# Mapping the risk distribution of *Borrelia burgdorferi* sensu lato in China from 1986 to 2020: a geospatial modelling analysis

**DOI:** 10.1080/22221751.2022.2065930

**Published:** 2022-04-12

**Authors:** Tian-Le Che, Bao-Gui Jiang, Qiang Xu, Yu-Qi Zhang, Chen-Long Lv, Jin-Jin Chen, Ying-Jie Tian, Yang Yang, Simon I. Hay, Wei Liu, Li-Qun Fang

**Affiliations:** a State Key Laboratory of Pathogen and Biosecurity, Beijing Institute of Microbiology and Epidemiology, Beijing, People’s Republic of China; b School of Mathematical Sciences, University of the Chinese Academy of Sciences, Beijing, People’s Republic of China; c Research Center on Fictitious Economy and Data Science, Chinese Academy of Sciences, Beijing, People’s Republic of China; d School of Economics and Management, University of the Chinese Academy of Sciences, Beijing, People’s Republic of China; e Department of Biostatistics, College of Public Health and Health Professions, and Emerging Pathogens Institute, University of Florida, Gainesville, FL, USA; f Department of Health Metrics Sciences, School of Medicine, University of Washington, Seattle, WA, USA; g Institute for Health Metrics and Evaluation, University of Washington, Seattle, WA, USA

**Keywords:** Lyme borreliosis, *Borrelia burgdorferi* sensu lato, modelling, BRT, spatial risk

## Abstract

Lyme borreliosis, recognized as one of the most important tick-borne diseases worldwide, has been increasing in incidence and spatial extent. Currently, there are few geographic studies about the distribution of Lyme borreliosis risk across China. Here we established a nationwide database that involved *Borrelia burgdorferi* sensu lato (*B. burgdorferi*) detected in humans, vectors, and animals in China. The eco-environmental factors that shaped the spatial pattern of *B. burgdorferi* were identified by using a two-stage boosted regression tree model and the model-predicted risks were mapped. During 1986−2020, a total of 2,584 human confirmed cases were reported in 25 provinces. *Borrelia burgdorferi* was detected from 35 tick species with the highest positive rates in *Ixodes granulatus*, *Hyalomma asiaticum*, *Ixodes persulcatus*, and *Haemaphysalis concinna* ranging 20.1%−24.0%. Thirteen factors including woodland, NDVI, rainfed cropland, and livestock density were determined as important drivers for the probability of *B. burgdorfer*i occurrence based on the stage 1 model. The stage 2 model identified ten factors including temperature seasonality, NDVI, and grasslands that were the main determinants used to distinguish areas at high or low-medium risk of *B. burgdorferi*, interpreted as potential occurrence areas within the area projected by the stage 1 model. The projected high-risk areas were not only concentrated in high latitude areas, but also were distributed in middle and low latitude areas. These high-resolution evidence-based risk maps of *B. burgdorferi* was first created in China and can help as a guide to future surveillance and control and help inform disease burden and infection risk estimates.

## Introduction

Lyme borreliosis, caused by the complex *Borrelia* (*B.*) *burgdorferi* sensu lato (hereinafter referred to as *B. burgdorferi*), is the most common tick-borne disease in the Northern Hemisphere[1,2], especially in the United States, with approximately 476,000 cases per year[3]. In European countries such as Netherlands, Austria, and Estonia, the annual incidence of Lyme borreliosis has reached >80 cases per 100,000 individuals [4]. *Borrelia burgdorferi* sensu lato infection in humans can cause skin, joints, heart, nervous system and other tissues and organs to be injured, leading to erythema migrans (EM), Lyme arthritis, meningo-radiculo-neuritis, acrodermatitis chronica atrophicans (ACA) and other clinical symptoms[1,2]. The main clinical features of Lyme borreliosis in North America and Europe are similar, however, there are more frequent systemic symptoms and seroreactivity in North America and more diverse clinical manifestations in Europe, such as ACA and borrelial lymphocytoma, which are not observed in North America[1,5]. Although more than 20 genospecies of *B. burgdorferi* complex have been found[6], there are three genospecies that most commonly cause Lyme borreliosis in humans: *B. afzelii*, *B. garinii* and *B. burgdorferi* sensu stricto, which are characterized by different vector competences, geographical distributions, and pathogenicity to humans[7]. In Europe, these three genospecies have been reported, and they are mainly transmitted by *Ixodes* (*I.*) *ricinus*[5,8]. *Borrelia burgdorferi* sensu stricto is more frequently reported in the United States, which is mainly spread by *I. scapularis* and *I. pacificus*[1]. In Asia, *B. afzelii* and *B. garinii* are predominant, while *B. burgdorferi* sensu stricto is rare, and the main vector is often *I. persulcatus* [8,9].

In China, since Lyme borreliosis was first reported in Hailin county in Heilongjiang Province[10], it has been found to be endemic in a large range of geographic regions, vectored mainly by *Ixodes* ticks, which maintain *B. burgdorferi* in a horizontal transmission cycle between ticks and multiple vertebrate hosts[11]. According to the most recent literature review in China, Lyme borreliosis is carried by at least 13 tick species[12]. Four genospecies of *B. burgdorferi* including *B. afzelii*, *B. garinii*, *B. burgdorferi* sensu stricto and *B. valaisiana-*related have been reported to cause Lyme borreliosis in China. *Borrelia garinii* is the most dominant genospecies, followed by *B. afzelii*, and the other two are much less frequent[1,13–16]. *Ixodes persulcatus* as well as various other *Ixodes* and *Haemaphysalis* species are the vectors of Lyme borreliosis in China[14,17]. Many studies have either predicted the distribution of vectors of *B. burgdorferi*, such as *I. persulcatus* and *I. granulatus*, through species distribution models, or focused on the investigations of *B. burgdorferi* infections in local areas[18–22]. But there are no studies about distributions and risk assessment of *B. burgdorferi* infections in humans, animals and vectors throughout the mainland of China which has hindered informed control strategies.

In this study, we established a comprehensive database on distributions of *B. burgdorferi* across the mainland of China during 1986−2020, with the goal of acquiring a better understanding of the epidemiology and distribution of Lyme borreliosis since its first identification in 1986. We also associated a variety of eco-environmental factors with the risk of *B. burgdorferi* infection, to build a comprehensive predictive model of the occurrence of *B. burgdorferi* infection in China.

## Method

### Data collection and management

We assembled five datasets, including four involving the occurrence of *B. burgdorferi* (i) among human confirmed cases, (ii) in human individuals with serological evidence, (iii) in animals (livestock and wild animals) with molecular, serological, or pathogen isolation evidence, (iv) in ticks with molecular, serological or pathogen isolation evidence, as well as one dataset related to the eco-environmental factors, which were chosen due to their potential effect on the ecological suitability of *B. burgdorferi* occurrence based upon the previous studies[23–25]. These included climate data from Worldclim, livestock density information from the Food and Agriculture Organization, mammalian richness from NASA, land cover, elevation, Normalized Difference Vegetation Index (NDVI, a proxy for the density of actively photosynthesising vegetation[26]), population density, and night-time light from China Resource and Environment Science and Data Center (Supplementary Tables 1 and 2).

**Figure 1.**
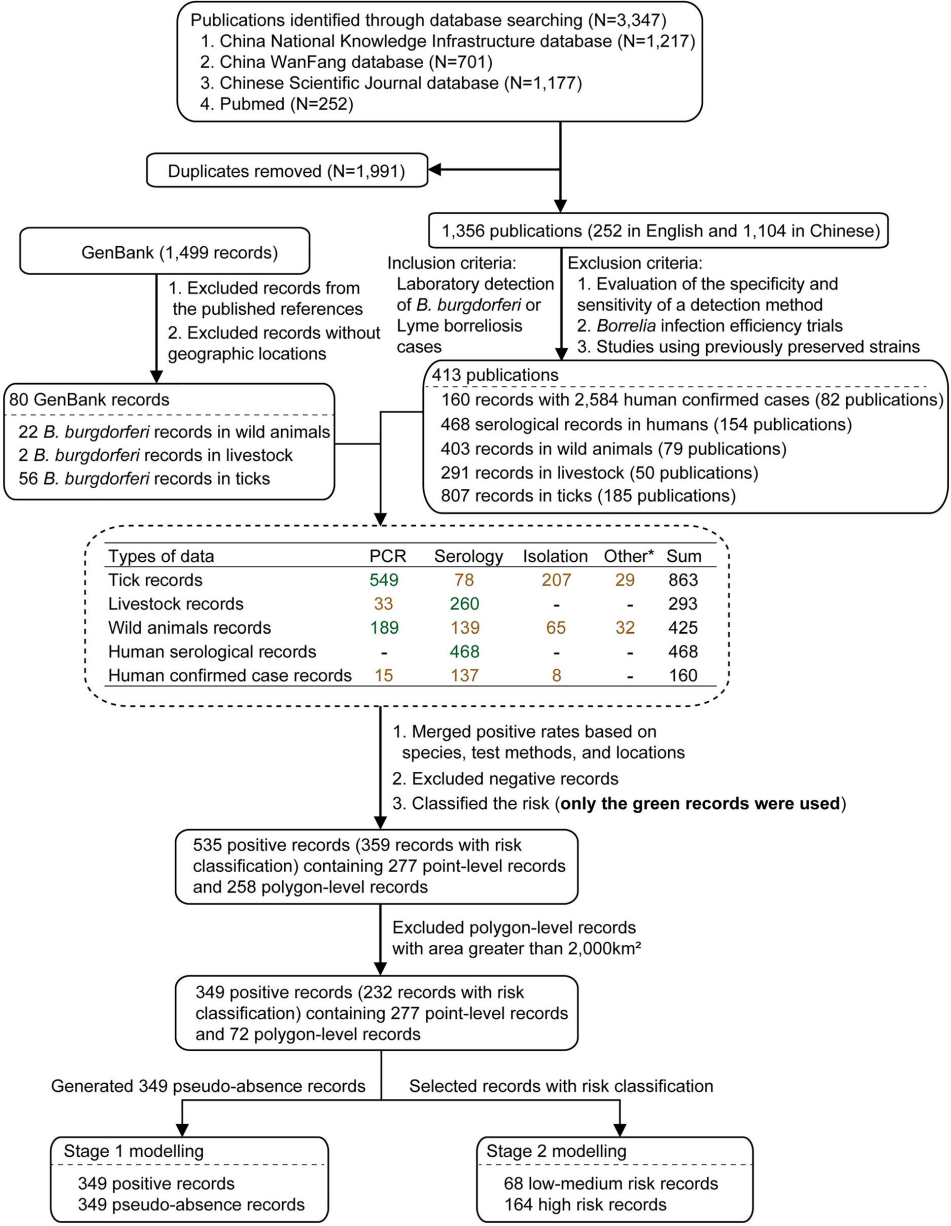
Flow chart of literature review. *Other test assays included RFLP (restriction fragment length polymorphism) and MLSA (multilocus sequence analysis).

Literature related to *B. burgdorferi* were searched from the major databases (PubMed, China National Knowledge Infrastructure, China WanFang database, and Chinese Scientific Journal database), with the keywords “Lyme” or *Borrelia burgdorferi* and “China.” All the publications between January 1986 and December 2020 were searched without language limitations. Studies were eligible if they described the laboratory detection of *B. burgdorferi* in humans, ticks, or animals by any of the molecular assay, serological assay, or isolation. We excluded the following studies: (i) evaluation of the specificity and sensitivity of a detection method; (ii) *B. burgdorferi* infection efficiency trials; and (iii) studies using previously preserved strains. In addition, unpublished *B. burgdorferi* data from GenBank (https://www.ncbi.nlm.nih.gov/genbank/) were supplemented (Figure 1).

Title and abstracts of the retrieved studies using the search strategy were screened independently by two reviewers (QX, BGJ) to identify studies potentially eligible for inclusion. The full texts of the potentially eligible studies were retrieved and independently assessed for eligibility by two reviewers (TLC, JJC). All conflicts of opinion and uncertainties were discussed and resolved by consensus with a third reviewer. Attempts were also made to clarify any uncertainties or missing data in selected reports with the corresponding authors. The main content of the database was to summarize the positive rates of *B. burgdorferi* for different hosts or vector ticks in different locations. The positive rates of multiple surveys were combined when the surveyed species of hosts or vector ticks and detection method of *B. burgdorferi* were same in the same locations, e.g. the overall positive rate of *B. burgdorferi* in *I. persulcatus* was calculated by overall number of positive detections divided by the number of *I. persulcatus* tested if multiple investigations of *I. persulcatus* were performed in the same place. Then locations with negative tests for *B. burgdorferi* were excluded. The detailed data extraction, variables definition, numbers of publications and records, and data used for the two-stage BRT model are shown in Figure 1 and Supplementary method.

### The risk classification of B. burgdorferi

A positive record was defined as one or more laboratory or clinically confirmed infections of *B. burgdorferi* in human beings, or *B. burgdorferi* detected from ticks or animals occurring at a unique location (the same administrative area or 10 km×10 km pixel for points). For each positive record, we used positive rates of *B. burgdorferi* in humans, livestock, wild animals, or ticks as proxies for the grading of the *B. burgdorferi* risk, and the positive rate was only valid when the samples tested were greater than 10. To ensure the homogeneity of these positive rates, we only included serological tests of livestock and humans (excluding investigations focusing on people with specific clinical conditions, such as psychosis and arthritis), PCR tests of ticks and wild animals for the risk classification since other methods had fewer records (Figure 1). For a specific sampling site where multiple tick species were tested, the highest positive rate of tick species was considered as the final positive rate. The same method was applied to wild animals and livestock. Positive records not used for risk classification were considered unknown risk, including human confirmed cases, ticks and wild animals not tested by PCR, livestock not tested by serology, and data with less than 10 tests (Figure 1).

Based on the final positive rates that were determined for each record, the 3rd quartile of each type of data (11% for humans, 17% for wild animals, 25% for livestock, and 24% in ticks) was used as a threshold to classify the risk for each of surveyed locations. Briefly, the surveyed locations were considered as with high-risk if (1) positive detection rate of *B. burgdorferi* by PCR higher than 24% in ticks or higher than 17% in wild animals; or (2) positive detection rate by serological assay higher than 11% in humans or higher than 25% in livestock. Otherwise, the low-medium risk classification was defined. Our goal was to obtain a comprehensive risk classification from different data sources, e.g. investigations of *B. burgdorferi* in ticks, wild animals, livestock and humans, because a single type of data was not enough to cover all regions. For those survey sites where they were more than one type of positive rate, the risk was determined based on the data type that produced the highest risk classification.

In addition, we selected pseudo-absence records considered as “controls” in the modelling analysis to indicate locations with no risk. The method was as follows: (1) Randomly sampled the pseudo-absence records from 10 km×10 km pixel map; (2) The sampling range was grids within 200 km of any occurrence record, but excluded grids within 20 km of any occurrence record. (3) The number of samples was the same as the occurrence records [27,28]. Finally, locations with 4 categories of *B. burgdorferi* risk were generated, including no risk (pseudo-absence records), medium-low risk (positive rate less than 3rd quartile), high risk (positive rate greater than 3rd quartile) and unknown risk.

### Model development

The BRT model is usually used for ecological niche modelling[29,30], similar to logistic regression, with a binary outcome as the dependent variable and environmental variables as the independent variable. A two-stage boosted regression tree (BRT) model was applied to identify the potential ecological drivers for distribution of *B. burgdorferi* and create a high-resolution risk map using the R package “dismo”[31] and “gbm”[32] based on the locations with 4 categories of *B. burgdorferi* risk. The Pearson correlation coefficient was used to screen variables to reduce multicollinearity, only one variable was kept when two variables were correlated (*r* > 0.75) (Supplementary Figure 1). Subsequently, we filtered out variables with a relative contribution of less than 4% by conducting a pre-model for each of the two-stage BRT models by including all the variables filtered by the multicollinearity test. Based on 13 screened variables including 5 eco-climatic variables, 4 land cover types, livestock, population density, NDVI, and elevation, the stage 1 model was used to distinguish whether there was a risk of *B. burgdorferi* occurrence. In this case, all locations with medium-low risk, high risk and unknown risk were considered as “cases,” and those with no risk (pseudo-absence records) were considered as “controls.” The stage 2 model further distinguished high or low-medium risk for areas at risk of *B. burgdorferi* occurrence projected by the stage 1 model, and 10 screened variables, including 2 eco-climatic variables, 5 land cover types, population density, NDVI, and mammalian richness were included in the stage 2 modelling analysis.

The positive rates of different sources may vary somewhat, e.g. a higher positive rate of ticks in a particular area did not necessarily mean a higher positive rate of humans. Therefore, we separately ran the models by including only human or tick data as sensitivity analyses in the stage 2 model, while separate stage 2 model was not performed by including only wild animals and livestock due to few available records with specific locations. In addition, the stage 1 model was conducted by including all types of data, rather than by including only one type of data, because any positive detection in humans, ticks or animals can indicate the presence of this pathogen. In addition, we also performed sensitivity analyses using the 72nd and 78th percentiles, respectively, as thresholds for risk classification in the stage 2 BRT model. The Kappa coefficient was calculated to assess the consistency of sensitivity analyses with the main result. Details about model development, screening of multicollinearity, cross-validation for tuning parameters, assessment of goodness-of-fit, and sensitivity analysis were given in the Supplementary method and Supplementary Figure 1.

All analysis was performed in R 3.6.3 (R Foundation for Statistical Computing, Vienna, Austria.) and ArcGIS 10.7 (ESRI Inc., Redlands, CA, USA).

## Results

### Database assembly

The literature review yielded a total of 1,356 publications. In addition, 1,499 records from GenBank were determined. With the consensus of two independent reviewers, 413 publications and 80 records from GenBank met the inclusion criteria and were used for data extraction. After pooling data from all sources, we assembled a comprehensive database of *B. burgdorferi* occurrence including 160 records reporting on the human confirmed cases, 468 records on serological tests in humans, and 293, 425, and 863 records in livestock, wild animals, and ticks, respectively (Figure 1).

### Human confirmed cases and serological investigation of B. burgdorferi

**Figure 2.**
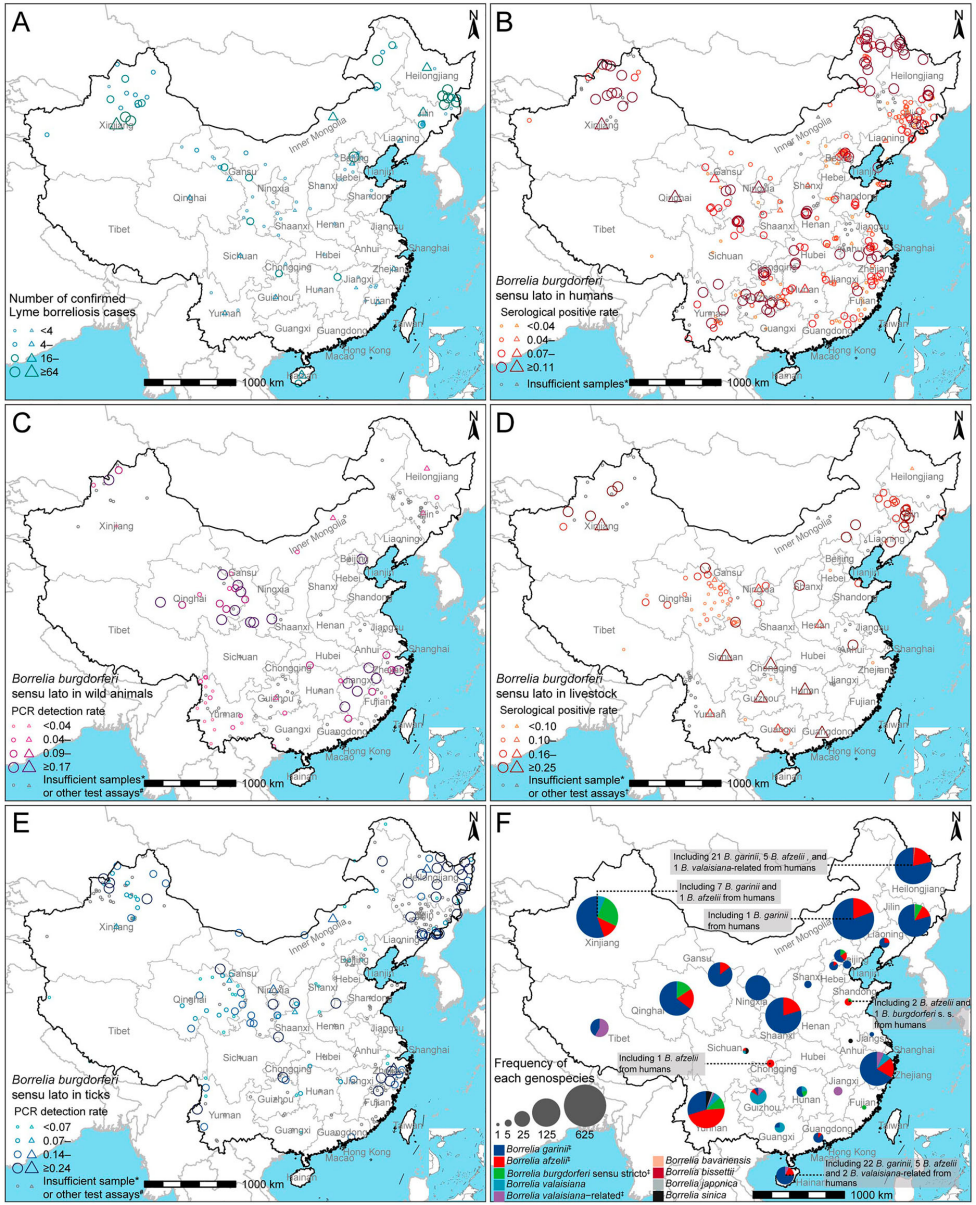
Distribution of Lyme borreliosis cases and the positive rate of *Borrelia burgdorferi* sensu lato. The quartiles of each type of data were used to truncate the corresponding data. **(A)** Distribution of confirmed cases of Lyme borreliosis. **(B)** Distribution of infection rate of specific antibody for *Borrelia burgdorferi* sensu lato in humans. **(C)** Distribution of detection rate for *Borrelia burgdorferi* sensu lato in wild animals. **(D)** Distribution of infection rate of specific antibody for *Borrelia burgdorferi* sensu lato in livestock. **(E)** Distribution of detection rate for *Borrelia burgdorferi* sensu lato in ticks. **(F)** Genospecies distribution of *Borrelia burgdorferi* sensu lato in China. Records reported at the province level were represented as a triangle, while records reported at the point, county, city-level were represented as a circle. *Positive rate was not calculated for the data with the number of samples tested less than 10. ^#^Other test assays included serological method, isolation, RFLP, and MLSA. ^†^Other test assays included PCR. ^‡^It has been isolated from patients.

A total of 2,584 confirmed cases were reported from 1986 to 2020 and were mainly located in the Northeast China (942 cases, 36.5%), Inner Mongolia-Xinjiang (1,034 cases, 40.0%) and North China regions (324 cases, 12.5%) (Figure 2A and Table 1). Based on available demographic data of cases, males accounted for 61.3% (1,013/1,653), and over half were aged 21–40 years (55.8%, 444/796), followed by 23.2% for 41–60 years olds and 17.5% for children and teenagers aged under 20 years. The age group over 60 had the lowest proportion (3.5%). Forest workers were the most frequent occupation of patients (39.7%, 305/768), followed by farmers and herdsmen (18.6%). Among 1,497 patients with known clinical symptoms, the most frequently seen were erythema migrans (44.7%), arthritis (24.4%), and neurologic manifestations (22.4%), while cardiac manifestations (5.3%), lymphadenopathy (2.2%), ocular manifestations (1.3%), and acrodermatitis chronica atrophicans (0.5%) were reported as the occasional complications. The incidence of erythema migrans in Northeast China attained as high as 60.6%, which differed from the South and the Qinghai-Tibet region of China, where arthritis was reported as the most common symptom (68.3% and 56.8%, respectively). A total of 468 records on serological surveys in human beings were reported from 154 publications, which resulted in an overall seroprevalence of 7.8% in healthy population, with particularly high seropositive rate in Xinjiang Uygur Autonomous Region (20.2%, 982/4,861) and Chongqing City (18.0%, 54/300), followed by Inner Mongolia Autonomous Region (14.2%, 729/5,133), Qinghai (12.6%, 373/2,969), Heilongjiang (11.4%, 911/7,965) and Hubei provinces (11.0%, 159/1,439) (Figure 2B).

**Table 1.** Demographic characteristics and clinical manifestations of Lyme borreliosis cases, 1986–2020, China

Characteristics	All	Northeast	North	Inner Mongolia-Xinjiang	Qinghai-Tibet	Southwest	Central	South
Overall	2,584	942	324	1,034	91	10	111	72
Sex*	1,653	751	136	645	74	3	6	38
Male	1,013 (61.3%)	456 (60.7%)	69 (50.7%)	422 (65.4%)	36 (48.6%)	3 (100.0%)	5 (83.3%)	22 (57.9%)
Female	640 (38.7%)	295 (39.3%)	67 (49.3%)	223 (34.6%)	38 (51.4%)	0 (0.0%)	1 (16.7%)	16 (42.1%)
Age*	796	487	82	106	74	3	7	37
<20	139 (17.5%)	81 (16.6%)	28 (34.1%)	20 (18.9%)	4 (5.4%)	0 (0.0%)	0 (0.0%)	6 (16.2%)
21–40	444 (55.8%)	320 (65.7%)	22 (26.8%)	49 (46.2%)	45 (60.8%)	2 (66.7%)	2 (28.6%)	4 (10.8%)
41–60	185 (23.2%)	85 (17.5%)	24 (29.3%)	34 (32.1%)	25 (33.8%)	0 (0.0%)	4 (57.1%)	13 (35.1%)
>60	28 (3.5%)	1 (0.2%)	8 (9.8%)	3 (2.8%)	0 (0.0%)	1 (33.3%)	1 (14.3%)	14 (37.8%)
Occupation*	768	571	82	68	0	1	6	40
Forest worker	305 (39.7%)	215 (37.7%)	29 (35.4%)	23 (33.8%)	0 (0.0%)	0 (0.0%)	6 (100.0%)	32 (80.0%)
Farmers and herdsmen	143 (18.6%)	95 (16.6%)	32 (39.0%)	13 (19.1%)	0 (0.0%)	1 (100.0%)	0 (0.0%)	2 (5.0%)
Others	320 (41.7%)	261 (45.7%)	21 (25.6%)	32 (47.1%)	0 (0.0%)	0 (0.0%)	0 (0.0%)	6 (15.0%)
Manifestations*	1,497	720	314	265	74	3	80	41
Erythema migrans	669 (44.7%)	436 (60.6%)	137 (43.6%)	68 (25.7%)	5 (6.8%)	0 (0.0%)	19 (23.7%)	4 (9.8%)
Arthritis	366 (24.4%)	122 (16.9%)	55 (17.5%)	86 (32.5%)	42 (56.8%)	1 (33.3%)	32 (40.0%)	28 (68.3%)
Neurologic manifestations	336 (22.4%)	100 (13.9%)	100 (31.8%)	97 (36.6%)	8 (10.8%)	2 (66.7%)	20 (25.0%)	9 (22.0%)
Cardiac manifestations	79 (5.3%)	42 (5.8%)	9 (2.9%)	7 (2.6%)	5 (6.8%)	0 (0.0%)	13 (16.2%)	3 (7.3%)
Lymphadenopathy	33 (2.2%)	27 (3.8%)	0 (0.0%)	2 (0.8%)	4 (5.4%)	0 (0.0%)	0 (0.0%)	0 (0.0%)
Ocular manifestations	20 (1.3%)	11 (1.5%)	3 (1.0%)	3 (1.1%)	2 (2.7%)	0 (0.0%)	0 (0.0%)	1 (2.4%)
ACA^#^	8 (0.5%)	1 (0.1%)	2 (0.6%)	4 (1.5%)	0 (0.0%)	0 (0.0%)	1 (1.2%)	0 (0.0%)
Other symptoms	174 (11.6%)	92 (12.8%)	29 (9.2%)	39 (14.7%)	9 (12.2%)	1 (33.3%)	2 (2.5%)	2 (4.9%)
Year	2,584	942	324	1,034	91	10	111	72
1986–1990	51 (2.0%)	9 (1.0%)	14 (4.3%)	28 (2.7%)	0 (0.0%)	0 (0.0%)	0 (0.0%)	0 (0.0%)
1991–1995	381 (14.7%)	123 (13.1%)	94 (29.0%)	100 (9.6%)	53 (57.9%)	1 (10.0%)	6 (5.4%)	4 (5.6%)
1996–2000	776 (30.1%)	251 (26.6%)	111 (34.4%)	328 (31.7%)	30 (33.3%)	0 (0.0%)	54 (48.6%)	2 (2.8%)
2001–2005	892 (34.5%)	355 (37.7%)	20 (6.1%)	467 (45.2%)	5 (5.1%)	5 (50.0%)	39 (35.4%)	1 (1.9%)
2006–2010	228 (8.8%)	82 (8.7%)	17 (5.2%)	110 (10.6%)	3 (3.7%)	3 (33.3%)	12 (10.5%)	1 (0.9%)
2011–2015	211 (8.2%)	122 (13.0%)	49 (15.1%)	0 (0.0%)	0 (0.0%)	1 (10.0%)	0 (0.0%)	39 (54.2%)
2016–2020	45 (1.7%)	0 (0.0%)	19 (5.9%)	1 (0.1%)	0 (0.0%)	0 (0.0%)	0 (0.0%)	25 (34.7%)

* Cases with incomplete characteristic information were excluded.

^#^ ACA: Acrodermatitis chronica atrophicans

The population of different regions are: Northeast (106 million); North (442 million); Inner Mongolia-Xinjiang (64 million); Qinghai-Tibet (13 million); Southwest (52 million); Central (550 million); South (148 million).

### B. burgdorferi in animals

A total of 403 records of *B. burgdorferi* in wild animals were reported, with an overall positive rate of 11.5% (945/8,226, 95% CI: 10.8%–12.2%) using PCR tests. The prevalence of *B. burgdorferi* varied by animal species when the study sites were combined, with the highest positive rate obtained from *Apodemus (A.) sylvaticus* (54.4%, 92/169) and followed by *A. peninsulae* (36.4%, 68/187) (Supplementary Table 3). A geographic difference existed, with the highest positive rates of *B. burgdorferi* infection among tested wild animals observed in Jiangxi Province (40.7%, 83/204) and in Tianjin City (32.0%, 32/100) (Figure 2C).

Altogether 293 records of *B. burgdorferi* in livestock were reported, which yielded an overall seroprevalence of 17.4% (2,727/15,667, 95% CI: 16.8%–18.0%). When disaggregated by livestock types, we observed a highly similar positive rate between goats (18.1%, 1,583/9,318), cattle (21.2%, 653/3,079), and horses (18.6%, 45/242), all higher than that observed in dogs (12.0%, 429/3,566) (Supplementary Table 3). Spatially, the highest positive rate of *B. burgdorferi* in livestock was recorded in Sichuan Province (62.1%, 64/103), followed by Xinjiang Uygur Autonomous Region (28.2%, 640/2 267) and Anhui Province (27.8%, 40/144) (Figure 2D).

### B. burgdorferi in ticks

**Figure 3.**
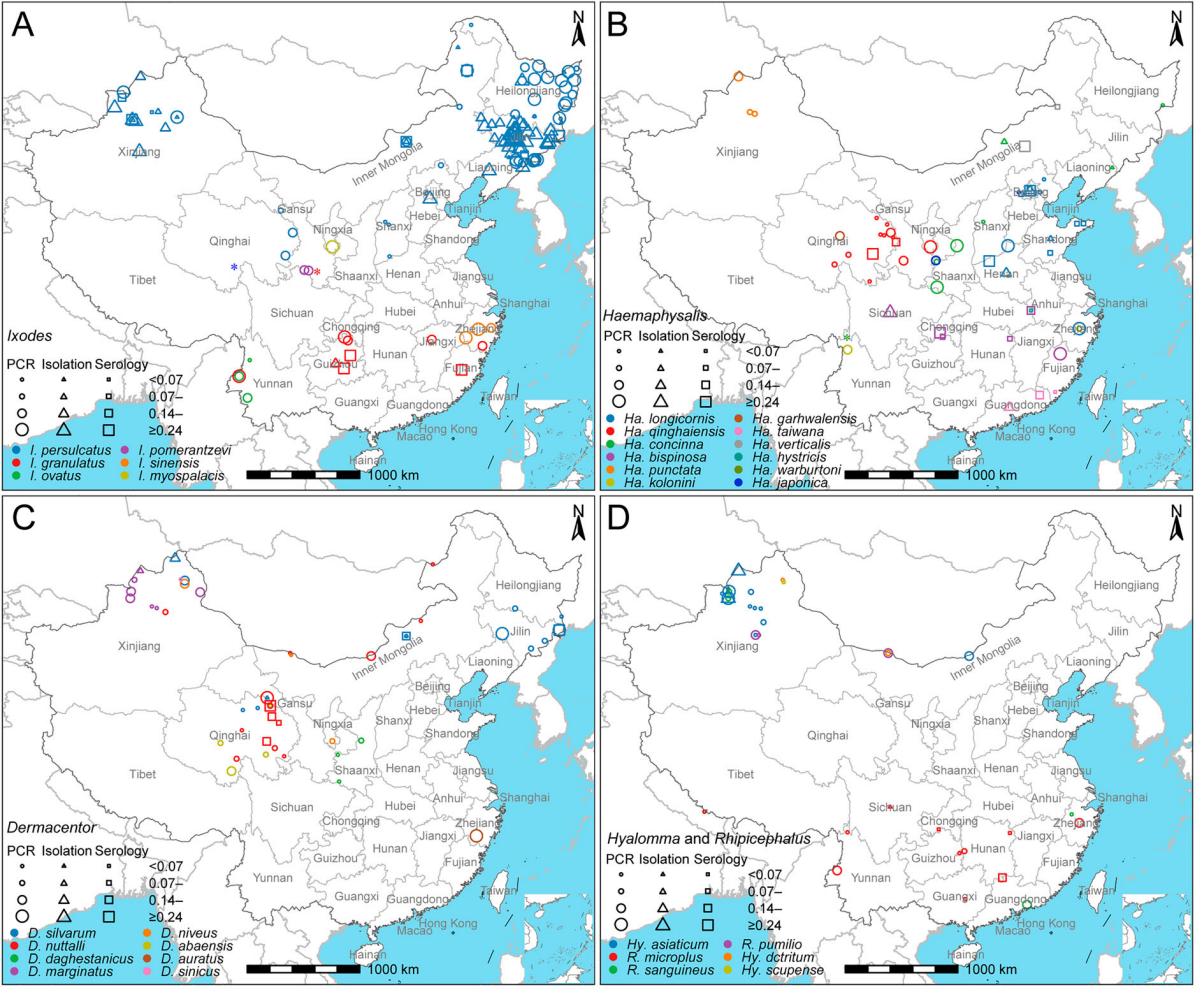
Distribution of the positive rate of *Borrelia burgdorferi* sensu lato detected in different tick species in China during 1986–2020. The quartiles of PCR positive rate in ticks were used to truncate the data. **(A)** *Ixodes* (*I.*). **(B)** *Haemaphysalis* (*Ha.*). **(C)** *Dermacentor* (*D.*). **(D)** *Hyalomma* (*Hy.*) and *Rhipicephalus* (*R.*). *The blue, red, and green asterisks represent *I. crenulatus*, *I. kuntzi* and *Ha. nepalensis*, respectively, and their positive rates were not calculated because the number of samples tested was less than 10.

A total of 863 records of *B. burgdorferi* in ticks were reported, yielding positive detection in 35 tick species, and negatives in 12 tick species (Figure 3 and Supplementary Table 4). The PCR prevalence of *B. burgdorferi* varied among the 35 tick species, with the high and comparable level attained among *I. granulatus* (24.0%, 92/384), *Hyalomma* (*Hy.*) *asiaticum* (22.0%, 304/1,379), *I. persulcatus* (20.7%, 1,821/8,777), and *Haemaphysalis* (*Ha.*) *concinna* (20.1%, 110/548), while low prevalence (less than 20%) obtained from other 31 tick species. The highest richness of tick species that were positive for *B. burgdorferi* (12 tick species out of 16 tick species) was observed in Xinjiang Uygur Autonomous Region. The highest number of tests and the highest positive rate (22.0%, 1,574/7,139) of *B. burgdorferi* in ticks were observed in Northeast China (Figure 2E, Figure 3).

### Genospecies of B. burgdorferi

A total of nine genospecies of *B. burgdorferi* were reported in the mainland of China (Figure 2F, Supplementary Table 5). *Borrelia garinii* was the most predominant genospecies, and had been reported in 21 provinces of China. *Borrelia afzelii* or *B. valaisiana* was the most prevalent genospecies in Southwest China such as Yunnan, Guizhou, Guangxi provinces, and Chongqing municipality. *Borrelia burgdorferi* sensu stricto was predominantly detected in Qinghai, Xinjiang, Yunnan, and Jilin provinces, and *B. valaisiana*-related was detected in Heilongjiang Province and seven provinces in South and Central China. Other genospecies such as *B. bavariensis*, *B. bissettii*, *B. japonica* and, *B. sinica* were detected only sporadically. Among them, *B. garinii*, *B. afzelii*, *B. burgdorferi* sensu stricto, and *B. valaisiana*-related have been detected in Lyme borreliosis patients, but only in Xinjiang, Heilongjiang, Inner Mongolia, Shandong, Chongqing, and Hainan provinces.

### The risk prediction of B. burgdorferi

**Figure 4.**
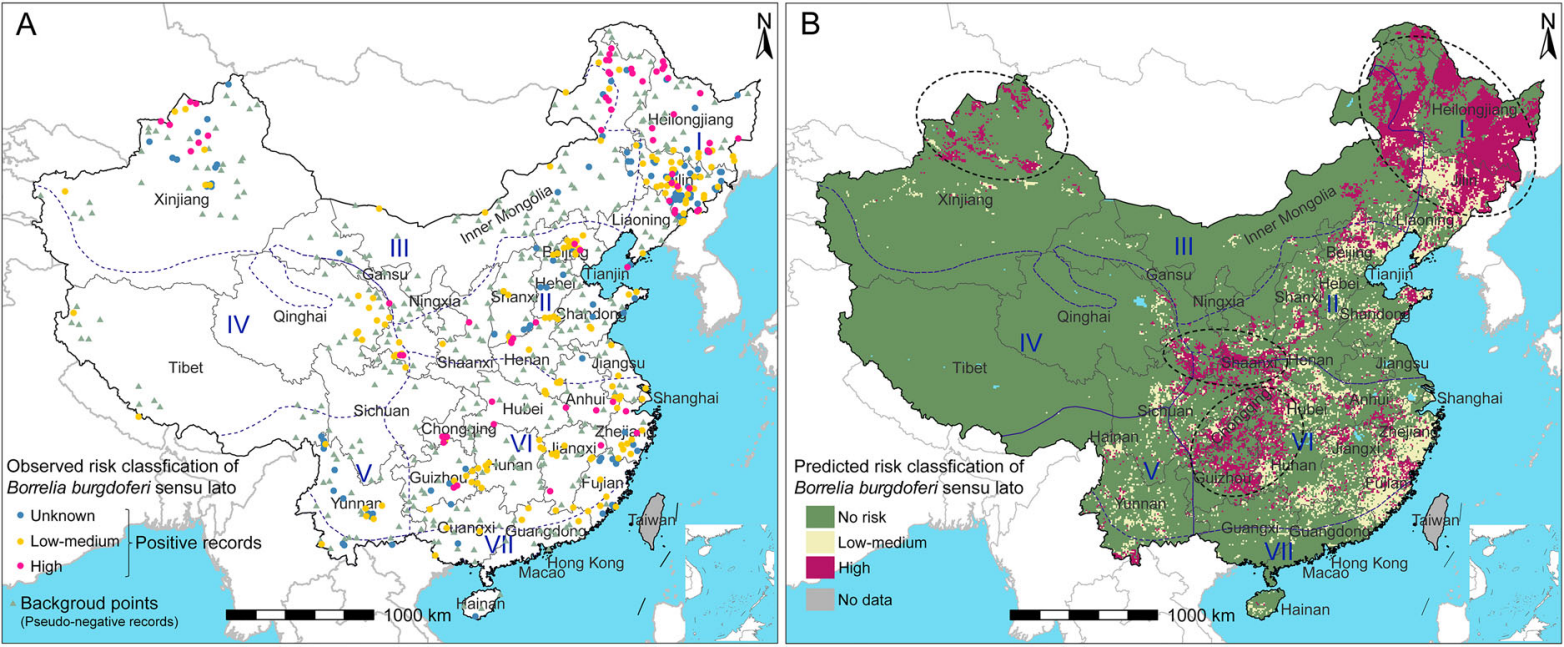
Recorded and predicted risk distribution of *Borrelia burgdorferi* sensu lato presence in China. **(A)** *Borrelia burgdorferi sensu lato* risk classification based on literature review. Background points sampled from the grid map based on distribution of positive records. The coordinates of polygon centroids were displayed for city-level or county-level evidence. **(B)** Predicted risk distribution of *Borrelia burgdorferi sensu lato* after averaged 100 bootstrapping BRT models. The thresholds of stage 1 and stage 2 model was determined by the cut-off values at which the Youden index of the test set was maximum. Black Doted Circles represent different high-risk hotspot areas. I = Northeast region, II = North China region, III = Inner Mongolia-Xinjiang region, IV = Qinghai-Tibet region, V = Southwest region, VI = Central China region, and VII = South China region.

According to the predictive model, four main high-risk areas for *B. burgdorferi* were defined that were centred around the mountains, i.e. Northeast China along the Changbai Mountains and the Greater Khingan Mountains; Inner Mongolia-Xinjiang region centreing around the Tianshan Mountains and the Altai Mountains; South, North China and Qinghai-Tibet region surrounding Qinling Mountains; Central China region, covering Guizhou Province, Chongqing and southwestern Hubei with the surrounding Wuling Mountains (Figure 4). In addition to the four hotspot areas, there were also scattered high-risk areas in northern Hebei Province, easternmost Shandong Province, southernmost Yunnan Province, southern Anhui Province, northern Fujian Province, and northern Jiangxi Province. The predicted *B. burgdorferi* risk area was wider than actual observed from the literature review based on the grid map, with the area at high-risk increased from 11,000 to 971,400 km^2^, the population size at high-risk increased from 1.4 to 116.6 million people, the area of low-medium risk increased from 25,100 to 820,100 km^2^, and the population size of low-medium risk increased from 5.1 to 251.0 million people (Table 2). Sensitivity analysis results showed that the mixed data model by including all of the human, tick and animal data had a substantial spatial similarity with the model of human data (kappa coefficient = 0.60, *P *< 0.001), and had a moderate spatial similarity with the model of tick data (kappa coefficient = 0.42, *P *< 0.001). However, in Jilin Province and Fujian Province, the human data model did not predict high risk and the mixed data model predicted high risk. At the same time, we also observed that the tick data model predicted many high-risk areas in these two provinces (Supplementary Figure 2). In addition, the prediction results using the 72nd percentile and the 78th percentile as risk classification thresholds were also highly similar to the final prediction results, with kappa coefficients of 0.73 and 0.75 (*P *< 0.001), respectively (Supplementary Figure 3).

**Table 2.** The area and population size of the *Borrelia burgdorferi sensu lato* occurrence risk predicted by the BRT model.

Region	BRT model predicted/Actual observed (Relative difference)			
	High risk area×10^3^ km^2^	Low-medium risk area×10^3^ km^2^	High risk population×10^3^ persons	Low-medium risk population×10^3^ persons
Northeast	394.0/4.1 (9,609.8%)	121.9/4.2 (2,902.4%)	29,899.4/345.4 (8,656.9%)	31,370.1/759.7 (4,129.5%)
North	86.8/0.8 (10,850.0%)	158.9/3.9 (4,074.4%)	19,589.6/221.9 (8,828.4%)	73,875.2/1,396.7 (5,289.4%)
Inner Mongolia-Xinjiang	177.1/2.0 (8,855.0%)	71.7/2.4 (2,987.5%)	5,157.9/46.5 (11,083.0%)	8,879.7/280.8 (3,162.0%)
Qinghai-Tibet	21.4/1.1 (1,945.5%)	27.3/4.3 (634.9%)	537.9/26.9 (1,997.0%)	952.3/123.6 (770.3%)
Southwest	11.3/0.3 (3,766.7%)	56.3/1.8 (3,127.8%)	1,363.8/37.9 (3,601.4%)	6,566.5/194.8 (3,370.1%)
Central	269.8/2.7 (9,992.6%)	335.5/7.2 (4,659.7%)	57,550.7/679.2 (8,473.4%)	110,563.5/1,757.8 (6,290.0%)
South	11.0/0.0 (-)	48.5/1.3 (3,730.8%)	2,501.6/0.0 (-)	18,804.1/544.8 (3,451.3%)
All	971.4/11.0 (8,830.9%)	820.1/25.1 (3,267.3%)	116,600.9/1,357.8 (8,587.4%)	251,011.5/5,058.2 (4,962.4%)

Note: The predicted risk was compared with the actual observed risk from literature review and the relative differences (%) are given in parentheses.

*In the observed data, unknown risk records were not included in the comparative analysis. For polygon data, the area and population were the average values of the grid it contains.

### Ecological niches suitable for B. burgdorferi distribution

In stage 1 of the BRT model, thirteen variables were determined as important drivers for distinguishing whether there was a risk of *B. burgdorferi* occurrence (Table 3 and Supplementary Figure 4). Six of them showed a positive correlation with the occurrence of *B. burgdorferi*, among which closed-canopy woodland has the largest relative contribution to the occurrence of *B. burgdorferi* (12.5%), followed by livestock density, population density, NDVI, other woodland, and rainfed cropland (Supplementary Figures 5−7). Three of them, the annual mean temperature, total precipitation, and isothermality, were negatively associated with *B. burgdorfer*i occurrence. Nonlinear effects were determined from the remaining variables, including temperature seasonality, elevation, precipitation seasonality, and shrub.

**Table 3.** The relative contribution of environmental variables to predict the occurrence risk of *Borrelia burgdorferi* sensu lato based on BRT model**.**

Stage 1			Stage 2		
Variable	Mean ± sd (%)	Effect	Variable	Mean ± sd (%)	Effect
Closed-canopy woodland	12.51 ± 1.89	Positive correlation	Temperature seasonality	22.08 ± 4.49	Positive correlation
Livestock	10.97 ± 1.25	Positive correlation	Isothermality	11.66 ± 2.10	Negative correlation
Temperature seasonality	10.15 ± 1.19	Nonlinear effects	High coverage grasslands	11.35 ± 2.77	Positive correlation
Annual mean temperature	8.43 ± 1.03	Negative correlation	Population density	10.50 ± 3.13	Nonlinear effects
Population density	7.80 ± 1.22	Positive correlation	NDVI	9.19 ± 2.14	Positive correlation
NDVI	7.27 ± 1.66	Positive correlation	Shrub	8.37 ± 2.13	Nonlinear effects
Other woodland	7.13 ± 1.05	Positive correlation	Mammalian richness	7.96 ± 1.86	Positive correlation
Total precipitation	6.84 ± 0.99	Negative correlation	Moderate coverage grasslands	6.78 ± 1.80	Positive correlation
Rainfed cropland	6.09 ± 1.15	Positive correlation	Sparse-canopy woodland	6.33 ± 2.17	Positive correlation
Elevation	6.08 ± 1.07	Nonlinear effects	Other woodland	5.78 ± 1.84	Nonlinear effects
Precipitation seasonality	5.92 ± 0.82	Nonlinear effects	–	–	–
Isothermality	5.58 ± 0.93	Negative correlation	–	–	–
Shrub	5.23 ± 0.93	Nonlinear effects	–	–	–

In stage 2 of the BRT model, ten predictors were determined to attain good performance in discriminating regions at high risk and low-medium risk of *B. burgdorferi* (Table 3 and Supplementary Figure 4). Temperature seasonality, high coverage grasslands, NDVI, mammalian richness, moderate coverage grasslands, and sparse-canopy woodland were positively related to increased risk of *B. burgdorferi*, while isothermality was negatively associated with the risk of *B. burgdorferi* (Supplementary Figures 8−10). Nonlinear effects were observed from population density, shrub, and other woodland. In the stage 2 model including only human data, climatic factors such as annual mean temperature and temperature seasonality were dominant factors, while vegetation-related variables such as NDVI and moderate coverage grasslands were the most important factors in the stage 2 model using only tick data (Supplementary Table 6).

The validation statistics showed that both stages of the BRT model have decent predictive performance, with AUC of 0.82 and 0.80 respectively. In addition, the sensitivity, specificity, accuracy, and F1 score also showed good performance of the model (Supplementary Figure 11 and Supplementary Table 7).

## Discussion

In China, evidence of Lyme borreliosis have been reported in many regions in recent years, while the disease burden and distribution of risk areas remained obscure, due to not being included in notifiable infectious diseases for surveillance. To our knowledge, this is the first systematic analysis of the appropriate niche of *B. burgdorferi* and risk mapping on distribution of *B. burgdorferi* in China. The demographic characteristics and clinical profile of Lyme borreliosis patients, the main tick vectors, animal hosts of *B. burgdorferi*, and distribution of *B. burgdorferi* genospecies in China, were comprehensively described.

Although our results showed a downward trend in the number of Lyme borreliosis cases in recent years, this did not reflect a reduction in the disease burden of Lyme borreliosis, but rather was an important piece of evidence that Lyme borreliosis has been neglected in China. The recent occurrence of emerging or reemerging tick-borne diseases with high disease severity have the possibility to led to the neglect of research on Lyme borreliosis in recent years, e.g. the main endemic areas of severe fever with thrombocytopenia syndrome (SFTS) in Central China region and of tick-borne encephalitis (TBE) in Northeast China region[33,34], have been shown a significant decrease of the reported number of Lyme borreliosis cases, while South China region with few endemic areas of SFTS and TBE was not shown a downward trend. In addition, the lag in publication of related papers may partly contribute to the perceived decline in the number of Lyme borreliosis cases.

Our results indicated that about 24% of Lyme borreliosis patients in China had arthritis, a late disease manifestation, which was much higher than the 2%–7% in Europe, although the main genospecies in China and Europe are the same[1]. Lack of early intervention and treatment for Lyme borreliosis might be the reason for the high number of arthritis symptoms in Lyme borreliosis cases in China[35], despite the therapeutic being widely available[36]. Almost 60% of the patients were forest workers, farmers and herdsmen, who are more exposed to tick bites during their professional activities. According to the studies in Europe, agricultural and forestry workers have a higher *B. burgdorferi* seroprevalence than general population, which indicates high proportion of forest workers, farmers and herdsmen could overestimate the seroprevalence in the population[23]. Therefore, we performed a comprehensive risk classification from different data sources as a proxy for risk at different locations, rather than directly using positive rates as an indicator of risk. Selecting tick species with the highest positive rate but with low population density as a proxy for *B. burgdorferi* risk assessment may overestimate the risk. Our modelling data showed that majority of tick species with the highest positive rate of *B. burgdorferi* infection were also the most predominant tick species in the regions [89.3% (42/47) locations], and the remaining 10.7% (5/47) locations, the tick species with the highest positive rate were also the subdominant tick species, which indicates a very low degree of overestimation for the risk estimation of *B. burgdorferi* infection in this study. In addition, livestock and wild animals are not the direct source of *B. burgdorferi* infection of humans or other animals, but only serve as a reference for the prevalence of *B. burgdorferi* in a region. Therefore, the species with the highest positive rate (usually the ones prone to tick bites) are more likely to reflect the actual prevalence of *B. burgdorferi* in this region.

Many tick species have been associated with Lyme borreliosis, but members of the genus *Ixodes* are considered the primary vectors and are the most common ticks known to transmit the pathogen to humans[1,2]. Among them, *I. persulcatus*, mainly distributed in northeastern and northwestern China, is considered to be the main vector of Lyme borreliosis in Asia and parts of Eastern Europe[7]. In southern China, another *Ixodes* tick with a high positive rate of *B. burgdorferi* and widespread distribution is *I. granulatus*[8], which is also considered to be a major vector of Lyme borreliosis in China[11]. These two *Ixodes* are thought to be predominantly found in forests, which often have environments suitable for high tick density[20,34]. When humans enter forest areas for agricultural or forestry activities such as mushroom picking and tree felling, they are easily exposed to these *Ixodes* ticks and infected with *B. burgdorferi*[34]. *Hyalomma asiaticum* and *Ha. concinna* also have high positive rates of *B. burgdorferi*. *Haemaphysalis concinna* is distributed in the northern and central regions, living in forests and shrubs and often parasitic on domestic animals, and the possibility of contact with rural residents is extremely high[37,38]. In a survey of Cangxi County, Sichuan Province, 53.8% of residents had been bitten by *Ha. concinna*[38]. *Hyalomma asiaticum* also frequently bite humans, given that it is the most important vector of Crimean-Congo hemorrhagic fever in China[39]. This tick is mainly found in the semi-desert areas of northwestern China[37,39]. A large number of forestry workers are in direct contact with this tick due to extensive afforestation activities[40]. Therefore, we speculated that these two ticks are also potential vectors of Lyme borreliosis. In addition, the difference in the positive rates of ticks in different regions was more likely to be caused by the tick species because the positive rates of *I. persulcatus* in Northeast China and the Xinjiang Uygur Autonomous Region were both high, while the positive rates of most other tick species in these two regions were low. The geographic difference in the positive rate of *B. burgdorferi* infection in wild animals is likely related to the composition of wild animals surveyed in different environments, given that wild animals usually affect tick populations and have different competencies to *B. burgdorferi* infection[41].

The prediction map of the BRT model showed that the potential risk area was much larger than the area observed in the survey, especially in the Central China region, which might be caused by insufficient surveillance. The four high-risk areas identified by this study surround four mountain areas, which were the Changbai and the Greater Khingan Mountains, Tianshan and Altai Mountains, Wuling Mountains, and Qinling Mountains. Previous studies have found that *I. persulcatus*, a tick species with a high infection rate of *B. burgdorferi*, is widely distributed in the first two high-risk areas[37], while two other tick species with high infection rates of *B. burgdorferi*, including *I. granulatus* and *Ha. concinna*, are widely located in the latter two high-risk areas[19,20]*.* Although featured by different ecological niches, all representing indicate higher environmental suitability for the propagation of tick vectors and wild animals. Vegetation-related variables such as NDVI and closed-canopy woodland were almost positively correlated at both stages of the model, which was in line with our expectations, as abundant vegetation is suitable for *B. burgdorferi* and its vectors and hosts[25,42]. In the stage 1 model, closed canopy woodland was the most important factor, indicating forests are important for *B. burgdorferi* to establish ecological cycles[25]. However, this factor was less important in the stage 2 model, possibly because more specific habitats including composition and population size of different vectors and animal hosts have a greater impact on whether there was a high risk of *B. burgdorferi*. Climate factors played a more important role in the model via only human data than the model that only via tick data. This might be due to the fact that different climatic regions have different ecological environments, which are closely related to human activities such as different types of agricultural production. Our previous studies have also indicated that agricultural production plays an important role in the occurrence of tick-borne diseases [33,34].

There are of course limitations to any ecological study of this kind. A report of absence may be not published due to the lack of positive results, and the efforts and quality of detection and report of *B. burgdorferi* infection can be variable by region. For example, a positive serological test can be due to a previous infection, and even nucleic acid testing methods may have errors, which can affect risk prediction[6]. Furthermore, the included data spanned a long duration of more than 30 years, with some of the historical documents inevitably suffering from flaws of changing criteria or standards that were applied in the curated data, all might influence the prediction of risk.

In conclusion, we applied an integrated approach to identify active *B. burgdorferi* foci in China. Compared with previous studies where *B. burgdorferi* hazard has been always evaluated using one single method and focusing on limited areas, this is the first attempt to adopt an integrated approach at the national level to include tick vectors, animals, and human beings. Areas predicted as suitable but with no records of disease might also be prudent to check for disease occurrence. Ultimately, to quantitatively assess the risk of Lyme borreliosis to humans, additional factors including density of predominant vector ticks, the bite rate and transmission capacity of vector ticks, the species and density of animal hosts, as well as the human behaviours and health care awareness, will be needed in future studies.

## Supplementary Material

Supplemental Material

## Data Availability

The datasets used and/or analyzed during the current study are available from the corresponding author on reasonable request.
