# Supplementary material for: Mapping the risk distribution of Borrelia burgdorferi sensu lato in China from 1986 to 2020: a geospatial modelling analysis
Source: Emerg Microbes Infect. 2022 Apr 12;11(1):1215–26. doi: 10.1080/22221751.2022.2065930 (PMC9067995; doi:10.1080/22221751.2022.2065930)
Supplement: Supplemental Material [file TEMI_A_2065930_SM2522.docx]

**Appendix:**

**Tian-Le Che, et al. Mapping the risk distribution of *Borrelia burgdorferi* sensu lato in China from 1986 to 2020: a geospatial modelling analysis**

**Contents**

[Supplementary Methods 3](#_Toc97711528)

[Supplementary Table 1. Information of data sources in this study. 8](#_Toc97711529)

[Supplementary Table 2. Description of 34 potential influencing factors used in the modelling efforts. 9](#_Toc97711530)

[Supplementary Figure 1. Correlation matrix for 34 variables. 10](#_Toc97711531)

[Supplementary Table 3. Mean prevalence rates of *Borrelia burgdorferi* sensu lato in animals. 11](#_Toc97711532)

[Supplementary Table 4. Mean prevalence rates of *Borrelia burgdorferi* sensu lato in ticks. 12](#_Toc97711533)

[Supplementary Table 5. Genospecies distribution of *Borrelia burgdorferi* sensu lato in China. 14](#_Toc97711534)

[Supplementary Figure 2. Predicted risk of *Borrelia burgdorferi* sensu lato in China using all positive records for stage 1 model and only human serological data or tick data for stage 2 model. 15](#_Toc97711535)

[Supplementary Figure 3. Predicted risk of *Borrelia burgdorferi* sensu lato in China based on different high-risk threshold. 16](#_Toc97711536)

[Supplementary Figure 4. Plots of all covariates entered into the BRT model. 17](#_Toc97711537)

[Supplementary Figure 5. The marginal effect of closed-canopy woodland, livestock, temperature seasonality, and annual mean temperature for *Borrelia burgdorferi* sensu lato risk in the stage 1 model. 18](#_Toc97711538)

[Supplementary Figure 6. The marginal effect of population density, NDVI, other woodland, and total precipitation for *Borrelia burgdorferi* sensu lato risk in the stage 1 model. 19](#_Toc97711539)

[Supplementary Figure 7. The marginal effect of rainfed cropland, elevation, precipitation seasonality, isothermality, and shrub for *Borrelia burgdorferi* sensu lato risk in the stage 1 model. 20](#_Toc97711540)

[Supplementary Figure 8. The marginal effect of temperature seasonality, isothermality, high coverage grasslands, and population density for *Borrelia burgdorferi* sensu lato risk in the stage 2 model. 21](#_Toc97711541)

[Supplementary Figure 9. The marginal effect of NDVI, shrub, mammalian richness, and moderate coverage grasslands for *Borrelia burgdorferi* sensu lato risk in the stage 2 model. 22](#_Toc97711542)

[Supplementary Figure 10. The marginal effect of sparse-canopy woodland and other woodland for *Borrelia burgdorferi* sensu lato risk in the stage 2 model. 23](#_Toc97711543)

[Supplementary Table 6. Relative contribution of environmental variables in the stage 2 model including only human or tick data. 24](#_Toc97711544)

[Supplementary Figure 11. Performance evaluation of the BRT model after averaged 100 bootstrapping models. 25](#_Toc97711545)

[Supplementary Table 7. Performance evaluation of binary BRT model. 26](#_Toc97711546)

[Supplementary References 27](#_Toc97711547)

Supplementary Methods

*Data extraction and variable definition*

A standardized form was used to enter the data from literature[[1-413](#_ENREF_1)] and GenBank that includes the following variables: year of publication, study year, study subjects (human beings, ticks, or animals), genospecies of *B. burgdorferi* and the number of the genospecies , number of samples tested and number of positive for *B. burgdorferi*, laboratory method (molecular, serological or isolation assay) used to identify *B. burgdorferi*, geo-coded study sites parameters, and the baseline information on the study subjects. For tested ticks, the specific species were collected. For tested animals, we divide them into livestock and wild animals, with the former divided into cattle, sheep, and others and the latter collecting detailed species. For the tested population, the categories of people (healthy individuals or individuals with specific clinical manifestations) were collected. For confirmed human cases, sex, age, occupation, the clinical manifestations if any were collected. According to the diagnosis standard issued by Chinese Preventive Medicine Association (http://www.ttbz.org.cn/Pdfs/Index/?ftype=st&pms=45652), a confirmed case was defined as (i) a history of tick bite or exposure history of ticks, (ii) any of the typical clinical symptoms (erythema migrans, arthritis, neurologic manifestations, cardiac manifestations, lymphadenopathy, ocular manifestations, acrodermatitis chronica atrophicans), and (iii) a positive test for *B. burgdorferi* (molecular test, serological test, or isolation).

After data collection was completed, all data were carefully checked, and duplicate data were removed. Then all data were geo-referenced to the highest level using Baidu Map (http://api.map.baidu.com/lbsapi/getpoint/), which generate point-level data at village, town, or hill level and polygon-level data at county, prefecture, or province level. We used ArcGIS 10.7 (Esri Inc, Redlands, CA, USA) to create a grid map with a spatial resolution of 10km*10km to associate each piece of data. Each point-level data was matched to a grid by latitude and longitude. Polygon-level data were matched to the corresponding administrative district map and were only used as a supplement for modelling when the point-level data in this area were not available. Multiple records with the same geographic location were merged and the number of tests and positives were summed when the species (tick species, wild animal species and, the categories of livestock) and detection method were the same. The polygon-level data with area more than 2000 km^2^ were excluded to reduce the bias of model caused by non-standard spatial scale [414]. A positive record at a specific sampling site was defined as human confirmed cases or any positive test for *B. burgdorferi* in humans, or animals, or ticks by any one of the methods involving molecular, serological, or isolation assay.

Data on the environmental, socioeconomic, and biological factors during the study period were collected in the form of raster map, including climate data, land cover, elevation, NDVI, livestock density, mammalian richness, population density, nighttime light (Supplementary Table 1). For land cover data, percentage coverage of paddy field, rainfed cropland, closed-canopy woodland, shrub, sparse-canopy woodland, other woodland, high coverage grasslands, moderate coverage grasslands, low coverage grasslands were extracted and summarized at the study levels (10km*10km resolution). For climatic data, 19 variables (Bio1‒19) were created based on monthly maximum temperature, monthly minimum temperature and monthly rainfall from 1986‒2018 using R package “dismo” in order to generate more biologically meaningful variables (Supplementary Table 2). The map in raster format was summarized into processable data format at the study level based on the grid map, and R package “Raster” was used for resampling in order to change the resolution of raster data. Specifically, for data other than land cover, we used the “aggregate” function to create a new Raster with a lower resolution, and then used the “projectRaster” function to project the values of environment variables to the grid map through bilinear interpolation method (the value of the four nearest input cell centers was used to determine the value on the output raster). But for land cover data, we first projected the “id” value (index of each grid) of the grid map to the land cover data using the nearest neighbor method, and then counted the number of pixels for each land use types corresponding to each grid (indexed by “id” value). Finally, the multi-year average of 34 covariates were calculated for each grid (Supplementary Table 2). Then we calculated the covariate values for all the locations of *B. burgdorferi* occurrence. The point data have the same value as its corresponding grid. For polygon data, the value of the covariable was the average of all the grids contained in the polygon[[414](#_ENREF_414)].

*Model development*

At stage 1, a BRT model with a logistic structure was used to analyze the relationship between the presence/absence of *B. burgdorferi* and socioeconomic, environmental, and climatic predictors. At stage 2, we built a BRT model with a logistic structure to fit the high risk/low-medium risk of *B. burgdorferi* on socio-environmental predictors.

To reduce multicollinearity among the predictors for the BRT modelling, we evaluated pairwise Pearson correlations between predictors (Supplementary Figure 1). For each pair of predictors with an absolute Pearson correlation coefficient higher than 0.75, only one predictor was selected in the analysis. We first selected the most two commonly used variables with easily interpretable biological significance, bio1 (annual average temperature) and bio12 (annual precipitation), and then excluded variables with correlation coefficients greater than 0.75 with these two variables. For the remaining pairs of correlated variables, we removed the variable with a greater correlation coefficient with other variables. In the end, a total of 19 variables including bio1, bio3, bio4, bio12, bio15, elevation, paddy field, rainfed cropland, closed-canopy woodland, shrub, sparse-canopy woodland, other woodland, high coverage grasslands, moderate coverage grasslands, low coverage grasslands, NDVI, livestock density, mammalian richness, and population density were retained.

In both models, we performed feature selection by selecting variables whose importance were greater than 4% after building the pre-model which included all predictors to reduce potential overfitting and improve the accuracy of models. In addition, a bootstrapping procedure with 100 iterations was conducted to generate more robust estimates. In each iteration, a training set with 80% of data was randomly selected by bootstrapping without replacement, and the remaining 20% served as a test set. The train set was used to train the BRT model, and the test set was used to evaluate model performance and generate the best threshold cutoff value based on the Youden index for the classification of predicted values.

**Sensitivity analyses**

Data from different sources were inevitably heterogeneous, e.g., a higher positive rate of ticks in a particular area does not necessarily mean a higher positive rate of humans. Therefore, in the stage 2 BRT model, we separately ran the models for humans and ticks as sensitivity analyses, but not for wild animals and livestock due to few available records in wild animals and livestock. In other words, we only used tick or human data in the stage 2 model, however the stage 1 model still used all types of data because we believe that any positive detection in humans, ticks or animals can indicate the presence of this pathogen in vectors and host. In addition, we also performed sensitivity analyses using the 72^nd^ and 78^th^ percentiles, respectively, as thresholds for risk classification in the stage 2 BRT model. In the above two sensitivity analyses, the stage 1 model was not changed. The kappa coefficient was calculated to assess the consistency of sensitivity analyses with the main result.

*Risk assessment*

After the locations from literature review were mapped to a 10×10km grid, we calculated the grid population and area corresponding to different risk levels as the actual observed risk. We calculated the grid population and area corresponding to the different risks of the predicted results of the two-stage BRT model as the predicted risk. We also calculated the relative difference between the predicted risk and the actual observed risk.

Supplementary Table 1. Information of data sources in this study.

| Variable | Source | Note | Data period |
| --- | --- | --- | --- |
| Climate data | Fick S E, Hijmans R J. WorldClim 2: new 1‐km spatial resolution climate surfaces for global land areas. International journal of climatology, 2017, 37(12): 4302-4315.  Harris I, Jones P D, Osborn T J, et al. Updated high‐resolution grids of monthly climatic observations–the CRU TS3. 10 Dataset. International journal of climatology, 2014, 34(3): 623-642. (available at: https://www.worldclim.org/data/monthlywth.html) | Including monthly minimum temperature, maximum temperature, and precipitation with a resolution of 2.5 min of arc. | 1986–2018 |
| Land cover | Institute of Geographic Sciences and Natural Resources Research, Cas. Remote sensing monitoring data of Land cover in China. Beijing: Resource and Environment Science and Data Center. (available at: https://www.resdc.cn/Datalist1.aspx?FieldTyepID=1,3) | A raster digital map with a resolution of 1km. | 1980–2018 |
| Elevation | Institute of Geographic Sciences and Natural Resources Research, Cas. DEM (Digital elevation model) of China. Beijing: Resource and Environment Science and Data Center. (available at: https://www.resdc.cn/data.aspx?DATAID=123) | A raster digital map with a resolution of 1km. | 2000 |
| NDVI | Institute of Geographic Sciences and Natural Resources Research, Cas. Annual 1km spatial distribution of normalized difference vegetation index (NDVI) in China. Beijing: Resource and Environment Science and Data Center. (available at: https://www.resdc.cn/data.aspx?DATAID=257) | On the basis of monthly data, the annual vegetation index dataset was generated by the maximal synthesis method; a resolution of 1km. | 1998–2018 |
| Livestock density | Gilbert M, Nicolas G, Cinardi G, et al. Global cattle distribution in 2010 (5 minutes of arc). V3 ed: Harvard Dataverse; 2018. (available at: https://doi:10.7910/DVN/GIVQ75)  Gilbert M, Nicolas G, Cinardi G, et al. Global goats distribution in 2010 (5 minutes of arc). V3 ed: Harvard Dataverse; 2018. (available at: https://doi:10.7910/DVN/OCPH42)  Gilbert M, Nicolas G, Cinardi G, et al. Global buffaloes distribution in 2010 (5 minutes of arc). V3 ed: Harvard Dataverse; 2018. (available at: https://doi:10.7910/DVN/5U8MWI)  Gilbert M, Nicolas G, Cinardi G, et al. Global sheep distribution in 2010 (5 minutes of arc). V3 ed: Harvard Dataverse; 2018. (available at: https://doi:10.7910/DVN/BLWPZN) | Including the density of cattle, buffalo, sheep, and goat with a resolution of 5 min of arc. | 2010 |
| Mammalian richness | International Union for Conservation of Nature - IUCN, Center for International Earth Science Information Network - CIESIN - Columbia University. Gridded Species Distribution: Global Mammal Richness Grids, 2015 Release. Palisades, NY: NASA Socioeconomic Data and Applications Center (SEDAC); 2015. (available at: https://sedac.ciesin.columbia.edu/data/set/species-global-mammal-richness-2015) | Provide information on the number of mammal species present at a 1km spatial resolution. | 2013 |
| Population density | Institute of Geographic Sciences and Natural Resources Research, Cas. Population spatial distribution kilometer grid dataset of China. Beijing: Resource and Environment Science and Data Center. (available at: https://www.resdc.cn/data.aspx?DATAID=251) | A raster digital map with a resolution of 1km. | 1995–2015 |
| Night-time Light | Institute of Geographic Sciences and Natural Resources Research, Cas. Global nighttime light data 2000-2013. Beijing: Resource and Environment Science and Data Center. (available at: https://www.resdc.cn/data.aspx?DATAID=213) | A raster digital map with a resolution of 1km. | 2000-2013 |

Supplementary Table 2. Description of 34 potential influencing factors used in the modelling efforts.

| **Variable** | **Description** |
| --- | --- |
| Bio1 | Annual average temperature (℃) |
| Bio2 | Mean diurnal range (Mean of monthly max - min temperature) (℃) |
| Bio3 | Isothermality (BIO02 ÷ BIO07 ×100) (%) |
| Bio4 | Temperature seasonality (standard deviation of mean temperature over 12 months of a year) |
| Bio5 | Max temperature of warmest month (℃) |
| Bio6 | Min temperature of coldest month (℃) |
| Bio7 | Annual range of temperature (BIO05-BIO06) (℃) |
| Bio8 | Mean temperature of wettest quarter (℃) |
| Bio9 | Mean temperature of driest quarter (℃) |
| Bio10 | Mean temperature of warmest quarter (℃) |
| Bio11 | Mean temperature of coldest quarter (℃) |
| Bio12 | Annual precipitation (mm) |
| Bio13 | Precipitation of wettest month (mm) |
| Bio14 | Precipitation of driest month (mm) |
| Bio15 | Precipitation seasonality (coefficient of variation in rainfall over 12 months of the year) |
| Bio16 | Precipitation of wettest quarter (mm) |
| Bio17 | Precipitation of driest quarter (mm) |
| Bio18 | Precipitation of warmest quarter (mm) |
| Bio19 | Precipitation of coldest quarter (mm) |
| Elevation | Average altitude (m) |
| Paddy field | Percentage coverage of paddy field (%) |
| Rainfed cropland | Percentage coverage of rainfed cropland (%) |
| Closed-canopy woodland | Percentage Coverage of Closed woodland (%) |
| Shrub | Percentage Coverage of shrub (%) |
| Sparse-canopy woodland | Percentage Coverage of sparse woodland (%) |
| Other woodland | Percentage Coverage of other woodland (%), contains young afforested land, cut-over land, nursery garden, and garden plot. |
| High coverage grasslands | Percentage coverage of high coverage grasslands (%) |
| Moderate coverage grasslands | Percentage coverage of moderate coverage grasslands (%) |
| Low coverage grasslands | Percentage coverage of low coverage grasslands (%) |
| NDVI | Normalized Difference vegetation Index |
| livestock density^*^ | Density of livestock (1 head per km²) |
| Mammalian richness | The number of mammal species per km² |
| Population density | Human population density (1 person per km^2^) |
| Nighttime light | Nighttime light index |

^*^Computed by sum of cattle density, buffalo density, goat density, and sheep density.

Supplementary Figure 1. Correlation matrix for 34 variables. Heatmap representing the correlation between features using Pearson correlation coefficient. Red and blue colors in the plot represent the positive and negative monotonic relationship respectively. The absolute value of the correlation coefficient was represented by the size of the circle, and the exact value shown in the lower triangle. The variables marked red represent those excluded by multicollinearity screening.


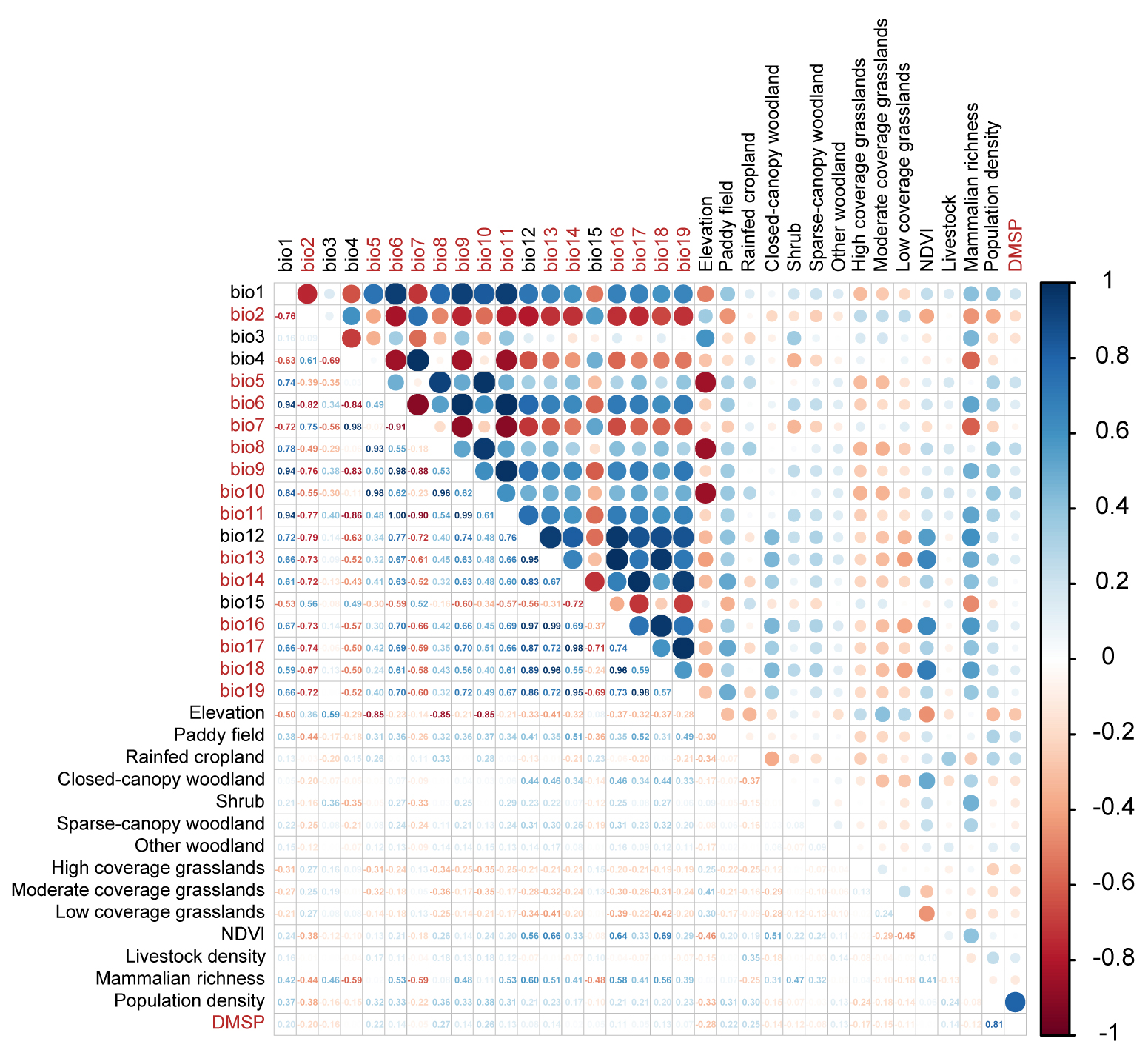


Supplementary Table 3. Mean prevalence rates of *Borrelia burgdorferi* sensu lato in animals.

|  | **PCR** | **Serology** | **Culture** |
| --- | --- | --- | --- |
| **Livestock** |  |  |  |
| Goat | 13.0 (19/146) | 18.1 (1583/8743) | - |
| Cattle | 1.5 (6/402) | 21.2 (653/3079) | - |
| Horse | 3.0 (1/33) | 18.6 (45/242) | - |
| Dog | 8.3 (43/520) | 12.0 (429/3566) | - |
| Deer | 25.4 (18/71) | - | - |
| Camel | - | 40.0 (10/25) | - |
| Fox | - | 58.3 (7/12) | - |
| **Wild animal** |  |  |  |
| *Apodemus agrarius* | 6.8 (13/191) | 40.7 (11/27) | 9.1 (1/11) |
| *Apodemus chevrieri* | 4.1 (9/219) | - | - |
| *Apodemus draco* | 4.2 (1/24) | - | - |
| *Apodemus orestes* | 30.0 (3/10) | - | - |
| *Apodemus peninsulae* | 36.4 (68/187) | - | 100.0 (2/2) |
| *Apodemus sylvaticus* | 54.4 (92/169) | - | 35.0 (7/20) |
| *Apodemus uralensis* | - | - | 55.6 (5/9) |
| *Berylmys bowersi* | 12.1 (4/33) | - | - |
| *Clethrionomys frater* | - | - | 27.7 (13/47) |
| *Clethrionomys rufocanus* | 9.1 (4/44) | - | 28.6 (2/7) |
| *Cricetulus barabensis* | - | 6.2 (1/16) | - |
| *Cricetulus longicaudatus* | 6.1 (2/33) | - | 100.0 (1/1) |
| *Cricetulus migratorius* | 11.1 (1/9) | - | - |
| *Ellobius talpinus* | - | - | 60.0 (3/5) |
| *Eothenomys miletus* | 0.6 (1/165) | - | - |
| *Lepus sinensis* | - | - | 100.0 (1/1) |
| *Meriones libycus* | 4.3 (2/46) | - | - |
| *Meriones unguiculatus* | 4.8 (6/124) | - | - |
| *Microtus arvalis* | - | - | 21.2 (11/52) |
| *Niviventer confucianus* | 16.4 (93/567) | - | 50.0 (1/2) |
| *Niviventer coninga* | 2.1 (1/48) | - | - |
| *Niviventer coxingi* | - | 50.0 (1/2) | 50.0 (1/2) |
| *Niviventer fulvescens* | 4.9 (2/41) | - | - |
| *Ochotona curzoniae* | 5.3 (4/75) | - | - |
| *Rattus losea* | 9.7 (9/93) | 20.0 (1/5) | - |
| *Rattus norvegicus* | 10.0 (3/30) | 20.0 (7/35) | 4.8 (1/21) |
| *Rattus tanezumi* | 14.5 (10/69) | 33.3 (2/6) | - |
| *Rhombomys opimus* | 24.0 (24/100) | - | - |
| *Sicista concolor* | - | - | 3.8 (2/52) |
| *Spermophilus undulatus* | 10.9 (24/220) | - | - |
| *Suncus murinus* | 25.0 (1/4) | - | - |
| *Tamias sibricus* | 12.5 (1/8) | - | - |

Supplementary Table 4. Mean prevalence rates of *Borrelia burgdorferi* sensu lato in ticks.

| Ticks | PCR | Serology | Culture | Genospecies of *Borrelia burgdorferi* sensu lato |
| --- | --- | --- | --- | --- |
| *Amblyomma testudinarium* | 0.0 (0/3) | - | - | *-* |
| *Arguas reflexus* | - | 4.2 (2/48) | - | Uncharacterised *Borrelia burgdorferi* sensu lato |
| *Dermacentor abaensis* | 10.8 (23/213) | - | - | Uncharacterised *Borrelia burgdorferi* sensu lato |
| *Dermacentor auratus* | 35.7 (20/56) | 0.0 (0/5) | - | Uncharacterised *Borrelia burgdorferi* sensu lato |
| *Dermacentor daghestanicus* | 3.9 (29/747) | - | - | Uncharacterised *Borrelia burgdorferi* sensu lato |
| *Dermacentor marginatus* | 16.5 (42/254) | - | 1.7 (2/118) | Uncharacterised *Borrelia burgdorferi* sensu lato |
| *Dermacentor niveus* | 6.4 (23/361) | - | 0.0 (0/14) | *Borrelia garinii, Borrelia afzelii* |
| *Dermacentor nuttalli* | 10.3 (149/1443) | 22.7 (25/110) | 0.0 (0/20) | *Borrelia garinii, Borrelia afzelii, Borrelia burgdorferi* sensu stricto*, Borrelia valaisiana, Borrelia bissettii* |
| *Dermacentor pavlovskyi* | 0.0 (0/1) | - | - | *-* |
| *Dermacentor silvarum* | 14.3 (175/1225) | 28.0 (104/372) | 6.5 (12/185) | *Borrelia garinii, Borrelia afzelii, Borrelia burgdorferi* sensu stricto*, Borrelia bavariensis, Borrelia bissettii* |
| *Dermacentor sinicus* | 6.7 (2/30) | - | - | Uncharacterised *Borrelia burgdorferi* sensu lato |
| *Haemaphysalis bispinosa* | 32.4 (11/34) | 14.6 (39/267) | 29.4 (20/68) | *Borrelia garinii, Borrelia valaisiana* |
| *Haemaphysalis concinna* | 20.1 (110/548) | - | 2.7 (11/403) | *Borrelia garinii, Borrelia afzelii, Borrelia burgdorferi* sensu stricto*, Borrelia bissettii* |
| *Haemaphysalis cornigera* | 0.0 (0/161) | - | 0.0 (0/10) | *-* |
| *Haemaphysalis erinacei* | 0.0 (0/15) | - | - | *-* |
| *Haemaphysalis garhwalensis* | 14.5 (22/152) | - | - | *Borrelia garinii, Borrelia afzelii* |
| *Haemaphysalis hystricis* | - | 12.5 (10/80) | - | Uncharacterised *Borrelia burgdorferi* sensu lato |
| *Haemaphysalis japonica* | 8.6 (5/58) | - | - | *Borrelia garinii, Borrelia afzelii* |
| *Haemaphysalis kolonini* | 19.0 (31/163) | - | - | Uncharacterised *Borrelia burgdorferi* sensu lato |
| *Haemaphysalis longicornis* | 9.9 (68/687) | 26.8 (158/590) | 12.4 (62/500) | *Borrelia garinii, Borrelia afzelii, Borrelia valaisiana, Borrelia valaisiana*-related |
| *Haemaphysalis nepalensis* | 33.3 (3/9) | - | - | Uncharacterised *Borrelia burgdorferi* sensu lato |
| *Haemaphysalis punctata* | 4.9 (9/182) | - | 0.0 (0/1) | *Borrelia garinii, Borrelia afzelii, Borrelia burgdorferi* sensu stricto*, Borrelia bissettii* |
| *Haemaphysalis qinghaiensis* | 15.0 (167/1114) | 23.9 (28/117) | - | *Borrelia garinii, Borrelia afzelii, Borrelia burgdorferi* sensu stricto |
| *Haemaphysalis taiwana* | 0.0 (0/14) | - | - | *-* |
| *Haemaphysalis tibetensis* | 0.0 (0/4) | - | - | *-* |
| *Haemaphysalis verticalis* | - | 11.1 (10/90) | 16.0 (8/50) | Uncharacterised *Borrelia burgdorferi* sensu lato |
| *Haemaphysalis warburtoni* | 12.5 (5/40) | - | - | Uncharacterised *Borrelia burgdorferi* sensu lato |
| *Hyalomma asiaticum* | 22.0 (304/1379) | - | 11.4 (24/210) | *Borrelia garinii, Borrelia burgdorferi* sensu stricto*, Borrelia valaisiana,* |
| *Hyalomma dctritum* | 3.5 (17/479) | - | 0.0 (0/63) | *Borrelia garinii, Borrelia afzelii* |
| *Hyalomma scupense* | 5.2 (4/77) | - | - | Uncharacterised *Borrelia burgdorferi* sensu lato |
| *Ixodes acutitarsus* | 0.0 (0/9) | - | - | *-* |
| *Ixodes crenulatus* | 33.3 (1/3) | - | - | Uncharacterised *Borrelia burgdorferi* sensu lato |
| *Ixodes granulatus* | 24.0 (92/384) | 57.1 (40/70) | 4.0 (12/300) | *Borrelia afzelii, Borrelia valaisiana, Borrelia japonica, Borrelia valaisiana*-related |
| *Ixodes haemaphysaloides* | 0.0 (0/1) | - | - | *-* |
| *Ixodes kashmiricus* | 0.0 (0/1) | - | - | *-* |
| *Ixodes kuntzi* | 50.0 (2/4) | - | - | Uncharacterised *Borrelia burgdorferi* sensu lato |
| *Ixodes myospalacis* | 45.5 (5/11) | - | - | Uncharacterised *Borrelia burgdorferi* sensu lato |
| *Ixodes ovatus* | 10.5 (34/323) | - | 0.0 (0/2) | *Borrelia garinii, Borrelia afzelii, Borrelia burgdorferi* sensu stricto*, Borrelia sinica* |
| *Ixodes persulcatus* | 20.7 (1821/8777) | 29.7 (328/1106) | 30.5 (2854/9359) | *Borrelia garinii, Borrelia afzelii, Borrelia bavariensis, Borrelia bissettii* |
| *Ixodes pomerantzevi* | 21.3 (13/61) | - | - | Uncharacterised *Borrelia burgdorferi* sensu lato |
| *Ixodes sinensis* | 16.4 (27/165) | - | 9.5 (2/21) | *Borrelia afzelii, Borrelia valaisiana* |
| *Ixodes vespertilionis* | 0.0 (0/4) | - | - | *-* |
| *Rhipicephalus haemaphysaloides* | 0.0 (0/1) | - | - | *-* |
| *Rhipicephalus microplus* | 7.3 (62/848) | 4.4 (8/183) | 1.6 (3/183) | *Borrelia garinii, Borrelia afzelii* |
| *Rhipicephalus pumilio* | 13.8 (113/817) | - | - | Uncharacterised *Borrelia burgdorferi* sensu lato |
| *Rhipicephalus sanguineus* | 9.8 (87/888) | - | - | *Borrelia garinii, Borrelia burgdorferi* sensu stricto |
| *Rhipicephalus turanicus* | 0.0 (0/100) | - | - | *-* |

Supplementary Table 5. Genospecies distribution of *Borrelia burgdorferi* sensu lato in China.

| **Province** | ***B. garinii*** | | |  | ***B. afzelii*** | | |  | ***B. valaisiana*** | | |  | ***B. burgdorferi* sensu stricto** | | |  | ***B. valaisiana*-related** | | |  | ***B. bavariensis*** | | |  | ***B. bissettii*** | | |  | ***B. japonica*** | | |  | ***B. sinica*** | | |
| --- | --- | --- | --- | --- | --- | --- | --- | --- | --- | --- | --- | --- | --- | --- | --- | --- | --- | --- | --- | --- | --- | --- | --- | --- | --- | --- | --- | --- | --- | --- | --- | --- | --- | --- | --- |
|  | Animals | Humans | Ticks |  | Animals | Humans | Ticks |  | Animals | Humans | Ticks |  | Animals | Humans | Ticks |  | Animals | Humans | Ticks |  | Animals | Humans | Ticks |  | Animals | Humans | Ticks |  | Animals | Humans | Ticks |  | Animal | Human | Tick |
| Anhui | - | - | - |  | - | - | - |  | - | - | - |  | - | - | - |  | - | - | - |  | - | - | - |  | - | - | - |  | - | - | - |  | - | - | 1 |
| Beijing | 8 | - | - |  | 1 | - | 2 |  | - | - | - |  | - | - | 2 |  | - | - | - |  | - | - | - |  | - | - | - |  | - | - | - |  | - | - | - |
| Fujian | - | - | - |  | - | - | - |  | - | - | - |  | - | - | 1 |  | - | - | - |  | - | - | - |  | - | - | - |  | - | - | - |  | - | - | - |
| Gansu | 14 | - | 45 |  | 2 | - | 8 |  | - | - | - |  | - | - | - |  | - | - | - |  | - | - | - |  | - | - | - |  | - | - | - |  | - | - | - |
| Guangdong | 1 | - | 6 |  | 1 | - | - |  | - | - | - |  | - | - | - |  | - | - | - |  | - | - | - |  | - | - | - |  | - | - | - |  | - | - | - |
| Guangxi | 2 | - | - |  | - | - | - |  | 6 | - | - |  | - | - | - |  | - | - | - |  | - | - | - |  | - | - | - |  | - | - | - |  | - | - | - |
| Guizhou | 2 | - | - |  | 2 | - | - |  | 5 | - | 12 |  | - | - | - |  | 3 | - | - |  | - | - | - |  | - | - | - |  | - | - | - |  | - | - | - |
| Hainan | - | 22 | - |  | - | 5 | - |  | - | - | - |  | - | - | - |  | - | 2 | - |  | - | - | - |  | - | - | - |  | - | - | - |  | - | - | - |
| Hebei | - | - | 5 |  | - | - | 1 |  | - | - | - |  | - | - | - |  | - | - | - |  | - | - | - |  | - | - | - |  | - | - | - |  | - | - | - |
| Heilongjiang | 9 | 21 | 201 |  | - | 5 | 53 |  | - | - | - |  | - | - | 1 |  | - | 1 | - |  | - | - | 1 |  | - | - | 2 |  | - | - | - |  | - | - | - |
| Hunan | - | - | 5 |  | - | - | - |  | 1 | - | 1 |  | 2 | - | - |  | - | - | - |  | - | - | - |  | - | - | - |  | - | - | - |  | - | - | - |
| Inner Mongolia | 19 | 1 | 393 |  | 1 | - | 98 |  | - | - | - |  | - | - | - |  | - | - | - |  | - | - | - |  | - | - | - |  | - | - | - |  | - | - | - |
| Jiangsu | 1 | - | - |  | - | - | - |  | - | - | - |  | - | - | - |  | - | - | - |  | - | - | - |  | - | - | - |  | - | - | - |  | - | - | - |
| Jiangxi | - | - | - |  | - | - | - |  | - | - | - |  | - | - | - |  | 3 | - | 3 |  | - | - | - |  | - | - | - |  | - | - | - |  | - | - | - |
| Jilin | 19 | - | 128 |  | - | - | 23 |  | - | - | - |  | 1 | - | 13 |  | - | - | - |  | - | - | 1 |  | - | - | - |  | - | - | - |  | - | - | - |
| Liaoning | 1 | - | 5 |  | - | - | 2 |  | - | - | - |  | - | - | - |  | - | - | - |  | - | - | - |  | - | - | - |  | - | - | - |  | - | - | - |
| Ningxia | - | - | 74 |  | - | - | - |  | - | - | - |  | - | - | - |  | - | - | - |  | - | - | - |  | - | - | - |  | - | - | - |  | - | - | - |
| Qinghai | 34 | - | 111 |  | 24 | - | 20 |  | - | - | 2 |  | - | - | 33 |  | - | - | - |  | - | - | - |  | - | - | - |  | - | - | - |  | - | - | - |
| Shaanxi | - | - | 218 |  | - | - | 56 |  | - | - | - |  | - | - | - |  | - | - | - |  | - | - | - |  | - | - | - |  | - | - | - |  | - | - | - |
| Shandong | - | - | - |  | - | 2 | 1 |  | - | - | - |  | - | 1 | - |  | - | - | - |  | - | - | - |  | - | - | - |  | - | - | - |  | - | - | - |
| Shanxi | 1 | - | 3 |  | - | - | - |  | - | - | - |  | - | - | - |  | - | - | - |  | - | - | - |  | - | - | - |  | - | - | - |  | - | - | - |
| Sichuan | - | - | - |  | 1 | 1 | 5 |  | 1 | - | - |  | - | - | - |  | - | - | - |  | - | - | - |  | - | - | - |  | - | - | - |  | 2 | - | - |
| Tianjin | 5 | - | - |  | - | - | - |  | - | - | - |  | - | - | - |  | - | - | - |  | - | - | - |  | - | - | - |  | - | - | - |  | - | - | - |
| Tibet | - | - | 13 |  | - | - | - |  | - | - | - |  | - | - | - |  | - | - | 18 |  | - | - | - |  | - | - | - |  | - | - | - |  | - | - | - |
| Xinjiang | 21 | 7 | 282 |  | 10 | 1 | 58 |  | 30 | - | 2 |  | 42 | - | 95 |  | - | - | - |  | - | - | - |  | 1 | - | - |  | - | - | - |  | - | - | - |
| Yunnan | 42 | - | 50 |  | 129 | - | 14 |  | 1 | - | 17 |  | 31 | - | 1 |  | 5 | - | - |  | - | - | - |  | - | - | - |  | 2 | - | 1 |  | - | - | 16 |
| Zhejiang | 52 | - | 91 |  | - | - | 41 |  | 8 | - | 8 |  | - | - | - |  | 14 | - | 1 |  | - | - | - |  | - | - | - |  | - | - | - |  | - | - | - |

Supplementary Figure 2. Predicted risk of *Borrelia burgdorferi* sensu lato in China using all positive records for stage 1 model and only human serological data or tick data for stage 2 model. **(A)** Predicted distribution of risk of *Borrelia burgdorferi* sensu lato using **human serological** data. **(B)** Predicted distribution of risk of *Borrelia burgdorferi* sensu lato using **tick** data. I =Northeast district, II= North China district, III = Inner Mongolia-Xinjiang district, IV = Qinghai-Tibet district, V= Southwest district, VI = Central China district, and VII= South China district. ^*^Positive data that did not participate in risk classification.


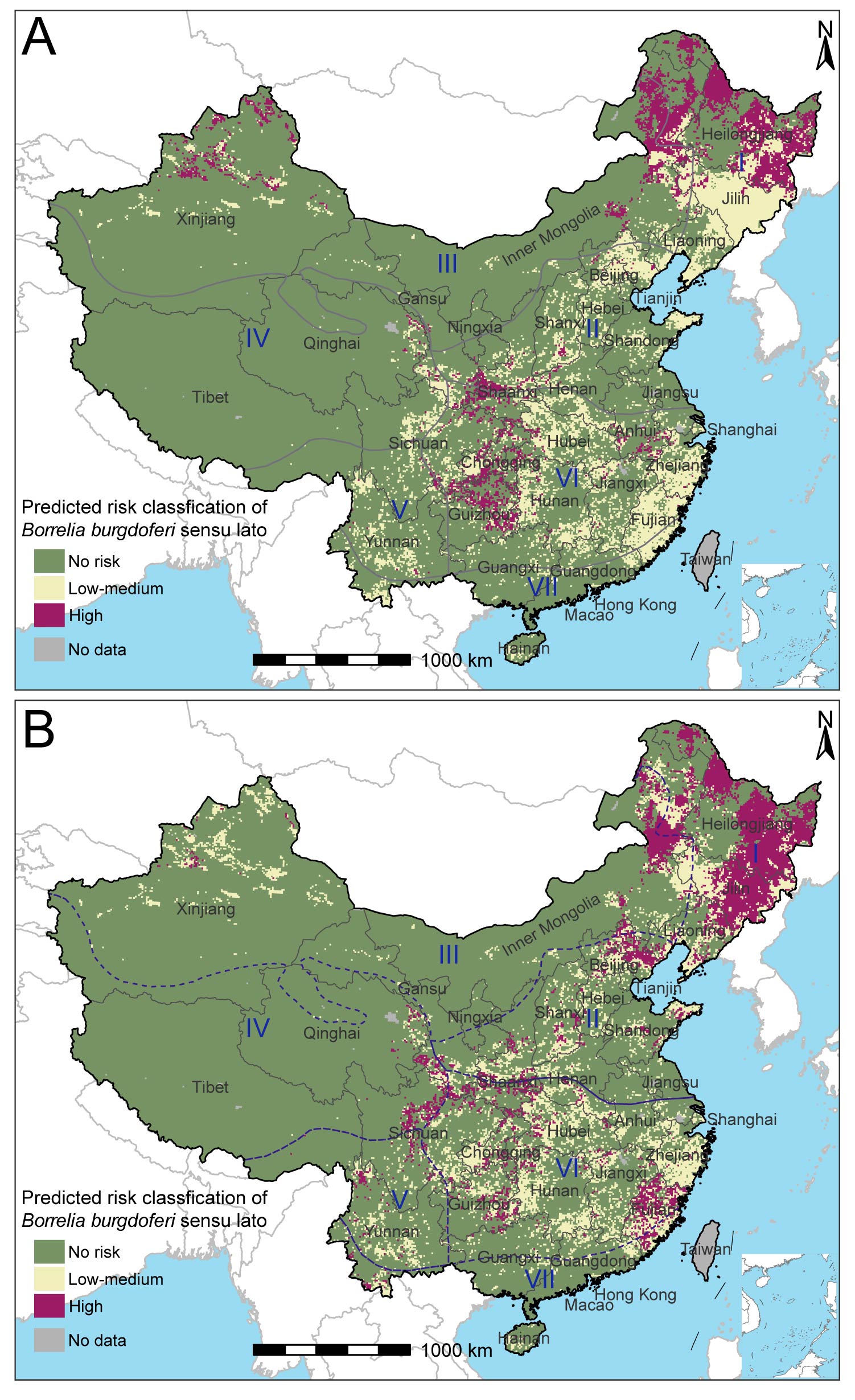


Supplementary Figure 3. Predicted risk of *Borrelia burgdorferi* sensu lato in China based on different high-risk threshold. **(A)** Predicted distribution of risk of *Borrelia burgdorferi* sensu lato using **72^nd^** percentile as high-risk threshold. **(B)** Predicted distribution of risk of *Borrelia burgdorferi* sensu lato using **78^th^** percentile as high-risk threshold. I =Northeast district, II= North China district, III = Inner Mongolia-Xinjiang district, IV = Qinghai-Tibet district, V= Southwest district, VI = Central China district, and VII= South China district. ^*^Positive data that did not participate in risk classification.


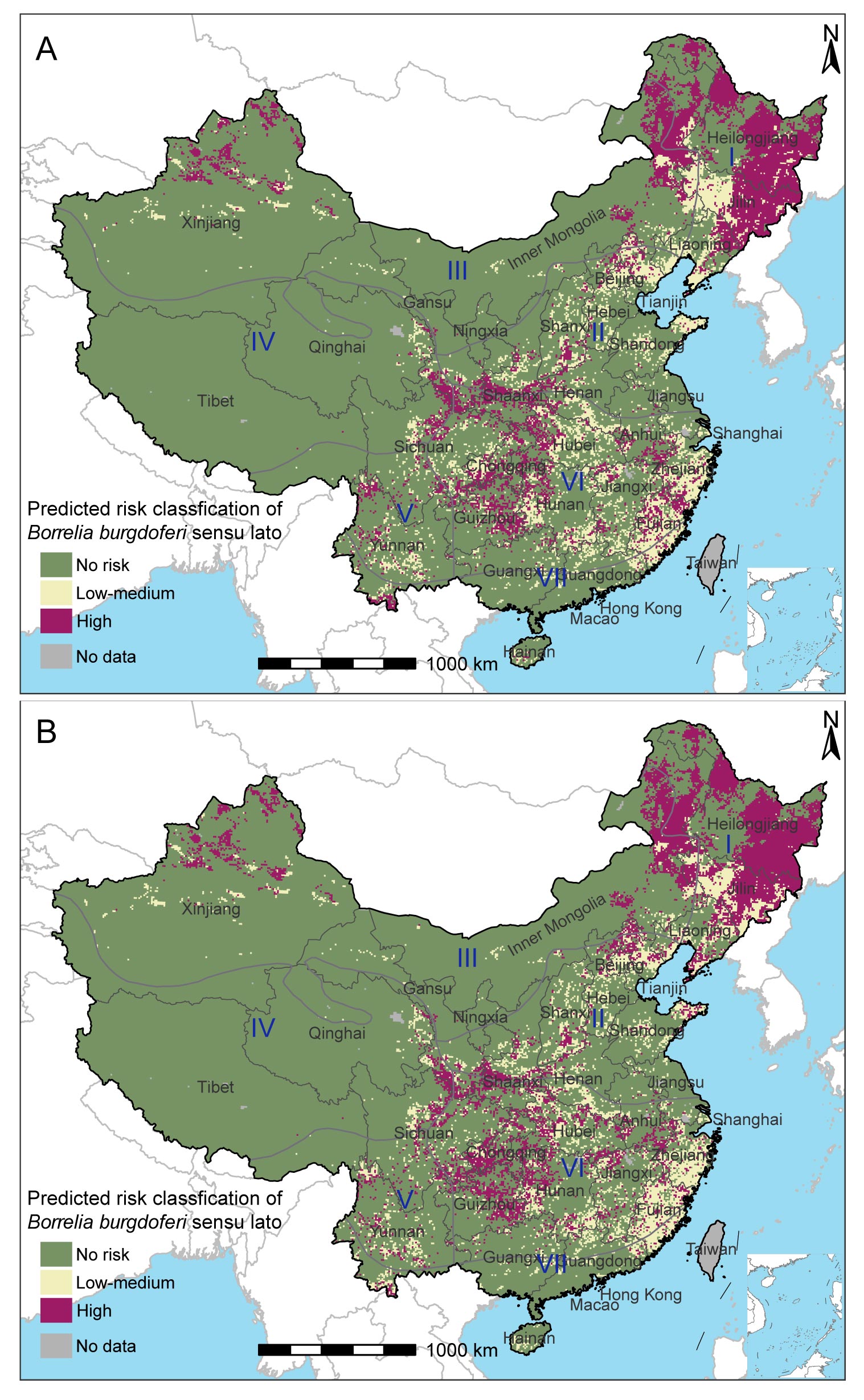


Supplementary Figure 4. Plots of all covariates entered into the BRT model.


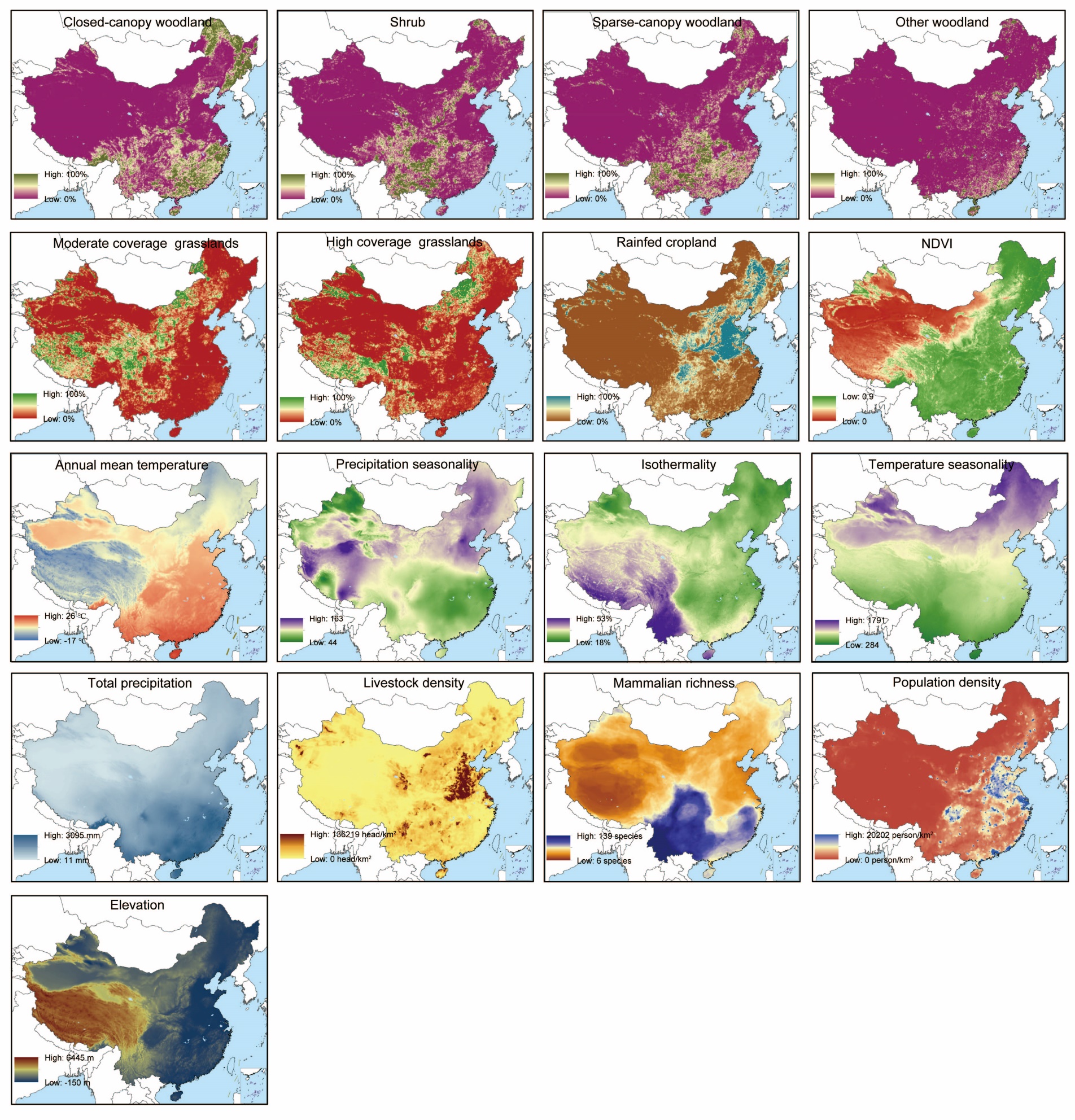


Supplementary Figure 5. The marginal effect of closed-canopy woodland, livestock, temperature seasonality, and annual mean temperature for *Borrelia burgdorferi* sensu lato risk in the stage 1 model. The red curves and gray bands show the average and range, respectively, of predicted occurrence risk from 100 BRT model using bootstrap method. Frequency distributions of the predictors are shown by the histograms in blue. The percentage values in parentheses show the relative contributions averaged over all 100 BRT models.


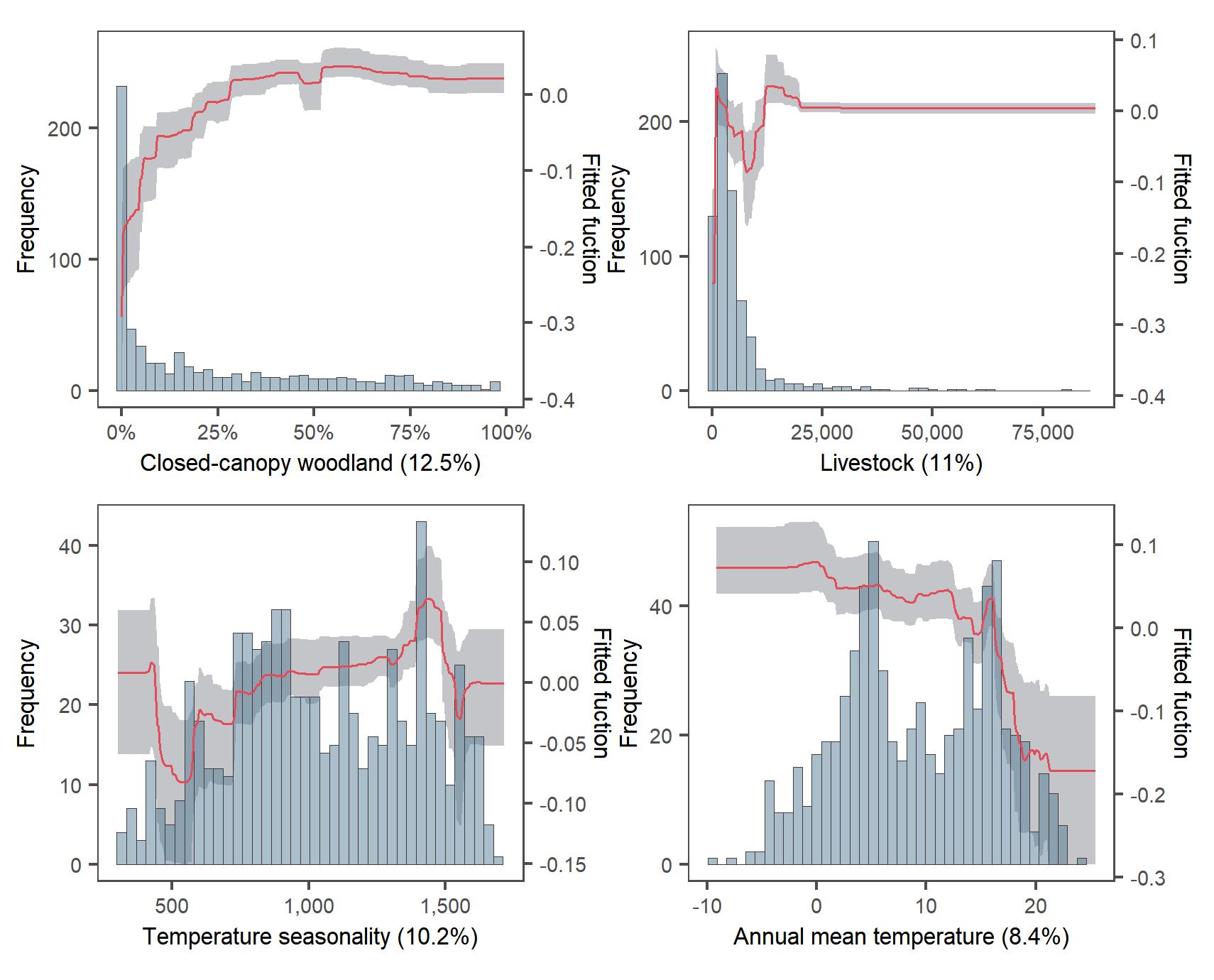


Supplementary Figure 6. The marginal effect of population density, NDVI, other woodland, and total precipitation for *Borrelia burgdorferi* sensu lato risk in the stage 1 model. The red curves and gray bands show the average and range, respectively, of predicted occurrence risk from 100 BRT model using bootstrap method. Frequency distributions of the predictors are shown by the histograms in blue. The percentage values in parentheses show the relative contributions averaged over all 100 BRT models.


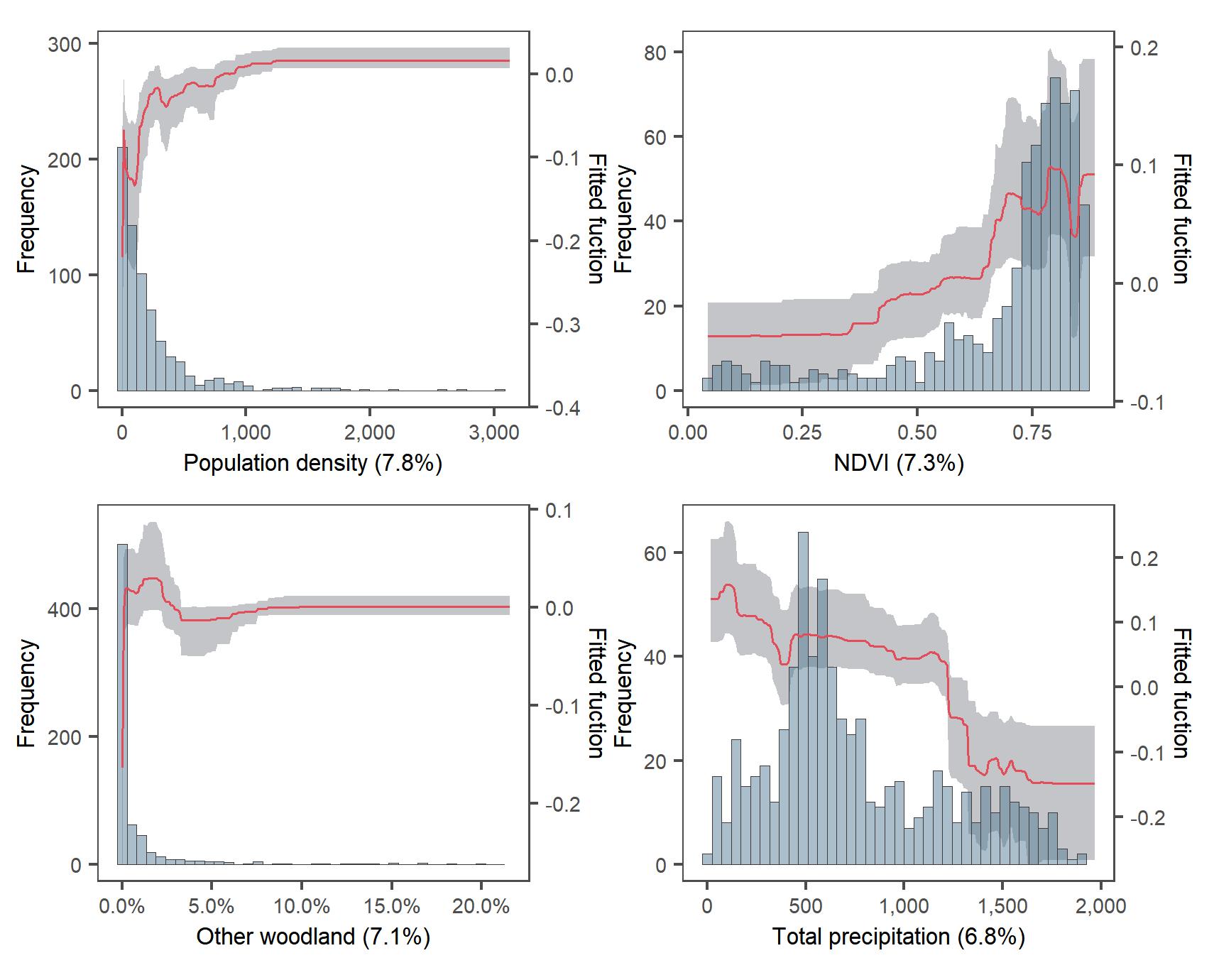


Supplementary Figure 7. The marginal effect of rainfed cropland, elevation, precipitation seasonality, isothermality, and shrub for *Borrelia burgdorferi* sensu lato risk in the stage 1 model. The red curves and gray bands show the average and range, respectively, of predicted occurrence risk from 100 BRT model using bootstrap method. Frequency distributions of the predictors are shown by the histograms in blue. The percentage values in parentheses show the relative contributions averaged over all 100 BRT models.


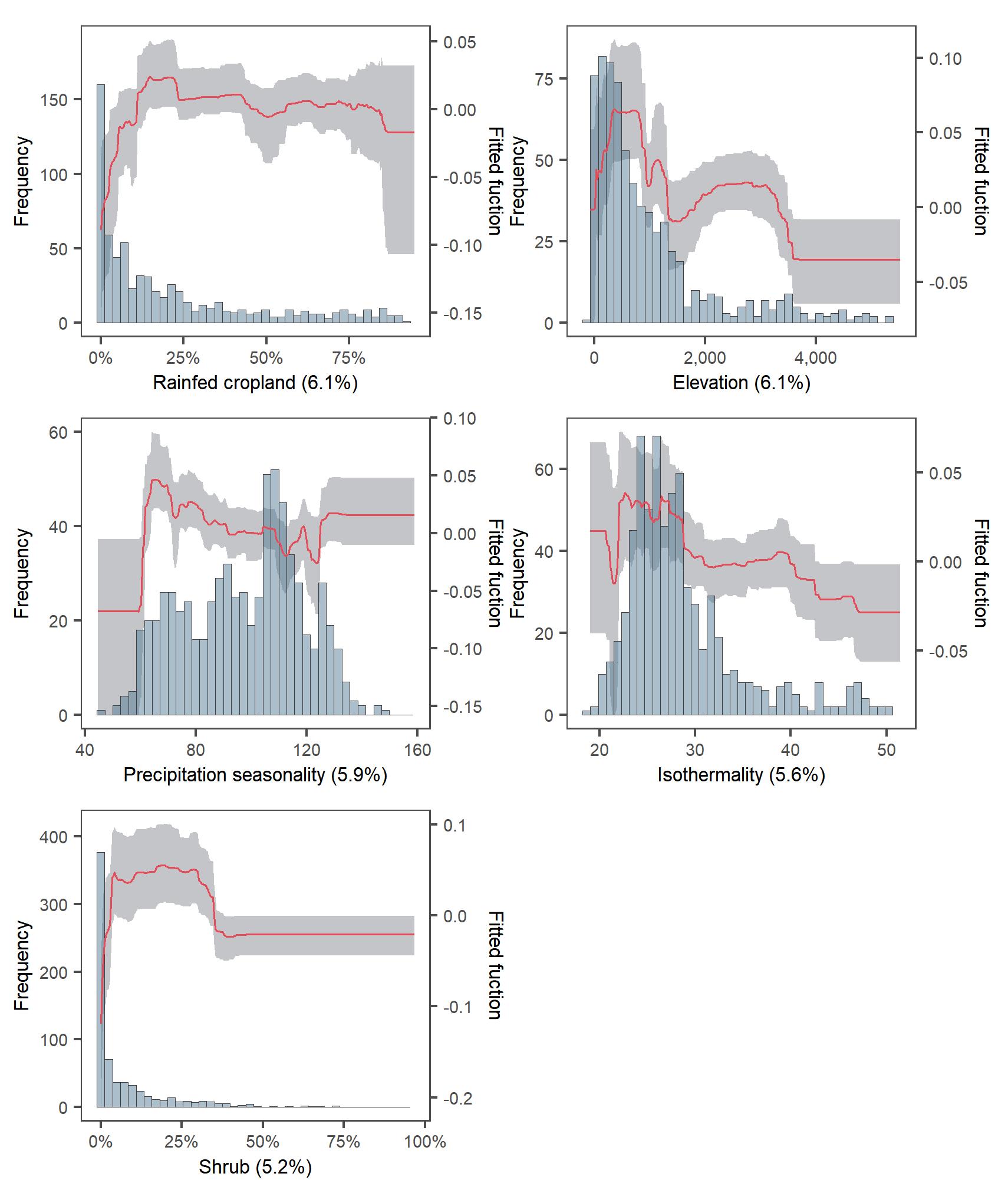


Supplementary Figure 8. The marginal effect of temperature seasonality, isothermality, high coverage grasslands, and population density for *Borrelia burgdorferi* sensu lato risk in the stage 2 model. The red curves and gray bands show the average and range, respectively, of predicted occurrence risk from 100 BRT model using bootstrap method. Frequency distributions of the predictors are shown by the histograms in blue. The percentage values in parentheses show the relative contributions averaged over all 100 BRT models.


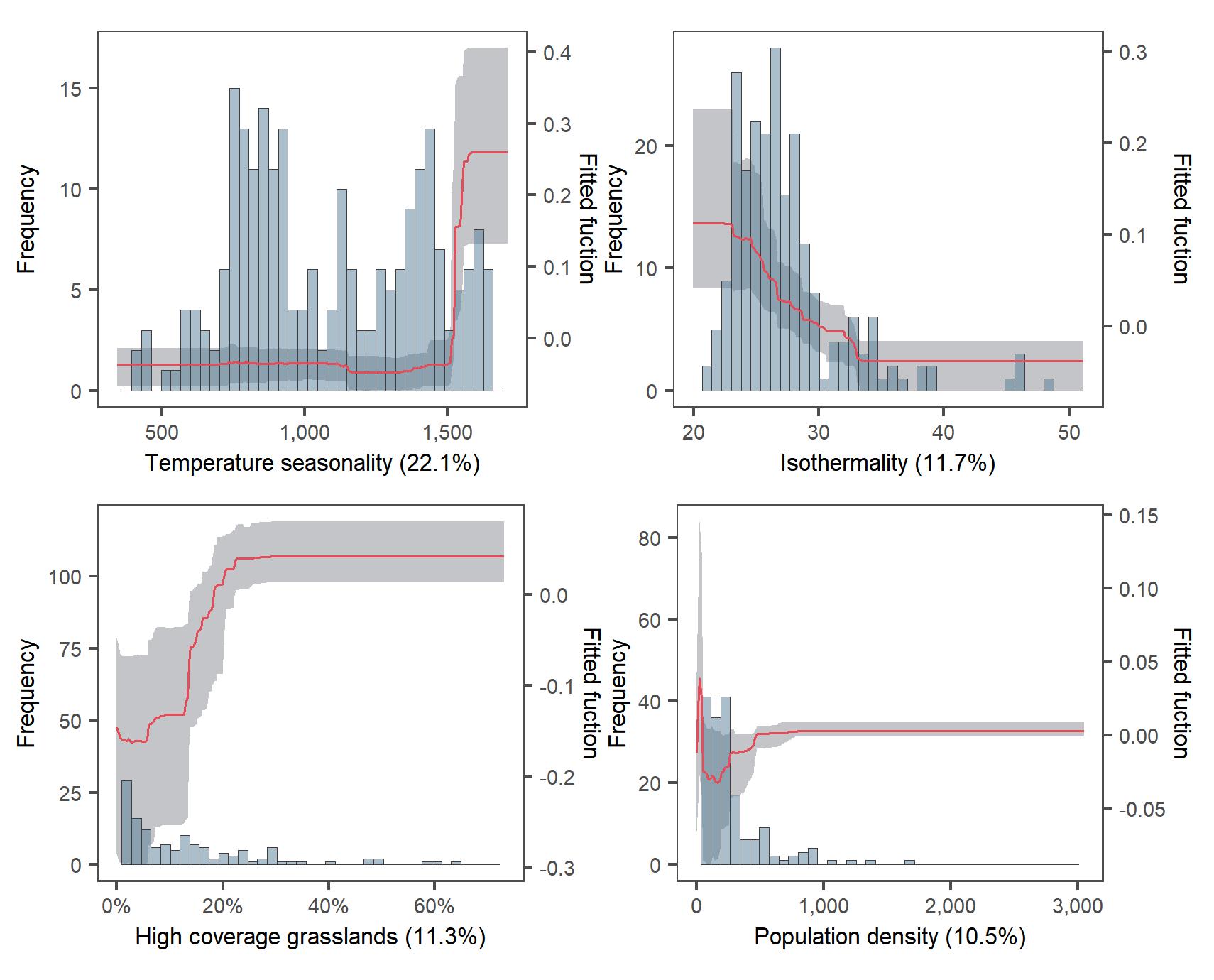


Supplementary Figure 9. The marginal effect of NDVI, shrub, mammalian richness, and moderate coverage grasslands for *Borrelia burgdorferi* sensu lato risk in the stage 2 model. The red curves and gray bands show the average and range, respectively, of predicted occurrence risk from 100 BRT model using bootstrap method. Frequency distributions of the predictors are shown by the histograms in blue. The percentage values in parentheses show the relative contributions averaged over all 100 BRT models.


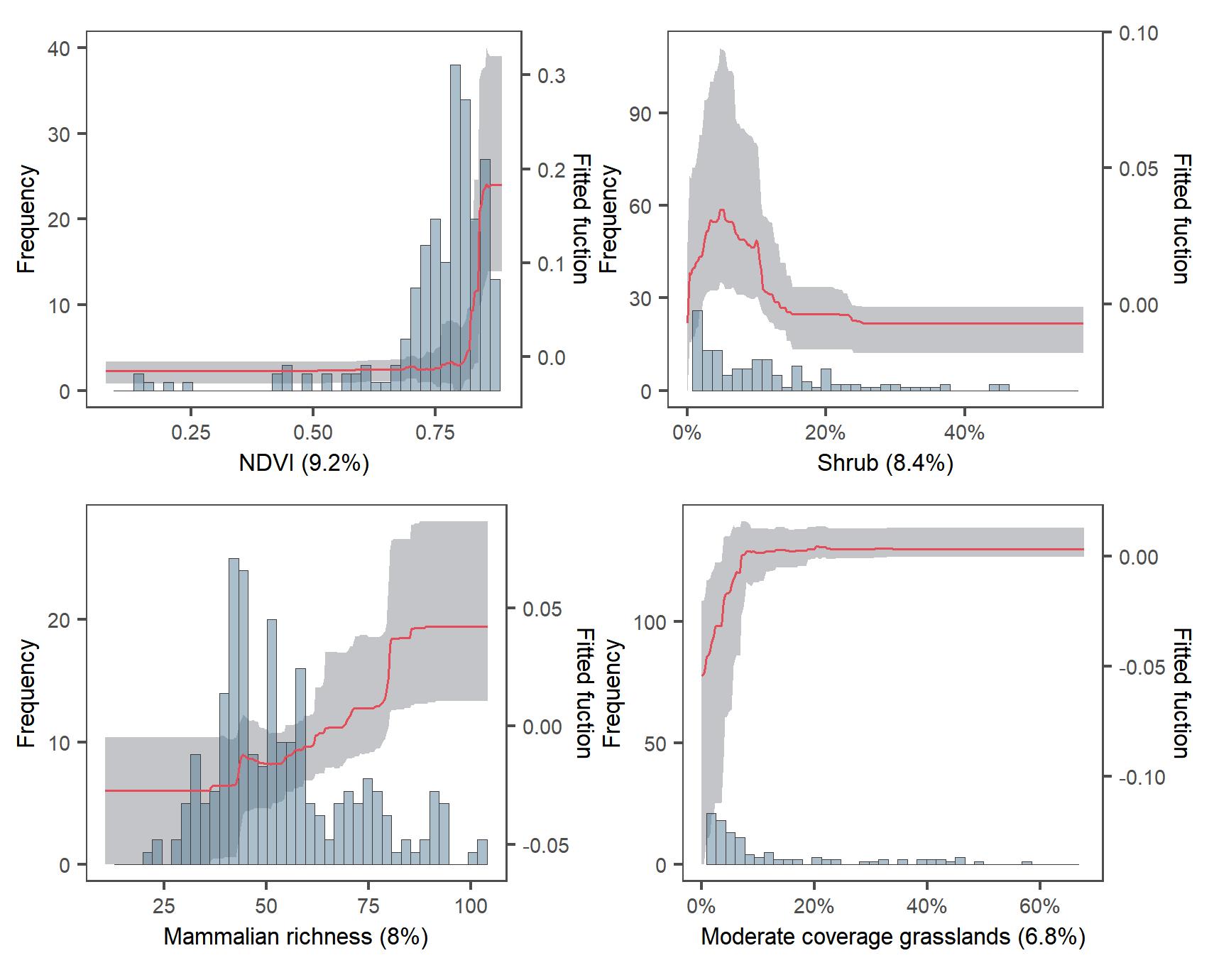


Supplementary Figure 10. The marginal effect of sparse-canopy woodland and other woodland for *Borrelia burgdorferi* sensu lato risk in the stage 2 model. The red curves and gray bands show the average and range, respectively, of predicted occurrence risk from 100 BRT model using bootstrap method. Frequency distributions of the predictors are shown by the histograms in blue. The percentage values in parentheses show the relative contributions averaged over all 100 BRT models.


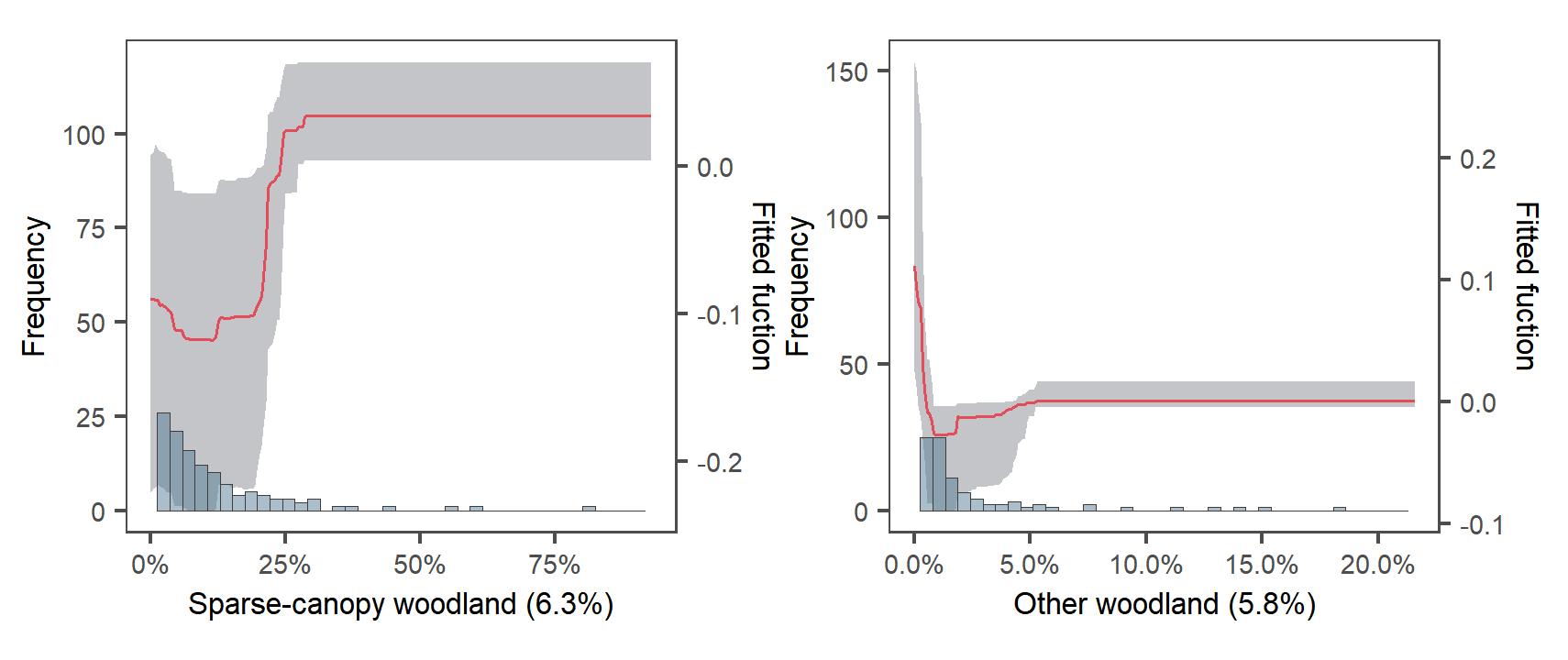


Supplementary Table 6. Relative contribution of environmental variables in the stage 2 model including only human or tick data.

| **Stage 2 model including only human data** | | |  | **Stage 2 model including only tick data** | |  |
| --- | --- | --- | --- | --- | --- | --- |
| **Variable** | **Mean ± sd (%)** | **Effect** |  | **Variable** | **Mean ± sd (%)** | **Effect** |
| Annual mean temperature | 19.37 ± 6.79 | Negative correlation |  | NDVI | 32.95 ± 7.74 | Positive correlation |
| Temperature seasonality | 14.77 ± 6.15 | Positive correlation |  | Moderate coverage grasslands | 17.89 ± 7.88 | Positive correlation |
| High coverage grasslands | 14.31 ± 4.77 | Positive correlation |  | Temperature seasonality | 15.10 ± 6.48 | Positive correlation |
| Isothermality | 12.57 ± 2.29 | Negative correlation |  | Annual mean temperature | 12.36 ± 9.88 | Negative correlation |
| Mammalian richness | 11.89 ± 2.91 | Positive correlation |  | Population density | 8.61 ± 6.00 | Negative correlation |
| Other woodland | 10.19 ± 2.28 | Nonlinear effects |  | Shrub | 6.76 ± 4.36 | Positive correlation |
| Elevation | 9.66 ± 1.95 | Nonlinear effects |  | Mammalian richness | 6.32 ± 5.62 | Negative correlation |
| Total precipitation | 7.24 ± 2.38 | Nonlinear effects |  | - | - | - |

Supplementary Figure 11. Performance evaluation of the BRT model after averaged 100 bootstrapping models. (A) ROC curves and AUC values of Stage 1 model. (B) ROC curves and AUC values of Stage 2 model.


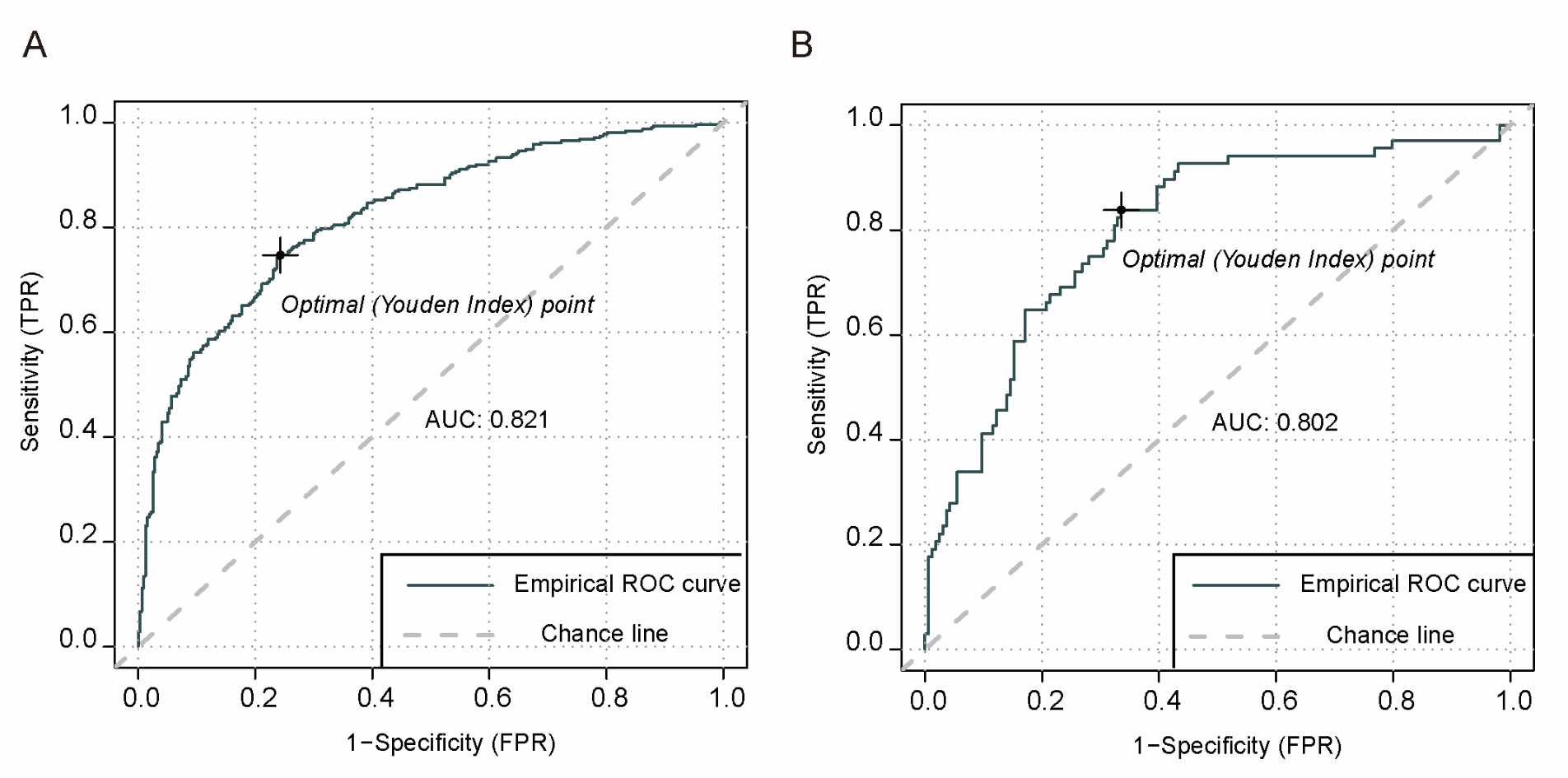


^*^AUC: area under the receiver operating characteristics curve

Supplementary Table 7. Performance evaluation of binary BRT model. Use the best cut-off point when the Youden index is the largest to classify the predicted value and calculate the evaluation indicators of the model.

| Model | Cutoff | Sensitivity | Specificity | Accuracy | F1 score |
| --- | --- | --- | --- | --- | --- |
| Stage 1 | 0.51 | 0.75 (0.70, 0.79) | 0.76 (0.71, 0.80) | 0.75 (0.72, 0.78) | 0.75 (0.16, 0.98) |
| Stage 2 | 0.21 | 0.84 (0.73, 0.91) | 0.66 (0.59, 0.73) | 0.72 (0.65, 0.77) | 0.63 (0.11, 0.96) |

Supplementary References

1. Ai CX, Wen YX, Zhang YG, et al. Clinical manifestations and epidemiological characteristics of Lyme disease in Hailin county, Heilongjiang Province, China. Ann N Y Acad Sci. 1988;539:302-313.
2. Zhang ZF. Investigation of Lyme disease in northeast of China. Chin J Epidemiol. 1989;10(5):261-264.
3. Ai CX, Zhang WF, Zhao JH. Sero-epidemiology of Lyme disease in an endemic area in China. Microbiol Immunol. 1994;38(7):505-509.
4. Li M, Masuzawa T, Takada N, et al. Lyme disease *Borrelia* species in northeastern China resemble those isolated from far eastern Russia and Japan. Appl Environ Microbiol. 1998;64(7):2705-2709.
5. Tian W, Zhang Z, Moldenhauer S, et al. Detection of *Borrelia burgdorferi* from ticks (Acari) in Hebei Province, China. J Med Entomol. 1998;35(2):95-98.
6. Masuzawa T, Takada N, Kudeken M, et al. *Borrelia sinica* sp. nov., a lyme disease-related *Borrelia* species isolated in China. Int J Syst Evol Microbiol. 2001;51(Pt 5):1817-1824.
7. Takada N, Masuzawa T, Ishiguro F, et al. Lyme disease *Borrelia spp.* in ticks and rodents from northwestern China. Appl Environ Microbiol. 2001;67(11):5161-5165.
8. Cao WC, Zhao QM, Zhang PH, et al. Prevalence of Anaplasma phagocytophila and *Borrelia burgdorferi* in *Ixodes persulcatus* ticks from northeastern China. Am J Trop Med Hyg. 2003;68(5):547-550.
9. Sun Y, Liu GP, Yang LW, et al. Multiple infections of tick-borne pathogens in *Ixodes persulcatus* collected from forests in Heilongjiang province. Acta Parasitologica Et Medica Entomologica Sinica. 2007;14.
10. Chu CY, Liu W, Jiang BG, et al. Novel genospecies of *Borrelia burgdorferi* sensu lato from rodents and ticks in southwestern China. J Clin Microbiol. 2008;46(9):3130-3133.
11. Zhan L, Cao WC, Chu CY, et al. Tick-borne agents in rodents, China, 2004-2006. Emerg Infect Dis. 2009;15(12):1904-1908.
12. Zhan L, Chu CY, Zuo SQ, et al. *Anaplasma phagocytophilum* and *Borrelia burgdorferi* in rabbits from southeastern China. Vet Parasitol. 2009;162(3-4):354-356.
13. Zhang F, Gong Z, Zhang J, et al. Prevalence of *Borrelia burgdorferi* sensu lato in rodents from Gansu, northwestern China. BMC Microbiol. 2010;10:157.
14. Chu CY, Jiang BG, He J, et al. Genetic diversity of *Borrelia burgdorferi* sensu lato isolates from Northeastern China. Vector Borne Zoonotic Dis. 2011;11(7):877-882.
15. Chu CY, Jiang BG, Qiu EC, et al. *Borrelia burgdorferi* sensu lato in sheep keds (Melophagus ovinus), Tibet, China. Vet Microbiol. 2011;149(3-4):526-529.
16. Hao Q, Hou X, Geng Z, et al. Distribution of *Borrelia burgdorferi* sensu lato in China. J Clin Microbiol. 2011;49(2):647-650.
17. Zhang L, Cui F, Wang L, et al. Investigation of anaplasmosis in Yiyuan County, Shandong Province, China. Asian Pac J Trop Med. 2011;4(7):568-572.
18. Fu Y, Liu Z, Guan G, et al. Development of real-time polymerase chain reaction for detection of *Borrelia burgdorferi* sensu lato in China. Vector Borne Zoonotic Dis. 2012;12(5):341-345.
19. Wang S, He J, Zhang L. Serological investigation of vector-borne disease in dogs from rural areas of China. Asian Pac J Trop Biomed. 2012;2(2):102-103.
20. Xia Z, Yu D, Mao J, et al. The occurrence of Dirofilaria immitis, *Borrelia burgdorferi*, Ehrlichia canis and Anaplasma phagocytophium in dogs in China. J Helminthol. 2012;86(2):185-189.
21. Geng Z, Hou XX, Hao Q, et al. Optimization of pulse-field gel electrophoresis for *Borrelia burgdorferi* subtyping. Biomed Environ Sci. 2013;26(7):584-591.
22. Hao Q, Geng Z, Hou XX, et al. Seroepidemiological investigation of lyme disease and human granulocytic anaplasmosis among people living in forest areas of eight provinces in China. Biomed Environ Sci. 2013;26(3):185-189.
23. Xuefei D, Qin H, Xiaodi G, et al. Epidemiological and clinical features of three clustered cases co-infected with Lyme disease and rickettsioses. Zoonoses Public Health. 2013;60(7):487-493.
24. Yang J, Guan G, Niu Q, et al. Development and application of a loop-mediated isothermal amplification assay for rapid detection of *Borrelia burgdorferi* s. l. in ticks. Transbound Emerg Dis. 2013;60(3):238-244.
25. Hou X, Xu J, Hao Q, et al. Prevalence of *Borrelia burgdorferi* sensu lato in rodents from Jiangxi, southeastern China region. Int J Clin Exp Med. 2014;7(12):5563-5567.
26. Ni XB, Jia N, Jiang BG, et al. Lyme borreliosis caused by diverse genospecies of *Borrelia burgdorferi* sensu lato in northeastern China. Clin Microbiol Infect. 2014;20(8):808-814.
27. Zhou X, Hou XX, Geng Z, et al. Establishment of multiple locus variable-number tandem repeat analysis assay for genotyping of *Borrelia burgdorferi* sensu lato detected in China. Biomed Environ Sci. 2014;27(9):665-675.
28. Dou X, Lyu Y, Jiang Y, et al. Seroprevalence of Lyme disease and associated risk factors in rural population of Beijing. Int J Clin Exp Med. 2015;8(5):7995-7999.
29. Hou J, Ling F, Chai C, et al. Prevalence of *Borrelia burgdorferi* sensu lato in ticks from eastern China. Am J Trop Med Hyg. 2015;92(2):262-266.
30. Wang YZ, Mu LM, Zhang K, et al. A broad-range survey of ticks from livestock in Northern Xinjiang: changes in tick distribution and the isolation of *Borrelia burgdorferi* sensu stricto. Parasit Vectors. 2015;8:449.
31. Yang J, Liu Z, Guan G, et al. Comprehensive surveillance of the antibody response to *Borrelia burgdorferi* s.l. in small ruminants in China. Ann Agric Environ Med. 2015;22(2):208-211.
32. Yang J, Liu Z, Niu Q, et al. Tick-borne zoonotic pathogens in birds in Guangxi, Southwest China. Parasit Vectors. 2015;8:637.
33. Margos G, Chu CY, Takano A, et al. *Borrelia yangtzensis* sp. nov., a rodent-associated species in Asia, is related to *Borrelia valaisiana*. Int J Syst Evol Microbiol. 2015;65(11):3836-3840.
34. Yu PF, Niu QL, Liu ZJ, et al. Molecular epidemiological surveillance to assess emergence and re-emergence of tick-borne infections in tick samples from China evaluated by nested PCRs. Acta Trop. 2016;158:181-188.
35. Yu P, Liu Z, Niu Q, et al. Molecular evidence of tick-borne pathogens in *Hyalomma anatolicum* ticks infesting cattle in Xinjiang Uygur Autonomous Region, Northwestern China. Exp Appl Acarol. 2017;73(2):269-281.
36. Zhai B, Niu Q, Yang J, et al. Identification and molecular survey of *Borrelia burgdorferi* sensu lato in sika deer (Cervus nippon) from Jilin Province, north-eastern China. Acta Trop. 2017;166:54-57.
37. Zhang J, Liu Q, Wang D, et al. Epidemiological survey of ticks and tick-borne pathogens in pet dogs in south-eastern China. Parasite. 2017;24:35.
38. Wang J, Kelly P, Zhang J, et al. Detection of Dirofilaria immitis antigen and antibodies against *Anaplasma phagocytophilum*, *Borrelia burgdorferi* and *Ehrlichia canis* in dogs from ten provinces of China. Acta Parasitol. 2018;63(2):412-415.
39. Zhai B, Niu Q, Liu Z, et al. First detection and molecular identification of *Borrelia* species in Bactrian camel (Camelus bactrianus) from Northwest China. Infect Genet Evol. 2018;64:149-155.
40. Jiang Y, Hou X, Zhang L, et al. Case report: A patient coinfected by *Borrelia burgdorferi* sensu lato and spotted fever group Rickettsiae in Urumqi, China. Medicine (Baltimore). 2019;98(46):e17977.
41. Li X, Li P, Zhang T, et al. A Serological Survey of *Borrelia burgdorferi* Infection in Sheep in Northeast China Regions Through Outer Surface Protein C-Based Enzyme-Linked Immunosorbent Assay. Vector Borne Zoonotic Dis. 2019;19(1):16-21.
42. Liu HB, Wei R, Ni XB, et al. The prevalence and clinical characteristics of tick-borne diseases at One Sentinel Hospital in Northeastern China. Parasitology. 2019;146(2):161-167.
43. Wang Y, Li S, Wang Z, et al. Prevalence and Identification of *Borrelia burgdorferi* Sensu Lato Genospecies in Ticks from Northeastern China. Vector Borne Zoonotic Dis. 2019;19(5):309-315.
44. Ai CX, Wen YX, Zhang YG, et al. Epidemiological investigation of Lyme disease in the forest area of Hailin County, Heilongjiang Province. Chin J Publ Heal. 1987;6(02):82-85+128.
45. Cao YX, Zhang XT, Ma J, et al. Isolation of Lyme disease Pathogen from *Ixodes persulcatus* collected in Xinjiang. Chin J Zoono. 1988(04):10-11+12.
46. Zhang QD. Our military researchers found that the Nalati area of Xinjiang is a natural foci of Lyme disease. People Mil Surg. 1988(12):80.
47. Li SQ, Wang FC, Zhang QD, et al. A clinical study of Lyme disease in the western forest area of Tianshan Mountains. Chinese Journal of Endemiology. 1989(06):3.
48. Cao YX, Zhang XT, Lian XY, et al. Natural foci of Lyme disease in Tianshan forest area in Xinjiang. Chin J Publ Heal. 1990(04):199.
49. Li SQ, Wang FC, Zhang QD, et al. Investigation on the natural foci of Lyme disease in the western forest area of Tianshan Mountains, Xinjiang. People Mil Surg. 1990(01):47-48.
50. Li SQ, Wang FC, Zhang QD, et al. Investigation report on the natural foci of Lyme disease in the western forest area of Tianshan Mountains. Medical Journal of National Defending Forces in Northwest China. 1990(08):13-15.
51. Pan L, Yu DS, Lin JR, et al. Lyme disease found in Fujian Province. Chin J Zoono. 1990(05):63.
52. Wang RK, Sun HY. Investigation report on Lyme disease in Langxi County. Anhui Medical Journal. 1990(06):40-41.
53. Zhang QD, Ye ZF, Zhu YF, et al. Isolation of Lyme disease pathogens from *Ixodes panaxa* collected from Inner Mongolia. Chin J Zoono. 1990(05):5-6+66.
54. Zhang QD, Zhang PH. Investigation on the natural foci of Lyme disease in a certain area of Xinjiang. J Prev Med Chin Peopl Liber Army. 1990(04):364-367.
55. Zhou QY, Zhao MY, Huang ZY, et al. The first Lyme disease antibody was detected in the sera of the population in Sichuan. Journal of Preventive Medicine Information. 1990(03):134.
56. Dong Y. Investigation report on Lyme disease in Weihe forest area of Heilongjiang Province. Chin J Zoono. 1991(04):36-37.
57. Lai CL, Fang JS, Zeng J, et al. A preliminary report on the seroepidemiological investigation of Lyme disease among forest farm workers. Guangxi Medical Journal. 1991(5):343-344.
58. Li YL, Hao JG, Zhang ZF, et al. Isolation of Lyme disease spirochetes from *Haemaphysalis dioica* in forest areas in eastern Sichuan. Chin J Vector Biol Control. 1991(06):386-387.
59. Li YL, Hao JG, Zhang ZF, et al. Investigation on Lyme disease in Nanchuan County, Sichuan Province. Chin J Vector Biol Control. 1991(01):54-56.
60. Ma HB, Yang WY, Yang XD, et al. Seroepidemiological investigation report of Lyme disease in Lijiang and Menglian in Yunnan. Medicine and Pharmacy of Yunnan. 1991(06):384-385.
61. Weng CR, Zhang ZF, Yang XZ, et al. Epidemiological study of Lyme disease in Guizhou (Preliminary investigation of Lyme disease infection in Dajia Forest Farm and its forest area in Liping County). Guizhou Medical Journal. 1991(04):208.
62. Xie XC, Liu HJ, Yang XK, et al. Isolation and identification of *Borrelia burgdorferi* in mountainous areas of Bole, Shawan, Fukang, Xinjiang. Endemic Diseases Bulletin (China). 1991(04):8-11.
63. Zhou QY, Zhao MY, Huang ZY, et al. Seroepidemiological survey of Lyme disease among healthy people in Sichuan Province. Journal of Preventive Medicine Information. 1991(01):46-47+38.
64. Fan LM. A report of 2 cases of Lyme disease with meningitis and meningoencephalitis. Chin J Nervous Mental Dis. 1992(05):289.
65. Liu ZJ, Wang DH, Zhang XP, et al. Investigation and study of *Borrelia burgdorferi* in the northern foothills of Qilian Mountains, Gansu. Chinese Journal of Veterinary Science and Technology. 1992(11):4.
66. Lu JP, Zhu HP, Yang XF, et al. Investigation of Lyme disease in forest area of Hulunbuir League. Inner Mongolia Medical Journal. 1992(03):30-31.
67. Ma HB, Wan KL, Zhang ZF. Comparison of three test methods for detecting Lyme disease antibodies. Chin J Zoono. 1992(01):32-34.
68. Ren GS, Yan DC, Ran XL, et al. Investigation on the seasonal fluctuation of *Ixodes scoparium* in the natural foci of Lyme disease in the Great Khingan Mountains, Inner Mongolia. Journal of Medical Pest Control. 1992(02):107-109.
69. Tu SQ, Li YS. One case report of Lyme disease. Beijing Medical Journal. 1992(06):352.
70. Wang DH, Liu ZJ, Su DS, et al. Preliminary Investigation on the Seroepidemiology of Lyme disease in Yak. Chinese Journal of Veterinary Science and Technology. 1992(06):2.
71. Wang JZ, Ning LY. Seroepidemiological survey of Lyme disease in Qingyang County. Chinese Journal of Rural Medicine. 1992(07):23.
72. Xiao GL, Yan XL, Liu GZ, et al. Seroepidemiological investigation report of Lyme disease in Jiangsu Province. Chin J Publ Heal. 1992(11):486.
73. Zhang DC, Hong Y, Zhang ZQ, et al. *Borrelia burgdorferi* isolated from ticks and rats in Liaoning. Chin J Vector Biol Control. 1992(01):19.
74. Cai ZL, Lu ZX, Hu LM, et al. Investigation of Human serum Lyme disease antibodies in Northeast China. Chin J Publ Heal. 1993(02):98.
75. Chen SA, Feng FB, Yang YL, et al. Lyme disease among professional fruit workers in the northwestern suburbs of Beijing. Beijing Medical Journal. 1993(03):137-139.
76. Chen ZG, Liu WP, Wu TW, et al. A case report of typical advanced Lyme disease. Chin J Zoono. 1993(01):16.
77. Huang YP, Zheng HN, Cai SG, et al. Discovery and investigation of Lyme disease in southern Fujian. Chin J Zoono. 1993(01):46-47.
78. Liu ZJ, Kang XM, Su DS, et al. Investigation on the natural foci of Lyme disease in Huajian area on the northern foot of Qilian Mountains. Chinese Journal of Veterinary Science and Technology. 1993(11):3.
79. Shen Z, Wang J, Zhang FQ, et al. Investigation of Lyme disease in the mountainous area of southwest Beijing. Chin J Publ Heal. 1993(S1):11-13.
80. Wang ZM, Qiu Q, Yu ZR, et al. Isolation of Lyme disease spirochetes from hares. Journal of Clinical Research. 1993(02):71.
81. Wang ZM, Wan KL, Qiu Q, et al. Investigation and research on Lyme disease in Huaihua District, Hunan Province. Chin J Vector Biol Control. 1993(03):3.
82. Xie XC, Liu HJ, Ye DK, et al. The report of the Lyme disease foci found in the Tianshan Mountains in Urumqi County. Endemic Diseases Bulletin (China). 1993(04):110-124.
83. Xu GD, Su DS. Study on the parasitic ticks in early spring cattle and sheep, Vector of Lyme disease. Journal of Lanzhou University. 1993(04):302-297.
84. Yu JS. The first discovery and epidemiological study of Lyme disease in Xinjiang. Tacheng Technology. 1993;00(01):28-29.
85. Yu JS, An P. Lyme disease found in the arid zone of the desert. Chin J Zoono. 1993(01):8.
86. Zhang QD, Zhu YF, Zhang PH, et al. Preliminary investigation on the geographical distribution of Lyme disease in my country. J Prev Med Chin Peopl Liber Army. 1993(03):206-208.
87. Zhang X, Zheng XY, Zhao X. Investigation report on Lyme disease in Hebei Province. J Prev Med Chin Peopl Liber Army. 1993(05):365-366.
88. Zhang ZK, Tian WC, Guo YX, et al. Seroepidemiological survey of Lyme disease in some populations in Hebei Province. Chin J Publ Heal. 1993(05):240.
89. Zhu JH, Wang SY, Cai HT, et al. Investigation of Lyme disease vectors in the Great Khingan Mountains of Inner Mongolia. Chin J Publ Heal. 1993(03):109.
90. Cai DH, He YX, Wang JS, et al. Investigation on the natural foci of Lyme disease in Qingyuan County. Chin J Vector Biol Control. 1994(05):2.
91. Chen WS, Feng BQ, Dong ZM, et al. Serological investigation of Lyme disease in three counties of northern Guangdong Province. Guangdong Journal of Health and Epidemic Prevention. 1994(04):2.
92. Chen YK. Investigation on serum antibodies of goat Lyme disease. Chinese Journal of Veterinary Science and Technology. 1994(02):2.
93. Feng FB, Zhang WF, Zhou GP, et al. Discovery and clinical investigation of Lyme disease in Beijing area. Chin J Epidemiol. 1994(01):4.
94. Guan SZ, Yin GC, Han SQ, et al. Detection of anti-*Borrelia burgdorferi* antibodies in human sero by VIDAS system. Chin J Epidemiol. 1994(06):4.
95. Guo WS, Li LC, Xia ZG, et al. Seroepidemiological survey of Lyme disease in Xin'an County. Henan Journal of Preventive Medicine. 1994(03):2.
96. Li H. A case of Lyme disease with skin ulcer as the first manifestation. Clinical Medicine of China. 1994(03):2.
97. Li LC, Guo WS, Zhang YP, et al. Investigation of Lyme disease infection in southwest Luoshan and Huaibing counties of Henan. Henan Med Res. 1994(02):3.
98. Liu ZJ. Lyme disease was also found in Gansu Province and its introduction. Lanzhou Science and Technology Information. 1994(02):146.
99. Liu ZJ. Two cases report of Lyme disease. Medical Journal of National Defending Forces in Northwest China. 1994(02):10-12.
100. Liu ZJ, Shi SZ, Wang DH, et al. Seroepidemiological investigation of Lyme disease among people in Diebu forest area. Chin J Publ Heal. 1994(02):1.
101. Luo GL, Pan L, Yu SS. Report of 4 cases of Lyme disease in Wuyishan forest area. Chin J Zoono. 1994(02):1.
102. Ma HB, Zhang Q, Yang WY, et al. Detection report of Lyme disease antibody in fever patients in Yingjiang County. Medical Journal of Dali College. 1994(03):3.
103. Quan DH, Ji JL, Li Q, et al. Two cases of misdiagnosis of Lyme disease. Clinical Misdiagnosis & Mistherapy. 1994(06):1.
104. Shen JJ, Zhang JS, Jiang WM, et al. Epidemiological status of Lyme disease in the northwestern margin of Fujian. Chin J Publ Heal. 1994(03):1.
105. Song XL, Zhang JT, Lv GP, et al. The Investigation of Lyme diseases in forest region in San Cha Zi Hunjiang City. Dis Surveill. 1994(04):2.
106. Wang GQ, Mo YM, Chen XY, et al. Lyme disease found in northeastern Hubei Province. Medical Journal of Wuhan University. 1994(04):4.
107. Wu GJ, Chen GP. A case report of Lyme disease seropositive in Wenzhou. Zhejiang Journal of Preventive Medicine. 1994(05):25.
108. Ye JC, Xu LC, Gao YP, et al. Investigation of Lyme disease in Men Tou Gou District Beijing. Dis Surveill. 1994(07):3.
109. Zhang XP, Wang DH, Su DS, et al. Investigation of Lyme disease in Huajian area. Chinese Journal of Veterinary Science and Technology. 1994(10):2.
110. Lai CL, Fang JS, Liu J, et al. Serological investigation of Lyme disease in three psychiatric hospitals. Journal of Applied Preventive Medicine. 1995(03):1.
111. Li ZM. Progressive pulmonary fibrosis caused by Lyme disease: a case report. Chin J Infect. 1995(1):1.
112. Liu GR, Ma XL, Qu YZ, et al. Seroepidemiological survey of Lyme disease in forest areas of three counties in Qinghai Province. Chin J Publ Heal. 1995(04):1.
113. Quan YW, Yan RJ, Ji WX, et al. Seroepidemiological survey of Lyme disease among population in Huadian City, Jilin Province. Chin J Vector Biol Control. 1995(06):2.
114. Tian H, Han Y, Jiang H. Seroepidemiological investigation of antibody against *Borrelia burgdorferi* in Guiyang port. Chinese Journal of Frontier health and Quarantine. 1995(03):136-137+190.
115. Tian WC, Li MQ, Zhang ZK, et al. *Borrelia burgdorferi* First Found in *Haemaphysalis japonicum* by PCR. Chin J Zoono. 1995(03):4.
116. Wang SG, Qu GH, Wang XC, et al. The clinical analysis of 26 patients double-infected forest encephalitis and Lyme disease. Chin J Vector Biol Control. 1995(04):3.
117. Yang M, Jiao H, Wang HY. A case report of Lyme disease with anxiety disorder as the first symptom. Chin J Vector Biol Control. 1995(06):1.
118. Yang XM, Yong XD, Zhang Y. Four Cases of Lyme diseases in one family. Chin J Zoono. 1995(03):1.
119. Yang ZQ, Liu YX, Sun HL, et al. Preliminary investigation on sera antibodies of 777 soldiers with Lyme disease. J Prev Med Chin Peopl Liber Army. 1995(06):1.
120. Zhang ZK, Tian WC, Guo YX, et al. Investigation of Lyme disease in Hebei Province. Chin J Publ Heal. 1995(06):2.
121. Jia WC, Wan KL, Zhang SQ, et al. Spirochetes isolated from the blood of two patients with Lyme disease. Chin J Vector Biol Control. 1996(02):2.
122. Li YT, Cao YL, Shi JM, et al. Seroepidemiological survey of Lyme disease in Shanghai. Chin J Vector Biol Control. 1996(01):2.
123. Liang JG, Zhang ZF. Analysis of rRNA gene restriction fragment length polymorphism of *B. burgdorferi* sensu lato isolated in China. Chin J Microbiol Immunol. 1996(05):4.
124. Lu JP, Yang XF, Zhao G, et al. Investigation of Lyme disease in desert area of Alxa League in Inner Mongolia. Chin J Vector Biol Control. 1996(03):1.
125. Ma HB, Zhang Q. A case of Lyme disease found in Chongjianghe forest area in Lijiang. Medicine and Pharmacy of Yunnan. 1996(01):1.
126. Pan L, Chen ZG, Huang YP, et al. Clinical and epidemiological analysis on Lyme disease in Fujian, China. Strait J Prev Med. 1996(03):3.
127. Pan L, Yu DS, Chen ZG, et al. Study on the host animal and transmission vector of Lyme disease in Fujian province. Strait J Prev Med. 1996(02):2.
128. Sun CX, Tian H, Li QJ, et al. Application of polymerase chain technology in the study of the transmission route of Lyme disease ticks. Chinese Journal of Veterinary Science and Technology. 1996(11):2.
129. Wang FJ. Report of 6 cases of Lyme disease caused by tick bite. Journal of Medical Pest Control. 1996(04):44-45.
130. Wang SG, Qu GH, Wei B, et al. The clinical analysis of 26 patients double-infected forest encephalitis and Lyme disease. Chin J Infect. 1996(02):2.
131. Xia XH, Mei GS. A case of Lyme disease in children. Chinese Journal of Pediatrics. 1996(02):1.
132. Xie XC, Li QP. SDS-PAGE analysis of two *Borrelia burgdorferi*-XjI3 and XjI12 strains in Xinjiang, China. Endemic Diseases Bulletin (China). 1996;11(02):27-29.
133. Xie XC, Liu HJ, Ye DK, et al. The geographical distribution and epidemiological survey of Lyme disease in Xinjiang. Endemic Diseases Bulletin (China). 1996(03):4.
134. Yan DC. Serological detection and analysis of mixed infection of forest encephalitis and Lyme disease in Great Khingan forest area. Dis Surveill. 1996(05):2.
135. Yang XF, Lu JP, Zhang JL, et al. Investigation and analysis of Lyme disease infection in some populations in Baotou and Hohhot. Chin J Vector Biol Control. 1996(01):1.
136. Yang XF, Lu JP, Zhao G, et al. Analysis of 39 cases of Lyme disease in the forest and pastoral area of Inner Mongolia. Inner Mongolia Medical Journal. 1996(05):1.
137. Zhang DC, Zhang ZQ, Sun GJ, et al. Investigation on Lyme disease in mountainous areas in eastern Liaoning. Chin J Epidemiol. 1996(05):1.
138. Zhang DR, Hang HG, Liu H, et al. A survey on the seroepidemiology of Lyme disease in Anhui province. Chin J Epidemiol. 1996(01):24.
139. Zhang QQ, Niu JQ, Yang QF, et al. Investigation Report on Lyme disease in forest area of Changbai Mountain of Jilin Province. Chin J Epidemiol. 1996(02):116.
140. Bai XJ. Seroepidemiological survey of Lyme disease in Tianzhu County, Gansu Province. J Prev Med Chin Peopl Liber Army. 1997(06):1.
141. Cao CJ, Li H, Wang Q, et al. Investigation on serology and symptomology of Lyme disease in the Great Khingan Mountains of Inner Mongolia. Chin J Zoono. 1997(04):76-77.
142. Deng AQ, Jiang HH, Huang WQ, et al. Analysis of clinical features of Lyme disease. Anhui Medical Journal. 1997(01):15-16.
143. Gong ZW, Shi SZ, Luo YQ, et al. Preliminary investigation of Lyme disease in a certain department and resident in Jiuquan. Lanzhou Science and Technology Information. 1997(04):10-11.
144. Huang P. A summary of natural focus diseases and medical insects in Tibet. Science of Travel Medicine. 1997(04):156-157+161.
145. Li H. Clinical manifestations of 170 cases of Lyme disease. Inner Mongolia Medical Journal. 1997(03):161-162.
146. Li ZG, Ma FH, Wu MY, et al. Study on Lyme disease in the liupanshan of Ningxia. Chin J Vector Biol Control. 1997(06):441-443.
147. Liu QW, Zhang LX, Li JH, et al. Seroepidemiological survey of Lyme disease in Chenzhou area. Dis Surveill. 1997(05):2.
148. Liu ZJ, Zhang XP, Shi SZ, et al. Studies on clinical epidemiology of 104 cases of Lyme disease. J Prev Med Chin Peopl Liber Army. 1997(05):4.
149. Ma HB, Zhang Q. Investigation on serum Lyme disease antibodies in 12 species of rats in Yunnan Province. Chin J Vector Biol Control. 1997(02):84.
150. Tian H, Li QJ, Yan XJ, et al. Detection of *Borrelia burgdorferi* by polymerase chain reaction. Chinese Journal of Veterinary Science and Technology. 1997(12):2.
151. Tian MF, Han Y, Long WB, et al. Preliminary investigation of Lyme disease along the newly built Hengnan Line. Railway Medical Journal. 1997(01):1.
152. Zhang DR, Hang HG, Li Q. Preliminary investigation of Lyme disease infection among the population in Linzhi, Tibet. Chin J Zoono. 1997(02):70-16.
153. Zhang XP, Liu ZJ, Shi SZ, et al. Serological survey of Lyme disease among populations in Diebu area at the northern foot of Minshan Mountain in Gansu Province. Chin J Zoono. 1997(04):72-73.
154. Zhang ZF, Wan KL, Zhang JS, et al. Studies on epidemiology and etiology of Lyme disease in China. Chin J Epidemiol. 1997(01):4.
155. Zhao YY, Sun SY, Liu WK, et al. Seroepidemiological survey of Lyme disease in Dalian. Chin J Zoono. 1997(06):69-71.
156. Bi S, Wang GX, Zhao L, et al. Lyme disease and neurological manifestations (report of 4 cases). Stroke Nervo Dis. 1998(01):3.
157. Chen WS, Wan KL, Hao RF, et al. Lyme disease spirochetes isolated for the first time in Guangdong. Chin J Vector Biol Control. 1998(04):2.
158. Hua MT, Lin T, Liu CL, et al. Serological survey of Lyme disease in humans and animals in Altay, Xinjiang. Medical Journal of National Defending Forces in Northwest China. 1998(04):3.
159. Hua MT, Lin T, Liu CL, et al. Studies on the seroepidemiology of Lyme disease of human and animals in ArLartai area of Xinjiang Province. Chin J Vector Biol Control. 1998(03):2.
160. Tian H, Li QJ, Yang WH, et al. *Borrelia burgdorferi* was isolated in Guangdong. Chinese Journal of Veterinary Science and Technology. 1998(03):2.
161. Wan KL, Zhang ZF, Dou GL, et al. *Ixodes persulcatus* plays a leading role in the transmission of *Borrelia burgdorferi* sensu lato to human in Northern region of China. Practical Preventive Medicine. 1998(06):4.
162. Wan KL, Zhang ZF, Zhang JS, et al. Preliminary investigation on Lyme disease in animals in 20 provinces, cities and autonomous regions of China. Chin J Vector Biol Control. 1998(05):5.
163. Wang LJ, Chen ZL. Report of 2 cases of Lyme disease in Shandong Province. Chin J Vector Biol Control. 1998(06):1.
164. Wei AM, Wu YM, Liu XX, et al. Seroepidemiological survey of three tick-borne diseases in Dongning and Suiyang. Shen Budui Yiyao. 1998;11(06):523-524.
165. Zhang DR, Lin T, Li Q, et al. Investigation on the Seroepidemiology of Lyme disease in Anhui Province. Chin J Vector Biol Control. 1998(04):3.
166. Zhang MJ, Wan KL, Hou XX, et al. Analysis of detection results of anti-*Borrelia burgdorferi* antibody in 268 suspected Lyme disease patients in Beijing. Dis Surveill. 1998(03):3.
167. Hua MT, Jin ZQ, Lin T, et al. Investigation of Lyme disease focus of natural infection in the border areas near Russia and Kazakhstan. J Prev Med Chin Peopl Liber Army. 1999(06):402-405.
168. Hua MT, Jin ZQ, Wang ZT, et al. A preliminary study of *Borrelia burgdorferi* carried in Mole Voles. Journal of Medical Pest Control. 1999(10):525-526.
169. Liu ZH, Tan QD, Dong GR. Report of 3 cases of Lyme disease. Chin J Vector Biol Control. 1999(03):1.
170. Long J, Lin T, Li WB, et al. Investigation on human and animal Lyme disease in Shanggao County of Jiangxi Province. Chin J Vector Biol Control. 1999(01):3.
171. Shi SZ. A case report of hearing loss caused by Lyme disease. Gansu Science and Technology. 1999(02):1.
172. Tian MF, Han Y, Long WB, et al. Serological survey of Lyme disease in railway construction population. Railway Medical Journal. 1999(05):298-299.
173. Tian MF, Han Y, Zhu JL, et al. Serological survey of Lyme disease in outpatients and inpatients of Leiyang Railway Hospital in Hunan Province. Practical Preventive Medicine. 1999(06):445.
174. Tian Z, Dong JJ, Sun HS, et al. Seroepidemiological investigation of Lyme disease in oil workers of Henan Oil Prospecting Bureou in Xinjiang. Chin J Vector Biol Control. 1999(05):361-363.
175. Wan KL, Zhang ZF, Wang HY, et al. Preliminary investigation on reservoir hosts of *Borrelia burgdorferi* in China. Journal of Hygiene Research. 1999(01):3.
176. Wang CQ, Pan HM, Li ZJ, et al. Investigation on Lyme disease in Xingshan County, Western Hubei. Chin J Vector Biol Control. 1999(02):2.
177. Wang FH, Ding JF, Jiao H. Analysis of nervous system manifestations in 32 Cases of Lyme disease. Chin J Vector Biol Control. 1999(04):1.
178. Yang XF, Cheng KL, Lu JP, et al. Geographic epidemiological investigation and etiological study of Lyme disease in Inner Mongolia. Chin J Zoono. 1999(02):2.
179. Zhang LB, Cheng JF, Wan KL, et al. Seroepidemiological survey of Lyme disease in Hubei Province, China. Chin J Vector Biol Control. 1999(03):2.
180. Chen WS, Hao RF, Luo HM, et al. New confirmed focus of Lyme disease in Guangdong province. Chin J Zoono. 2000(02):105-166.
181. Guo Y, Wan KL, Xu SE, et al. The discovery and study on the natural focus of Lyme disease in eastern Guandong Province. Chin J Zoono. 2000(02):42-45.
182. Hua MT, Jin ZQ, Gong ZW, et al. Investigation of common Ticks carrying *Borrelia burgdorferi* in Tiereketilin Pasturing Area of Haba River. Chin J Zoono. 2000(01):110-105.
183. Hua MT, Jin ZQ, He C, et al. Investigation of *Borrelia burgdorferi* carried by rat in forest and pastoral. Chin J Zoono. 2000(03):110.
184. Lan JQ, Tang AJ, He SY, et al. Investigation on serum antibodies of canine Lyme disease in Yunnan. Yunnan Journal Of Animal Husbandry & Veterinary Medicine. 2000(04):20.
185. Li XM, Zhang DR, Chen CW, et al. Seroepidemiological survey of Lyme disease in Hongta District, Yuxi City. Chin J Vector Biol Control. 2000(01):15.
186. Liang H, Yang DL, Hong PK, et al. Report of 2 cases of Lyme disease found for the first time in Jinjiang City. Fujian Medical Journal. 2000(S1):330-331.
187. Liu ZJ, Hua MT, Shi SZ, et al. Studies on relation between Lyme disease infection in human, livestock, and rodents. Journal of Medical Pest Control. 2000(06):298-301.
188. Shi CX, Zhang ZF, Wan KL. Analyzing clinical samples of Lyme disease with 5s-23s rRNA gene spacer RFLP analysis. Chin J Zoono. 2000(02):21-23.
189. Wang LJ, Wan KL, Liu SL, et al. The first discovery of endemic Lyme disease in Shandong province. Chin J Epidemiol. 2000(04):3.
190. Wang R. Report of 6 cases of Lyme disease. Ningxia Medical Journal. 2000(04):243-244.
191. Yan XL. The investigation on serum epidemiology of Lyme disease in Jiangsu Province. Chin J Publ Heal. 2000(01):1.
192. Zhang YC, Li LJ, Lei SJ, et al. Investigation of Lyme disease in Yuxi area of Yunnan. Chin J Zoono. 2000(06):107-108.
193. Chen ZX, Wu LJ, Zheng LF, et al. A survey on serum antibody of Lyme disease in Guangxi goats. Guangxi Journal Of Animal Husbandry & Veterinary Medicine. 2001(04):8-9.
194. Hua MT, Jin ZQ, Liu CL, et al. A report of 15 cases of Lyme disease in the Habahe and Burqin forest pastoral areas of Altai Mountains. Medical Journal of National Defending Forces in Northwest China. 2001(01):74.
195. Huang ZY, Hou XX, Wan KL. An Investigation of Lyme disease in Pingjiang County, Hunan Province, China. Chin J Vector Biol Control. 2001(04):293-294.
196. Jiang SY, Yi H, Gong TX, et al. Seroepidemiological investigation and analysis of Lyme disease in Tongde County, Qinghai Province. Qinghai Medical Journal. 2001(12):51-52.
197. Liu Y, Chen JY, Song CY, et al. The first report on the investigation of Lyme disease in Ji County, Tianjin. Journal of Medical Pest Control. 2001(09):485-487.
198. Qu ZF, Gan LH, Li Y, et al. Clinical analysis of 34 cases of Lyme disease. Journal of Apoplexy and Nervous Diseases. 2001(01):1.
199. Tan YH, Liu Y, He YX, et al. A case of Lyme disease with severe meningoencephalitis. Chinese Journal of Clinical Neurosciences. 2001(04):337.
200. Wang DH. Sero-epidemiological investigation of Lyme disease. Journal of Medical Pest Control. 2001(06):302-304.
201. Wang P, Jin ZH, Han YX, et al. A case report of Lyme disease misdiagnosed as tick sting. Jilin Medical Journal. 2001(01):30.
202. Yang JC. Seroepidemiological survey of Lyme disease in Xuzhou. Chin J Publ Heal. 2001(04):1.
203. Yang XJ, Wang CS, Gao LY, et al. Investigation of Lyme disease in Changbai Mountain. Chin J Zoono. 2001(02):104-161.
204. Zhang ZD, Chen YJ, Mei JH, et al. Seroepidemiological study of Lyme disease in the population of southern Zhejiang. Chin J Publ Heal. 2001(11):2.
205. An JY, Yao GL, Li QM, et al. Experimental Observation of Lyme disease Vector Tick and Tick *Borrelia burgdorferi* in Xishan Area of Beijing. The Fifth National Conference on Biomedical Stereology, the Eighth Military Pathology Academic Conference, and the Fourth Military Quantitative Pathology Academic Conference; Lanzhou, China; 2002.
206. Ding XN. Lyme neuroborreliosis: report of a case. Journal of Practical Medical Techniques. 2002(11):882-883.
207. Fan LH, Wang LS, Zou HY, et al. Seroepidemiological survey of Lyme disease in forest farms in Heihe area. Chin J Vector Biol Control. 2002(05):347.
208. Hua MT, Liu CL, Wang K, et al. Detection and analysis of serum Lyme disease antibodies of officers and soldiers and the masses stationed in the forest region of Altai mountains. Medical Journal of National Defending Forces in Northwest China. 2002(01):66-67.
209. Jia YP, He JH, Gao F, et al. Analysis of Lyme disease infection in a certain border control department in Inner Mongolia. J Prev Med Chin Peopl Liber Army. 2002(02):123-124.
210. Li JG, Xiao YM, Yang GQ, et al. 1 case report of Lyme disease. The Chinese Journal of Dermatovenereology. 2002(02):1.
211. Li JM, Cao WC, Zhang XT, et al. Exploratory study on natural focus and its causative agent of genospecies of Lyme disease by polymerase chain reaction in the forest areas of Beijing. Chin J Epidemiol. 2002(03):4.
212. Li QH, Liu CL, Yan AH, et al. Analysis of hemorheology in 32 patients with Lyme disease serum antibody (IgG) positive. Clinical Focus. 2002(21):1283-1284.
213. Liu Y, Tan YH, Sun H, et al. An Seroepidemiological investigation on Lyme disease in Shawan forest region of Xinjiang, China. Chin J Vector Biol Control. 2002(01):53-54.
214. Sun Y, Xu RM, Zhang PH, et al. Isolation and identification of some common tick species of Lyme disease spirochetes in my country. Acta Parasitology et Medica Entomologica Sinica. 2002;09(02):114-119.
215. Tan YH, Liu Y, Sun H, et al. Clinical features of neurological manifestation of Lyme disease in Xinjing. Chin J Vector Biol Control. 2002(02):93-94.
216. Xing FK, Peng ZH, Gao GH, et al. Investigation of Lyme disease in Changping district, Beijing. Chin J Zoono. 2002(06):106-110.
217. Zhao HM, Su JY, Wan KL, et al. Epidemiological investigation of Lyme disease in Xilingole District of Inner Mongolia. Progress In Veterinary Medicine. 2002(02):72-74.
218. Cai SL, Dong CX, Qu SH. Seroepidemiological survey of Lyme disease among the population in Huairou District, Beijing. Chin J Vector Biol Control. 2003(06):424.
219. Dong ZS, Wang CS, Yang XJ, et al. Study on *Borrelia burgdorferi* infective of mouseand livestock in Jilin Province. Chin J Zoono. 2003(04):78-80.
220. Gu CG, Li YJ, Pan BS. Detection of Lyme disease spirochetes antibodies in the population of Heihe forest area by ELISA technology. Heilongjiang Medicine And Pharmacy. 2003(01):63.
221. He HJ, Huang XM. Investigation of Lyme disease spirochetes carried by Boophilus miniatures in southern Hunan. Chin J Vector Biol Control. 2003(04):303.
222. Li H, Zhang XG, Xin ZL, et al. Investigation on the prevalence of Lyme disease in the Great Khingan Mountains of Inner Mongolia. Chin J Epidemiol. 2003(08):1.
223. Li N, Cui BJ, Liu RC. Report of 3 cases of Lyme disease. Clinical Focus. 2003(16):956.
224. Li ZW, Wang JB, Yuan HY, et al. Seroepidemiological investigation and analysis of recessive infection of Lyme disease. Journal of Medical Pest Control. 2003(12):758-759.
225. Lu JM. Clinical Analysis of 17 Cases with Lyme disease displaying mainly neurological manifestations. Journal of Clinical Research. 2003(11):831-833.
226. Song CY, Jia YH, Chen JY, et al. A Seroepidemiological survey of Lyme disease infection among primary and middle school students in Ji County, Tianjin City. Chinese Journal of School Health. 2003(02):181.
227. Tian Z, Wan KL, Hou XX, et al. Seroepidemiological survey of Lyme disease among populations in Xinjiang petroleum exploration area. Chin J Epidemiol. 2003(08):1.
228. Wang ZX, Wang DM, Weng CR, et al. Investigation on Lyme disease in Guizhou province. Chin J Zoono. 2003(02):113-114.
229. Weng CR, Zhang ZF, Wang ZX, et al. Seroepidemiological survey of Lyme disease in Guizhou Province. Guizhou Medical Journal. 2003(05):474-475.
230. Xu X. Current status and Prevention of Lyme disease infection in workers of Henan oilfield test production company in Xinjiang. The First Academic Symposium of the Professional Committee of Zoonoses of Henan Preventive Medicine Association; Zhengzhou, China; 2003.
231. Yan XL. Seroepidemiology of Lyme disease pathogen and its distribution in Jiangsu. Chinese Journal of School Doctor. 2003(01):29-30.
232. Liu ZJ, Sun Y, Shi SZ, et al. Investigation on the natural foci of Lyme disease in Lazikou forest area. Chin J Zoono. 2004(05):445-447.
233. Meng XH, Liu ZJ, Huang FS. Study on tick as Lyme disease vector in China. Chinese Journal of Hygienic Insecticides & Equipments. 2004(03):137-141.
234. Quan XH. Experience in the treatment of 73 cases of Lyme disease. Journal of Zhengzhou University (Medical Sciences). 2004(04):727.
235. Shi SZ, Liu ZJ, Sun Y, et al. Investigation on small animals infection of *Borrelia burgdorferi* in Diebu forest. Chinese Journal of Hygienic Insecticides & Equipments. 2004(04):220-221.
236. Tian Z, Chen J, Wan KL. Study on the therapeutic effect of 175 patients with Lyme disease. China Preventive Medicine. 2004(01):3.
237. Wang JZ, Yuan P, Guo HL, et al. Preliminary epidemiological investigation of Lyme disease in Shiyan City. Journal of Hubei University of Medicine. 2004(06):370.
238. Yu DD. Molecular epidemiology of *Borrelia burgdorferi* in Lyme disease in Jilin area [Master Dissertation]. Changchun(Jilin): Jilin University; 2004.
239. Dong LY, Chen XN, Sun Y, et al. Analysis of zoonotic characters on lyme disease in forests of Chengde. Chin J Publ Heal. 2005(07):836-837.
240. Gong ZY, Jiang LP, Wang Z, et al. Serological epidemiology investigation of Lyme disease in Zhejiang Province. Dis Surveill. 2005(10):510-512.
241. Jin SW, Dong JJ, Tian Z, et al. Analysis on clinic type and therapeutic efficacy of 400 cases of Lyme disease. Dis Surveill. 2005(07):356-358.
242. Liu ZJ, Shi SZ, Zhang JJ, et al. Investigation on the combined infection of Lyme disease, hare fever, tsutsugamushi and Q fever in Diebu forest area. World Journal of Infection. 2005;05(01):45-47.
243. Lu HZ, Huang ZJ, Chen MQ, et al. A case of Lyme disease misdiagnosed as tuberculous meningitis. Chin J Infect. 2005(01):14.
244. Qi Y, Wang F. Clinical analysis of Lyme disease (report of 11 cases). Journal of Jilin University (Medicine Edition). 2005(01):20.
245. Sun HS, Tian Z, Geng Z, et al. An Investigation on Lyme disease in Xinjiang Prospecting Bureau of Henan Oilfield. Chin J Vector Biol Control. 2005(03):209-211.
246. Sun Y, Liu ZJ, Xu RM, et al. Effects of natural forests protection on the epidemical characteristics of Lyme disease. Acta Parasitology et Medica Entomologica Sinica. 2005(02):106-111.
247. Tan YH, Sun H, Liu Y, et al. Investigation on Different diagnostic techniques for Lyme disease. Chinese Journal of Clinical Neurosciences. 2005(03):269-272.
248. Wang CS, Dong ZS, Yang XJ, et al. First discovery of Lyme disease foci in Changchun area. Chin J Publ Heal Engineering. 2005(03):158-160.
249. Yang DL, Wang WM, Zeng YM, et al. The investigation on Lyme disease and the tick vectors and host animals in Quanzhou, Fujian. Strait J Prev Med. 2005(06):12-14.
250. Zhou XY. Clinical and epidemiological analysis of Lyme disease in Mudanjiang area from 1996 to 2003. Journal of Parasitic Biology. 2005(02):159.
251. Gao Y, He J, Wang JB, et al. Genotyping of *Borrelia burgdorferi* sensu lato isolated from Northeastern Forest Areas of China. Chin J Microbiol Immunol. 2006(05):404-408.
252. Huang HN, Ding Z, He J, et al. Investigation on spirochete infection of Lyme disease in Jilin forest region. National Symposium on Zoonotic Diseases; Beijing, China; 2006.
253. Ji ZH, Zhang FM, Wang BQ. One case of Lyme disease arthritis cure by combination of traditional Chinese and Western medicine. Chin J Integr Trad West Med. 2006(12):1106.
254. Jiang LP, Mo SH, Zheng SG, et al. Leptospira DNA of Lyme disease was first detected in tick in Zhejiang province. Chinese Journal of Health Laboratory Technology. 2006(01):30-31.
255. Liao YH. Collecting the medical history should carefully analyze the condition of the disease and respond to the system lessons from a case of Lyme disease misdiagnosed as tuberculous meningitis. New Medicine. 2006(02):116.
256. Liu MS, Wu YJ, Zhao ZF. Investigation on seroepidemiology of Lyme disease in Changzhi, Shanxi Province. Journal of Changzhi Medical College. 2006(01):17-18.
257. Liu ZJ, Sun Y, Shi SZ, et al. The impact of environmental changes on the spread of Lyme disease in the Western Development. World Journal of Infection. 2006;06(02):113-117,132.
258. Qu YG, Zhu Q, Yang ZJ, et al. Seroepidemiological investigation of Lyme disease in cattle and sheep in Xinjiang. The Second Meeting of the Sixth Council of the Animal Infectious Diseases Branch of the Chinese Society of Animal Husbandry and Veterinary Medicine and the Sixth Representative Conference of the Teaching Professional Committee; Daqing, China; 2006.
259. Wang HW, He J, Zhu CY, et al. Detection and genotyping of *Borrelia burgdorferi* sensu lato in ticks from some areas of China. Journal of Parasitic Biology. 2006(02):81-85.
260. Zhang JM, Yang HJ, Li XM, et al. Investigate the infection of Lyme disease in Yuanqu county. Chinese Remedies & Clinics. 2006(07):490-491.
261. Zhu CY, He J, Wang JB, et al. Investigation on *Borrelia burgdorferi* sensu lato in ticks and rodents collected in Da Xing-An Mountains Forest areas of China. Chin J Epidemiol. 2006(08):681-684.
262. Zhu CY, He J, Zhao QM, et al. Molecular epidemiological studies on *Borrelia burgdorferi* in rodents collected in the forest area of several provinces and autonomous regions of China. Chin J Zoono. 2006(09):817-820.
263. Cui WW, Deng J, Shi SF, et al. Investigation on the seroepidemiology of Lyme disease in Hangzhou. China Preventive Medicine. 2007(04):382-386.
264. Dong JH, Zhu JH, Yin FR. Investigation on the prevalence of Lyme disease in Daxinganling forest area. Practical Preventive Medicine. 2007(05):1457-1458.
265. Geng Z, Hou XX, Hao Q, et al. Anti-*Borrelia burgdorferi* antibody response in 827 patients with suspected Lyme disease. Chin J Vector Biol Control. 2007(03):219-221.
266. Hao Q, Yang XJ, Hou XX, et al. Investigation and genotyping of Lyme bacteria in Jilin province. Chin J Vector Biol Control. 2007(04):303-305.
267. He H, Hao Q, Hu MX, et al. Investigation on the vectors of *Borrelia burgdorferi* and on the identification of the isolates along China-Russia border in Eastern Heilongjiang province, China. Chin J Epidemiol. 2007(01):70-73.
268. Huang HN, Ding Z, Zhu CY, et al. Serological investigation on the coinfection of spotted fever group of Rickettsia and *Borrelia burgdorferi* among livestock in a forest area of Jilin, province. Chin J Zoono. 2007(02):146-148.
269. Ling F, Chen DF, Wang Z, et al. DNA fragments of Lyme disease spirochetes seized from mouse books in Zhejiang Province. Chin J Zoono. 2007(04):415-416.
270. Pei RD, Zhao ZQ, Wu XH. Investigation on the prevalence of Lyme disease in Meihekou City. Chinese Community Doctors. 2007(23):244.
271. Tan YH, Liu Y, Sun H, et al. Surveillance of Lyme disease in Xinjiang Uygur Autonomous Region During 2000-2004. Chinese Journal of Clinical Neurosciences. 2007(02):158-161.
272. Wang CS, Dong ZS, Xia QB, et al. Geographic distribution of Lyme disease in Jilin Province. Chin J Publ Heal Engineering. 2007(02):80-81+84.
273. Wang LJ, Hou XX, Chen ZL, et al. Seroepidemiological study of Lyme disease in Shandong province. Chin J Vector Biol Control. 2007(04):306-308.
274. Wang QC, Wu LX, Chen JH, et al. Investigation and analysis of Lyme disease in Qiannan area of Guizhou Province. Guizhou Medical Journal. 2007(07):657-658.
275. Ye XD, Zheng SG, Zheng HO, et al. First investigation of tick-borne Lyme disease in jinhua, Zhejiang Province. Acta Parasitology et Medica Entomologica Sinica. 2007(03):165-168.
276. Hao Q, Hou XX, Geng Z, et al. Studies on epidemiology and etiology of Lyme disease in China. The 2nd National Symposium on Zoonotic Diseases; Taizhou, China; 2008.
277. He H, Zhang GB, Hu MX, et al. Genospecies investigation and pathogen typing of Lyme disease spirochetes in the Sino-Russian border area of Heilongjiang. The 2nd International Forum on Sustainable Vector Control; Beijing, China; 2008.
278. Jiang LP, Zheng SG, Mo SH, et al. Detection and sequence analysis of *Borrelia burgdorferi* sensu lato DNA in rat-shape animals from Zhejiang province. Chin J Vector Biol Control. 2008(05):461-463.
279. Li YX, Wang SS, Zeng X, et al. Isolation and identification of *Borrelia burgdorferi* sensu lato in Leye county, Guangxi province. Chin J Epidemiol. 2008(12):1269-1270.
280. Li YX, Zeng X, Liao GH, et al. Investigation on natural foci of Lyme disease in Shangyao port of Nanning of Guangxi province. Chinese Journal of Health Laboratory Technology. 2008(07):1411-1413.
281. Liu FQ, Hao Q, Gao LD, et al. Preliminary epidemiological investigation of Lyme disease in Hunan province. Dis Surveill. 2008(06):337-340.
282. Liu Y, Chen J, Chen JY, et al. The discovery and study on the epidemic focus of Lyme disease in Tientsin, China. Chin J Zoono. 2008(03):257-259+262.
283. Liu Y, Geng Z, Chen JY, et al. Investigation on the separation of spirochetes of Lyme disease in patients with polyneuritis in Ji County, Tianjin City. Tianjin Medical Journal. 2008(05):345.
284. Liu ZJ, Sun Y, He J, et al. Study on the relation of transmission of Lyme disease and ecological protection from Western Regions in China. Chin J Epidemiol. 2008(05):449-454.
285. Meng Z, Jiang LP, Lu QY, et al. Detection of co-infection with Lyme spirochetes and Spotted fever group *rickettsiae* in a group of *Haemaphysalis longicornis*. Chin J Epidemiol. 2008(12):1217-1220.
286. Wang CS, Wan KL, Guo JH, et al. Epidemiological survey on Lyme disease in the frontier regions in Jilin province. Journal of Public Health and Preventive Medicine. 2008(05):48-49.
287. Wang MW. Epidemiological study of Lyme disease in six provinces of China [Master Dissertation]. Harbin(Heilongjiang): Harbin Medical University; 2008.
288. Zheng SG, Ye XD, Zheng HO, et al. Investigation of prevalence of tick-borne diseases in Jinhua District, Zhejiang Province. China Preventive Medicine. 2008(01):8-12.
289. Gu R, Shi SZ. Investigation and control of vector ticks of Lyme disease in Longnan area. Chinese Journal of Hygienic Insecticides & Equipments. 2009;15(04):337-338.
290. Hao GF, Li H, Sun Y, et al. Detection of tick and tick-borne pathogen in some ports of Inner Mongolia. Chin J Epidemiol. 2009(04):365-367.
291. Lin GY, Zhang SY, Shen JJ, et al. Retrospective investigation of 2 cases of clinical diagnosis of granulocytic anaplasmosis. Strait J Prev Med. 2009;15(05):31-32.
292. Wang CS, Wan KL, Yang XJ, et al. Epidemiological survey of Lyme disease in plain region of Jilin Province. Practical Preventive Medicine. 2009;16(02):341-342.
293. Wang CS, Wan KL, Yang XJ, et al. Investigation on the distribution of Lyme disease in Yanbian and Jilin. Practical Preventive Medicine. 2009;16(04):1074-1075.
294. Wang HY, Hou XX, Li LQ, et al. Seroepidemiological investigation of Lyme disease in Miyun county, Beijing in 2005. China Preventive Medicine. 2009;10(08):737-739.
295. Yu DS, Geng Z, Jiang JX, et al. Investigation on the natural focus of Lyme disease in Diebu county of Gansu province. Chin J Vector Biol Control. 2009;20(01):57-58.
296. Yue JN, Shi Y. Epidemiological investigation of Lyme disease in parts of forest areas in Qinghai province. Chin J Vector Biol Control. 2009;20(04):358-359.
297. Zhang F, Zhong ZH, Gong ZW, et al. Study on the sero-epidemiology of Lyme disease from certain areas of northwestern China. Chin J Epidemiol. 2009(12):1318-1319.
298. Zhu CY. Investigation and genotyping of *Borrelia burgdorferi* infection in vectors and hosts [Ph.D Dissertation]. Beijing: Academy of Military Medical Sciences; 2009.
299. Zhuang Y, Wang DM, Jiang WJ, et al. Survey of Lyme disease infection among rural population in Guizhou Province in 2006. Guizhou Medical Journal. 2009;33(02):169-170.
300. Bian CL, Gong ZD, Zhang LY, et al. Detection and sequence analysis of *Borrelia burgdorferi* sensu lato in dogs from north-western Yunnan province, China. China Preventive Medicine. 2010;11(08):757-759.
301. Feng XY, Zhang JJ, Liu ZJ, et al. Investigation on serum antibodies of Lyme disease spirochetes and Coxiella Q Fever in Yaks and goats in Qilian County, Qinghai Province. Acta Parasitology et Medica Entomologica Sinica. 2010;17(03):145-147.
302. Geng Z, Hou XX, Wan KL, et al. *Borrelia burgdorferi* antibody levels in 105 patients with suspected Lyme disease. Chin J Vector Biol Control. 2010(12):1346-1348.
303. Geng Z, Hou XX, Wan KL, et al. Isolation and identification of *Borrelia burgdorferi* sensu lato from ticks in six provinces in China. Chin J Epidemiol. 2010;21(01):65-67.
304. Gong ZW, Zhang F, Zhang JJ, et al. Coinfection by *Borrelia burgdorferi*, Francisella tularensis and Coxiella burnetii in domestic animals from Gansu province, China. Chin J Epidemiol. 2010(02):234-235.
305. Hou XX, Geng Z, Hao Q, et al. Rats, the primary reservoir hosts of *Borrelia burgdorferi*, in six representative provinces, China. Chin J Zoono. 2010;26(11):1034-1036.
306. Li YX, Wang ZX, Zeng X, et al. *Borrelia burgdorferi* isolated from *Ixodes sinensis* in the border areas between Guangxi and Guizhou in China. Chin J Vector Biol Control. 2010;21(03):238-240.
307. Li ZQ, Liu ZJ, Fei JX, et al. Investigation of Lyme disease natural epidemic focus in Shaanxi Province. Chinese Journal of Hygienic Insecticides & Equipments. 2010;16(05):356-359.
308. Li ZQ, Liu ZJ, Guo HL, et al. Report of 14 cases of Lyme disease found in Shaanxi Province. World Journal of Infection. 2010;10(01):29-31.
309. Lin H. Lyme disease:4 cases report. Chinese Journal of Convalescent Medicine. 2010;19(02):178-179.
310. Liu H, Zhang F, Gong ZW, et al. Detection and genotyping of *Borrelia burgdorferi* in ticks from Liupanshan area of Ningxia Hui Autonomous Region. Chin J Publ Heal. 2010;26(10):1282-1283.
311. Lv YN, Dou XF, Gan YD, et al. Report of 1 case of Lyme disease reported directly online for the first time in Beijing. Capital Journal of Public Health. 2010;4(06):277-279.
312. Meng Z, Jiang LP, Li ZL, et al. Inflection with Lyme spirochetes detected in rodents of Zhejiang middle area. Chinese Journal of Health Laboratory Technology. 2010;20(10):2503-2505.
313. Niu QL, Yang JF, Guan GQ, et al. Identification and phylogenetic analysis of Lyme disease *Borrelia spp.* isolated from Shangzhi Prefecture of Heilongjiang Province, China. Vet Sci China. 2010;40(07):661-666.
314. Tan Y, Niu XS, Na, et al. The efficacy of antibiotics in the therapy on different types and stages of Lyme disease. Beijing Medical Journal. 2010;32(06):462-463.
315. Tong CL. Isolation and identification of *Borrelia burgdorferi* from ticks and establishment of ELISA method for detection of Lyme disease [Master Dissertation]. Hefei(Anhui): Anhui Agricultural University; 2010.
316. Xu N. A case report of articular type of Lyme disease. Journal of Medical Pest Control. 2010;26(04):375.
317. Yang JF. Research on three detection techniques of *Borrelia burgdorferi* [Master Dissertation]. Beijing: Chinese Academy of Agricultural Sciences; 2010.
318. Zheng ZL, Gu SP, Huang Y, et al. Detection of *Borrelia burgdorferi* sensu lato DNA in rodents from Anji. Chinese Journal of Health Laboratory Technology. 2010;20(11):2844-2845.
319. Li ZQ, Luo F, Liu ZJ, et al. Preliminary investigation of rodents and storage host of pathogen of Lyme disease in Shaanxi. Journal of Medical Pest Control. 2011;27(06):491-495.
320. Luo XH, He ZP. Natural infection of 4-Lyme disease *Borrelia burgdorferi* in Diebu and Huajian areas of Gansu province. Chinese Journal of Endemiology. 2011(05):557-558.
321. Shi SG, Zhang F, Liu ZJ. Molecular epidemiological studies on *Borrelia burgdorferi*i in ticks collected from several provinces and autonomous regions of northwestern China. Chin J Zoono. 2011;27(05):461-463.
322. Tan YH, Liu Y, Sun H, et al. Surveillance and analysis on the natural foci of Lyme disease in Southern Mountainous Area of Urumqi, Xinjiang. Chin J Vector Biol Control. 2011;22(02):141-143.
323. Tong CL, Wu YJ, Zhou YZ, et al. Expression on specific fragment of flagellin from *Borrelia burgdorferi* and its diagnostic. Chin J Zoono. 2011;27(12):1106-1110.
324. Yang JF, Yin H, Li YQ. Epidemiological survey of *Borrelia burgdorferi* in ticks in some areas of my country. The 26th Annual Reproductive Conference of China Dairy Association and the 3rd National Cattle Disease Prevention and Control Symposium of the National Beef and Yak/Dairy Cattle Industrial Technology System; Lanzhou, China. 2011.
325. Yao MQ, Shi ML, Xia XW, et al. An epidemiological investigation report of Lyme disease. Endemic Diseases Bulletin (China). 2011;26(06):50-51.
326. Yuan GX, Jin LH, Cai ZA. The first phase clinical observation of doxycycline in the treatment of Lyme disease. China Practical Medica. 2011;6(26):142-143.
327. Zhang JJ, Liu ZJ, Zhang F, et al. Molecular epidemiology investigation of spirochetes for Lyme disease in Altay region. Acta Parasitology et Medica Entomologica Sinica. 2011;18(01):34-37.
328. Zhang XX, Niu JG, Zhang XY. Investigation on Lyme disease infection in mountain forest Area of Jincheng City. International Journal of Medical Parasitic Diseases. 2011;38(01):17-18.
329. Dong YH, Zhao JY, Lu X, et al. Serological epidemiology investigation of Lyme disease in Henan Province. Modern Preventive Medicine. 2012;39(14):3681-3682.
330. Mou HG, Gu P. A case of Lyme disease with orthostatic hypotension misdiagnosed as diabetic cardiovascular autonomic neuropathy. Acta Acad Med Mil Tert. 2012;34(15):1542+1559.
331. Tan YH, Liu Y, Sun H, et al. Evaluation of the detection of *Borrelia burgdorferi* DNA in urine samples by polymerase chain reaction for diagnosing Lyme disease. Journal of Chinese Physician. 2012;20(04):372-376.
332. Zhou XP, Li YP. Molecular epidemiological studies on natural infection of Lyme disease spirochaete in rodents. Chinese Journal of Hygienic Insecticides & Equipments. 2012;18(05):408-410.
333. Chen Q, Sun H, Fei DL, et al. Seroepidemiological investigation of canine Lyme disease in western Liaoning. Chinese Journal of Veterinary Medicine. 2013;49(08):65-66.
334. Geng Z. Research on the establishment and preliminary application of standardization technique of pulsed field gel electrophoresis for Lyme disease [Ph.D Dissertation]. Changchun(Jilin): Jilin University; 2013.
335. Han H, Wan DZ, Zhang XL, et al. Detection and genotyping of *Borrelia burgdorferi* sensu lato in Pingxiang of Guangxi Zhuang Autonomous Region, China. Chin J Vector Biol Control. 2013;24(03):244-246.
336. Li X, Yang ZW, Wang WY, et al. Analysis of 40 cases of Lyme disease. Chron Pathematol J. 2013;14(09):671-674.
337. Li Y, Kang FY, Yang JF, et al. Co-infection of *phagocytic anaplasma* and *Borrelia burgdorferi* in ticks in Gansu, Hunan and Guangdong provinces. Chin J Zoono. 2013;29(02):117-121.
338. Sun X, Zhang GL, Liu XM, et al. Investigation of tick species and tick-borne pathogens in Hoxud county of Xinjiang Uyghur Autonomous Region, China. Chin J Vector Biol Control. 2013;24(01):5-7+10.
339. Tan YH, Liu Y, Wan KL, et al. Molecular epidemiological study of *Borrelia burgdorferi* infection among population in Xinjiang, China. Chin J Vector Biol Control. 2013;24(04):297-300.
340. Zhou X. Establishment and research of MLVA classification method for spirochetes of Lyme disease in China [Master Dissertation]. Taiyuan(Shanxi): Shanxi Medical University; 2013.
341. Zhou X, Hou XX, Geng Z, et al. Application of multiple-locus VNTR analysis and multi-locus sequence analysis in genotyping of *Borrelia burgdorferi* sensu lato. Chin J Vector Biol Control. 2013;24(02):98-102.
342. Geng Z, Li GH, Hou XX, et al. The first case of Lyme disease in Shanxi province, China: a case study. Chin J Vector Biol Control. 2014;25(04):318-319+322.
343. Gu CG, Cao XH, Jia YP, et al. Seroepidemiological investigation of Lyme disease in a part of forest region of Xiao Hinggan Mountains. Modern Preventive Medicine. 2014;41(06):1125-1126+1129.
344. Li J, Tong SF, Yang ZF, et al. Lyme disease and Scrub Typhus serosurvey among college freshmen in Yunnan Province. Journal of Kunming Medical University. 2014;35(05):21-23.
345. Ma RL, Wang Y, Wang XR, et al. Seroepidemiological investigation of Lyme disease in yak and sheep in Qinghai Province. China Animal Health Inspection. 2014;31(06):62-64.
346. Wu Q. Study on the transmission vector and pathogenic characteristics of *Borrelia garinii* SZ strain of Lyme disease [Ph.D Dissertation]. Beijing: Chinese Academy of Agricultural Sciences; 2014.
347. Zhang L, Hou XX, Geng Z, et al. Nested-PCR and LAMP in detection of *B. burgdorferi* in ticks. Chin J Zoono. 2014;30(12):1192-1195.
348. Zhang L, Wang YZ, Chen CF, et al. Isolation of *Borrelia burgdorferi* in *Ixodes* from four counties, in North Xinjiang. Chin J Epidemiol. 2014;35(03):262-265.
349. Zhao Y, Liu R, Zhang GL, et al. Seroepidemiological survey of tick-borne diseases in a border defense force in the north of Xinjiang. J Prev Med Chin Peopl Liber Army. 2014;32(04):324-325.
350. Cheng C, Ju WD, Fu WM, et al. Investigation on the compound infection of three tick-borne infectious diseases in vector ticks at Mishan Port in Heilongjiang. Chin J Publ Heal Management. 2015;31(03):361-363.
351. Dong JY. Identification of tick species and investigation of tick-borne diseases in northern Xinjiang [Master Dissertation]. Shihezi(Xinjiang): Shihezi University; 2015.
352. Dong JY, Wang AD, Mou LM, et al. The genospecies of *Borrelia burgdorferi* sensu stricto was detected for the first time in Hyalopia asiatica in Karamay, Xinjiang. Chin J Epidemiol. 2015;36(05):539-540.
353. Lan YQ, Ling F, Huang JB, et al. Surveillance of tick-borne infections during 2012-2013 in Lishui, Zhejiang. Chin J Vector Biol Control. 2015;26(05):512-515.
354. Liu XM, Zhang GL, Liu R, et al. Study on co-infection of tick-borne pathogens in *Ixodes persulcatus* in Charles Hilary, Xinjiang Uygur autonomous region. Chin J Epidemiol. 2015;36(10):1153-1157.
355. Wang AD, Xu J, Wang YZ, et al. Detection of Lyme disease pathogens in isolated ticks at Aibi Lake, Alataw Pass, Xinjiang Autonomous Region. Chinese Journal of Endemiology. 2015;34(08):592-595.
356. Wang XR, Liu ZJ, Ma RL, et al. Epidemiological survey of animal Lyme disease in six counties in Qinghai Province. Chinese Journal of Veterinary Medicine. 2015;51(01):55-56.
357. Yang LT, Gao ZH, Zhao WH, et al. Preliminary investigation of infection situation of *Borrelia burgdorferi* sensu lato in host animals in Deqin county of Yunnan province. China Tropical Medicine. 2015;15(04):419-421+433.
358. Yu PF, Niu QL, Liu ZJ, et al. Investigation of 6 pathogens in ticks in five provinces in my country. The 13th Symposium of the Chinese Society of Animal Husbandry and Veterinary Medicine Veterinary Parasitology; Harbin, China; 2015.
359. Zhang JJ, Zhang F, Li L, et al. *Borrelia burgdorferi* infection and their genospecies in ticks and rodents collected from Maijishan region, Gansu Province, China. Chin J Zoono. 2015;31(04):357-360.
360. Zhang L, Shi Y, Geng Z, et al. Investigation of *Borrelia burgdorferi* in Glires in Huzhu, Zekog, and Qilian county, Qinghai. Chin J Vector Biol Control. 2015;26(02):148-150.
361. Zhu X, Zhang L, Hou XX, et al. Investigation on Lyme disease of patients with arthritis or neurological disorders in Hainan Province, China. Chin J Zoono. 2015;31(04):353-356.
362. Chen QY. Molecular epidemiological investigation of ticks and tick-borne pathogens in domestic animals in kazak autonomous prefecture of Ili, Xinjiang, China [Master Dissertation]. Beijing: Chinese Academy of Agricultural Sciences; 2016.
363. Chen YL, Yang YS, Jia LL, et al. Seroprevalence of Lyme disease in Miyun County of Beijing, China. Capital Journal of Public Health. 2016;10(02):83-85.
364. Dong CH, Yin JX, Zuo SQ, et al. Pathogen infection of small mammals from households in Western Yunnan Province. Chin J Zoono. 2016;32(07):623-631.
365. Guo G. Study on pathogen carrying and genetic characteristics of rodents and ectoparasites in northern Xinjiang [Ph.D Dissertation]. Urumchi(Xinjiang): Xinjiang Medical University; 2016.
366. Han H, Yang Y, Tan KW, et al. Analysis on risk of tick-borne pathogens at Zhangmu port in Tibet. Chin J Vector Biol Control. 2016;27(06):539-541.
367. Hu G, Fang ZQ, Wang JC, et al. Investigation of pathogens carried by ticks at Zhangmu port on the border between China and Nepal. Acta Parasitology et Medica Entomologica Sinica. 2016;23(03):164-168.
368. Li YP, Pang ZD, Liu ZJ. Study on tick-borne disease in Northwest of China. Chinese Journal of Hygienic Insecticides & Equipments. 2016;22(02):180-183.
369. Liu W. Study on detection technology of Lyme disease spirochete recombinant specific antigen and RPA [Master Dissertation]. Beijing: Chinese Center for Disease Control and Prevention; 2016.
370. Luo JJ, Wu XF, Lu QX, et al. Investigation on Lyme disease and vector insect carriers of Various Llvestocks in Dulan County, Qaidam Basin. Chinese Qinghai Journal of Animal and Veterinary Sciences. 2016;46(02):27-28.
371. Mou LM. Establishment and epidemiological investigation of Lyme disease and Brucellosis diagnosis methods [Master Dissertation]. Shihezi(Xinjiang): Shihezi University; 2016.
372. Sun X, Zhang GL, Zheng Z, et al. Serological investigation of tick-borne diseases among workers in forest areas in northern Xinjiang. Acta Parasitology et Medica Entomologica Sinica. 2016;23(03):158-163.
373. Wang AD. Background investigation of common vector organisms at Alashankou port and study on the detection of pathogen spectrum [Master Dissertation]. Shihezi(Xinjiang): Shihezi University; 2016.
374. Wu JY, Huang ZW, Kang Q, et al. Seroepidemiological survey on *Borrelia burgdorferi* infection of dogs in Nanjiang region of Xinjiang. Journal of Inner Mongolia Agricultural University (Natural Science Edition). 2016;37(06):1-4.
375. Yin XP, Chen Q, Wang J, et al. Investigation on *Borrelia burgdorferi* in rodents captured at Alashankou port, Xinjiang. Chinese Journal of Frontier health and Quarantine. 2016;39(04):269-271.
376. Yin XP, Tian YH, Liang Z, et al. Analysis of sequencing results of rodent and tickspathogen positive genes at Alashan Pass Port on the China-Kazakhstan border. China's scientific and technological achievements. 2016;17(18):40-43.
377. Zhang F, Wang W, Li L. *Borrelia burgdorferi* infection and genospecies in ticks and rodents collected in Qinghai Province. J Prev Med Chin Peopl Liber Army. 2016;34(06):799-802.
378. Zhang L, Hou XX, Liu HX, et al. Prediction of potential geographic distribution of Lyme disease in Qinghai province with Maximum Entropy model. Chin J Epidemiol. 2016;37(01):94-97.
379. Bian C, Liu CY, Zhang T, et al. Analysis of multiple infections of Tick-borne Diseases Forest Encephalitis, Lyme disease and Spotted Fever. World Latest Medicine Information. 2017;17(17):137.
380. Chang QC, Fu X, Guo DH, et al. Investigation report on pathogens carried by ticks in Wandashan forest area. The 16th National Academic Conference of the Chinese Society of Parasitology and the 7th International Symposium on Parasitology; Jiujiang, China; 2017.
381. Cheng C. Investigation on the diversity and compound infection of tick-borne pathogens at Heilongjiang Port [Master Dissertation]. Beijing: Academy of Military Medical Sciences; 2017.
382. Feng JX, Yang YY, Li JJ, et al. Epidemiological investigation of *Borrelia burgdorferi* in Siping city of Jilin province surrounding areas and expression of surface protein C (OspC) from *Borrelia burgdorferi*. Chinese Journal of Veterinary Science. 2017;37(11):2151-2155.
383. Guo Q, Tian F, Kabunudong M, et al. Investigation on rats and pathogens at Huoerguosi port regions in Xinjiang, 2016. Chinese Journal of Frontier health and Quarantine. 2017;40(04):276-277+289.
384. Hou XX, Zhang L, Li GH, et al. Investigation on natural foci of Lyme disease in Lyuliang area, Shanxi province. Chin J Vector Biol Control. 2017;28(06):550-552.
385. Ji CL. Investigation on the foci of tick-borne Lyme disease in Alatanheli area of Baogeda Mountain. Journal of Clinical Medical Literature. 2017;4(45):8886+8888.
386. Li HY, Zhao SS, Zhang L, et al. Molecular investigation on the genera of Anaplasma and *Borrelia* in north region of Xinjiang. Journal of Shihezi University (Natural Science. 2017;35(01):108-112.
387. Long J, Niu XS, Wen J, et al. The survey of *Borrelia burgdorferi* infection among blood donors in Urumqi area. Chin J Vector Biol Control. 2017;28(03):280-282.
388. Pan YP, Yang JF, Niu QL, et al. Study on *Borrelia burgdorferi* sensu lato and spotted fever group Rickettsia in *Ixodes persulcatus* in Heilongjiang Province. Vet Sci China. 2017;47(01):31-37.
389. Tian XJ, Li SG, Wu ZY, et al. Investiga Investigation on Lyme disease among 1890 suspect patients in Beijing. Acta Parasitology et Medica Entomologica Sinica. 2017;24(03):155-160.
390. Wan DZ, Yang HM, Liang L, et al. Distribution and pathogens of natural infection on rodents in China-Vietnam border ports. Chinese Journal of Frontier health and Quarantine. 2017;40(05):341-344.
391. Wu JY, Gong TM, Wang L, et al. Investigation on serum epidemiology of tick-born Lyme disease in different population in Kashi city of Xinjiang. Journal of Tropical Medicine. 2017;17(12):1663-1665+1673.
392. Yan FF, Chang W, Pan LX, et al. Seroepidemiological survey of animal tick-borne Lyme disease in Awati County, Xinjiang. Heilongjiang Animal Science and Veterinary Medicine. 2017(08):114-115.
393. Yang YS, Lv YN, Chen YL. Serological investigation in arthritis patients with Lyme disease in Miyun county, Beijing. Chin J Vector Biol Control. 2017;28(05):490-491.
394. Zhang WZ, Wang XR. Epidemiological investigation of Lyme disease in some areas of Qinghai Province. Chinese Qinghai Journal of Animal and Veterinary Sciences. 2017;47(02):37-40.
395. Chen T, Zhu X, Zhang L. Analysis of Serum Lyme disease screening in patients with joint symptoms in Sanya. The 13th National Conference on Immunology; Shanghai, China; 2018.
396. Han R. Study on the Diversity of *Ixodes* in Qinghai Province and the Genetic Polymorphism of Tick-borne Pathogens [Ph.D Dissertation]. Beijing: Chinese Academy of Agricultural Sciences; 2018.
397. Qin LX, Zhang Q. Investigation on the infection of *Borrelia burgdorferi* carried Dermacentor sp. in Yichangerkuang and Xinghuo pasture in Hailun City of Heilongjiang Province. Feed Review. 2018(08):53-57.
398. Song CL, Hu Y, Tang L, et al. Analysis of microbial diversity carried by Dermacentor silvarum through next-generation sequencing. Heilongjiang Animal Science and Veterinary Medicine. 2018(21):5-9+254.
399. Wu LTY, Gao W, Yin XH, et al. Survey on *Borrelia* in rodents from Bayannur, Inner Mongolia Autonomous Region, China. Chin J Vector Biol Control. 2018;29(03):239-241.
400. Yang Y, Gao YF, Cao S, et al. Investigation on tick-borne pathogens in Inner Mongolia Manchuria port area during 2012-2014. Chin J Vector Biol Control 2018. 2018;29(02):147-150+156.
401. Zhang L, Miao GQ, Hou XX, et al. Evaluation of nested PCR and real-time PCR in host surveillance of Lyme disease. Chin J Vector Biol Control. 2018;29(05):425-427.
402. Dong CJ, Guo Y, Dong SS, et al. Primary investigation on the infection of *Borrelia burgdorferi* in rats of Jianchuan County, Yunnan. China Tropical Medicine. 2019;19(01):66-69.
403. Dong CJ, Guo Y, Hou XX, et al. Investigation of vector and host infection of Lyme disease in Jinghong, Yunnan. Dis Surveill. 2019;34(03):246-250.
404. Dong XD, He ZH, Gao ZH, et al. Detection and identification of *Borrelia sinica* in *Ixodes ovatus* from the border region of Yunnan province, China. Chin J Vector Biol Control. 2019;30(05):519-523.
405. He ZH. Molecular Epidemiological investigation of *Borrelia burgdorferi* infected by host animals and vector ticks in western Yunnan [Master Dissertation]. Dali (Yunnan): Dali University; 2019.
406. Hu GW, Shen YL, Zhao QB, et al. Detection of Lyme disease in cattle, sheep and vectors in some areas of Qinghai. The First Young Scientist Academic Forum of the Chinese Society of Animal Husbandry and Veterinary Medicine Veterinary Parasitology Branch; Wuhan, China; 2019.
407. Li JQ, Yuan YY, Wang YX, et al. A Case of Lyme disease with IgA nephropathy. The Chinese Journal of Dermatovenereology. 2019;33(04):453-455.
408. Liu WJ, Xiao FZ, Lin SX, et al. An investigation of rodents infected with *Borrelia burgdorferi* in five counties (cities) of Fujian province, China. Chin J Vector Biol Control. 2019;30(03):320-323.
409. Liu ZQ. Tick species distribution, molecular characteristics and molecular detection of important tick-borne pathogens in northern Xinjiang [Ph.D Dissertation]. Shihezi(Xinjiang): Shihezi University; 2019.
410. Meng Y, Zhang X, Wang DF, et al. Epidemiological analysis of the first confirmed case of Lyme disease combined with Spotted fever in Beijing. Chin J Zoono. 2019;35(01):81-84.
411. Wang Y, Ye F, Liu LY, et al. Establishment of a TaqMan-MGB probe real-time PCR assay for detection of *Borrelia burgdorferi* in ticks in Xinjiang. China Animal Health Inspection. 2019;36(11):89-94+99.
412. Li B, Lu S, Luo T, et al. Infection status of pathogens in Citellus undulatus in Guertu, Xinjiang. Dis Surveill. 2020:5.
413. Zhu X, Hou XX, Yu L, et al. Serological investigation on Lyme disease in western region of Hainan Province. Chin J Zoono. 2020;36(04):313-316+319.
414. Bhatt S, Gething PW, Brady OJ, et al. The global distribution and burden of dengue. Nature. 2013;496(7446):504-507.
